# Functional analysis of two sterol regulatory element binding proteins in *Penicillium digitatum*

**DOI:** 10.1371/journal.pone.0176485

**Published:** 2017-05-03

**Authors:** Ruoxin Ruan, Mingshuang Wang, Xin Liu, Xuepeng Sun, Kuang-Ren Chung, Hongye Li

**Affiliations:** 1 Institute of Biotechnology, Zhejiang University, Hangzhou, China; 2 Department of Plant Pathology, National Chung-Hsing University, Taichung, Taiwan; Seoul National University, REPUBLIC OF KOREA

## Abstract

The sterol regulatory element binding proteins (SREBPs) are key regulators for sterol homeostasis in most fungi. In the citrus postharvest pathogen *Penicillium digitatum*, the SREBP homolog is required for fungicide resistance and regulation of *CYP51* expression. In this study, we identified another SREBP transcription factor PdSreB in *P*. *digitatum*, and the biological functions of both SREBPs were characterized and compared. Inactivation of *PdsreA*, *PdsreB* or both genes in *P*. *digitatum* reduced ergosterol contents and increased sensitivities to sterol 14-α-demethylation inhibitors (DMIs) and cobalt chloride. Fungal strains impaired at *PdsreA* but not *PdsreB* increased sensitivity to tridemorph and an iron chelator 2,2’-dipyridyl. Virulence assays on citrus fruit revealed that fungal strains impaired at *PdsreA*, *PdsreB* or both induce maceration lesions similar to those induced by wild-type. However, Δ*PdsreA*, Δ*PdsreB* or the double mutant strain rarely produce aerial mycelia on infected citrus fruit peels. RNA-Seq analysis showed the broad regulatory functions of both SREBPs in biosynthesis, transmembrane transportation and stress responses. Our results provide new insights into the conserved and differentiated regulatory functions of SREBP homologs in plant pathogenic fungi.

## Introduction

Green mold, caused by *Penicillium digitatum* (Pers.: Fr.) Sacc., is the most destructive postharvest disease of citrus fruits. The disease causes severe losses of citrus fruits during packaging, storage, transportation, and marketing [1,2]. Application of sterol 14-α-demethylation inhibitors (DMIs), such as imazalil and prochloraz, is the most effective and economical method for green mold control. However, control efficacy of DMI fungicides is compromised as resistant strains emerged worldwide [3,4,5,6,7].

DMI fungicides inhibit the activity of the cytochrome P450-dependent sterol 14α-demethylase (Cyp51) and thus, block C14-demethylation of lanosterol in fungal pathogens [8,9]. DMI resistance has been reported in many phytopathogenic fungi, and major mechanisms leading to resistance include point mutations or overexpression of *CYP51* genes and overexpression of several transporter protein-coding genes [10,11,12,13,14,15,16,17,18]. In *P*. *digitatum*, overexpression of the *CYP51* genes is the dominant mechanism of DMI resistance in the field. Genotypes of these resistant isolates have been characterized, revealing that the insertion of a 126-bp tandem repeat or a 199-bp sequence in the promoter region of *CYP51A* or *CYP51B* leads to overexpression of these genes and thus, result in an increased resistance to DMI fungicides in *P*. *digitatum* [6,15,19,20]. Recently, a sterol regulatory element binding protein (SREBP) *sreA* was reported to be required for the resistance to prochloraz and the regulation of expression of *CYP51s* in *P*. *digitatum* [21].

SREBPs are key regulators for sterol homeostasis in many eukaryotes [22]. They are originally identified in mammals, which have two SREBP genes encoding three membrane-bound isoforms [23]. SREBP proteins contain a conserved basic helix-loop-helix (bHLH) DNA binding domain at the N-terminus, which has a unique tyrosine residues distinguished from other bHLH domains [24]. Although homologs of SREBPs have been identified in a number of fungal species, their biological functions could vary among different species. In most fungi, SREBPs play a vital role in the regulation for ergosterol biosynthesis [25]. In addition, SREBPs have been demonstrated to be crucial for hypoxia adaptation in *Schizosaccharomyces pombe*, *Cryptococcus neoformans*, and *Aspergillus fumigatus* [25]. In the rice blast fungus *Magnaporthe oryzae*, a SREBP homolog-coding gene (*MoSRE1*) has recently been reported to be associated with hypoxic response, conidiation, and invasive growth within host cells, but loss of *MoSRE1* did not cause remarkable decrease in disease severity [26]. Two SREBP homologs (*srbA* and *srbB*) have been characterized in *A*. *fumigatus*. *srbB* co-regulates the genes involved in heme biosynthesis and ergosterol biosynthesis in *A*. *fumigatus* [27]. Besides, *A*. *fumigatus srbB* independent of *srbA* was also involved in carbohydrate metabolism, indicating divergent functions between the two SREBP homologs. *P*. *digitatum* has two SREBP homologs designated PdSreA and PdSreB. The objective of this study is to determine the biological roles of *PdsreB* in *P*. *digitatum*, and to further understand its relationship with *PdsreA*.

## Materials and methods

### Fungal strains and growth conditions

The wild-type strain PdW03 (CBS130527) of *P*. *digitatum* was single spore collected from a diseased citrus fruit in Quzhou (118°50'N, 29°19'E) of Zhejiang province, China [6] and used for genetic manipulation. The citrus orchard was publicly owned and no permits were required for collecting samples at the location. Fungal strains were cultured on potato dextrose agar (PDA) medium at 25°C. Mycelia of wild-type and its derived mutants were cultured in potato dextrose broth (PDB) with agitation (160 rpm) at 25°C when DNA or RNA purification was desired. Conidia were harvested from fungal colonies cultured on PDA at 25°C by flooding with sterile water.

### Generation of gene deletion mutants

The *PdsreA* deletion vector, designated pTFCM-*PdsreA*-Del, was constructed by inserting truncated *PdsreA* fragments into the left and right arms of the *hph* gene conferring hygromycin resistance in the pTFCM vector [28]. The 5’-end *PdsreA* fragment (1,472 bp) was amplified by PCR from PdW03 genomic DNA with primer pair P1/P2 (S1 Table), digested with *Kpn*I and *Sac*I, and cloned into the *Kpn*I-*Sac*I digested pTFCM vector to generate plasmid pTFCM-*PdsreA*-up. The 3’-end *PdsreA* fragment (1,217 bp) was amplified using primer pair P3/P4, digested with *Spe*I and *Xho*I, and cloned into pTFCM–*PdsreA*-up to generate the deletion plasmid pTFCM-*PdsreA*-Del. The deletion construct was transformed into *Agrobacterium tumefaciens* strain AGL-1 and into *P*. *digitatum* via *A*.*tumefaciens*-mediated fungal transformation (ATMT) as described previously [28]. Putative transformants were selected from PDA medium supplemented with hygromycin B (50 mg/l). PCR diagnosis using the primers not present in the disruption construct was performed to identify putative mutants. Successful integration of the *hph* gene within *PdsreA* was confirmed further by Southern blot hybridization (S1 Fig). *PdsreB* was deleted in PdW03 using a similar strategy. A *neo* gene cassette conferring resistance to neomycin was used as a dominant selectable marker to generate double-deletion mutants from a Δ*PdsreA* mutant. The *PdsreA* and *PdsreB* double mutant strain (ΔΔ*PdsreAB*) were generated by transferring a pCA-neo-*PdsreB* deletion construct into Δ*PdsreA* via ATMT. Transformants were recovered from medium supplemented with neomycin (100 mg/l).

### Genetic complementation

The defective phenotypes observed in the *PdsreA* deletion mutant were restored by transforming a functional copy of the wild-type *PdsreA* gene. A 4425-bp full-length *PdsreA* gene, including a 1028-bp promoter region and a 227-bp terminator region, was amplified from genomic DNA of wild-type strain PdW03 using primers P29 and P30 (S1 Table). Restriction enzymes *Hind*III and *Kpn*I were incorporated into the primers to facilitate cloning. The PCR fragment was cloned into *Hind*III and *Kpn*I sites of the plasmid pCA-neo [29] to generate pCA-neo-CP*PdsreA*. The pCA-neo-*PdsreA* plasmid was transformed into a Δ*PdsreA* via ATMT. Transformants were recovered from medium supplemented with neomycin (100 mg/l), examined by PCR, and confirmed further by Southern blot analysis. Similarly, a functional copy of *PdsreB* was cloned into pCA-neo to yield pCA-neo-CP*PdsreB*, which was used to complement the phenotypes seen in the *PdsreB* deletion mutant.

### Assays for vegetative growth and fungicide sensitivity

Radial growth of the wild-type and the deletion mutant strains was assessed on PDA plates supplemented with the test chemicals. Those included 2,2’-dipyridyl (2DP), CoCl_2_, menadione, five DMI fungicides (imazalil, prochloraz, tebuconazole, fluconazole, and miconazole), and two fungicides (terbinafine and tridemorph) functioning as ergosterol biosynthesis inhibitors (EBIs) at different concentrations. Each plate was inoculated with a 5-mm agar plug carrying fungal mycelium taken from a 1-day-old culture as described previously [30]. Each treatment contained three replicates and all experiments were repeated at least two times. After incubation at 25°C for 5 days, the mean diameter of each colony was measured. The original mycelial plug diameter (5 mm) was subtracted from each measurement. The percentage of the mycelial radial growth inhibition (MRGI) was calculated using the formula: MRGI% = [(C − N)/(C − 5)]× 100%, where C is colony diameter of untreated mycelia and N is that of treated mycelium. The 50% effective concentration (EC_50_) values were calculated with the IBM SPSS Statistics 20.

### Quantification of ergosterol

For ergosterol extraction, spore suspensions (10^7^ spores/ml, 200 μl) of fungal strains were added into a 150-ml yeast glucose (YG) medium (5 g/l yeast extract, 15 g/l glucose) and incubated at 25°C for 2 days on a rotary shaker. Mycelia were harvested by passing through a filter paper and washing three times with sterile water. Total ergosterol was extracted by hexane using a previously described method [31]. Ergosterol samples were analyzed using Agilent 1100 high-performance liquid chromatography (HPLC) system. Ergosterol was separated in a Hypersil BDS C18 (250 nm × 4.6 nm, 5 mm) analytical column at 25°C using 100% methanol (chromatography pure) as mobile phase and detected at 282 nm wavelength. The identity of ergosterol was verified based on the retention time and co-chromatography of a commercially available ergosterol (Purity >85%, Sangon Biotech, China). The experiment was repeated twice.

### Virulence assays

Infection assays were performed on fruit of mandarin (*Citrus unshiu*) harvested from an organic orchard, which was never treated with fungicides, in Taizhou, China [31]. Conidia were harvested from 5-day-old cultures of *P*. *digitatum* strains grown on PDA and resuspended in distilled water, and the concentrations were adjusted to 10^6^ conidia/ml. Citrus fruits were washed with water before inoculation. Citrus peel was wounded (1–2 mm deep) with a bunch of 5 sterile needles and inoculated with 5 μl conidial suspensions. The infected citrus fruits were incubated at room temperature. Disease development of the inoculated citrus fruits was examined daily and the lesion size was measured at 4 days post inoculation. Twenty fruits were used for each strain. The experiment was repeated three times.

### Gene expression profiling and qRT-PCR analysis

Conidia of wild-type and mutant strains were harvested, inoculated in 100 ml PDB, and incubated at 25°C for 48 h. Fungal mycelia were harvested by passing through a filter paper, frozen in liquid nitrogen and lyophilized. Fungal RNA was extracted from dried mycelium using an AxyPrep^TM^ Multisource Total RNA Miniprep kit (Axygen Biotechnology, Hangzhou, China) according to the manufacturer’s instructions.

RNA-Seq libraries and paired-end sequencing using the Illumina Hiseq2500 platform were performed by the Biomarker Company (Beijing, China). After quality control, sequence reads were mapped to the genome of *P*. *digitatum* strain PHI26 [32] using bowtie2. Differentially expressed genes (DEGs) set was calculated with EBseq under the criteria of false discovery rate (FDR) <0.01 and fold changes >2 [33,34,35]. The DEGs were annotated by blast searching against non-redundant protein sequences (nr) and Swiss-Prot databases and then subjected to the FungiFun2 web-based sever to interrogate the functional enrichment of FunCat and gene ontologies [36]. RNA-seq data were deposited in NCBI's Sequence Read Archive (SRA) with accession number SRP073233. For quantitative real-time PCR, RNA sample (10 μg) was used for reverse transcription with a PrimeScript^TM^ RT reagent kit (TakaRa Biotechnology, Dalian, China). qRT-PCR was performed as described previously [31]. Primers used in this study are listed in S1 Table.

### Statistical analysis

The significance of treatments was determined by analysis of variance and treatment means separated based on the Tukey’s honestly significant difference available in the SPSS package (SPSS Inc., Chicago IL, USA). Means in the same column followed by the same letter are not significantly different.

### Ethics statement

No ethical permissions were required for this work which involved no experimentation involving animals or human samples.

## Results

### Identification and characterization of SREBPs in *P*. *digitatum*

A phylogenetic tree constructed using ribosomal proteins revealed a close relationship between *A*. *fumigatus* and *P*. *digitatum* (S2 Fig). From the complete genome sequence of *P*. *digitatum* [32], sequence search using *A*. *fumigatus* SrbB (AFUB_099590) as a query identified one homolog, PDIG_67800, with 53% identity, which was named *PdsreB*. *PdsreB* had a shorter ORF than *PdsreA* (PDIG_90150) [21], with 947-bp interrupted by a 56-bp intron, which encodes a polypeptide of 296 amino acids. PdSreB was found to have a bHLH domain in the C terminus. However, no DUF2014 domain was found in PdSreB, which was found at the C terminus of PdSreA. In addition, PdSreA protein was predicted to contain two transmembrane domains, but PdSreB contains no transmembrane domains (S2 Fig).

### Disruption of *PdsreA* and *PdsreB* in *P*. *digitatum*

*PdsreA* and *PdsreB* were independently and simultaneously disrupted in *P*. *digitatum* using a homologous recombination strategy (S1A Fig). Utilizing PCR analysis, one *PdsreA* deletion mutant was identified after screening 81 transformants with the primer pairs P15/P16 and P17/P18. one *PdsreB* deletion mutant was identified from 55 transformants with the primer pairs P21/P22 and P23/P24, and one double deletion mutant was identified from 24 transformants using primer pairs P25/ P26 and P27/P28 (S1 Table), designated Δ*PdsreA*, Δ*PdsreB*, and ΔΔ*PdsreAB*, respectively. Transformation of a functional copy of *PdsreA* or *PdsreB* into the corresponding mutant resulted in a complementation strain designated CP*PdsreA* or CP*PdsreB*. Successful disruption was confirmed further by Southern hybridization. The *PdsreA*-specific probe hybridized an expected 5939-bp band in *Xho*I-digested DNA prepared from Δ*PdsreA* due to the insertion of hygromycin resistance cassette within *PdsreA* and a 2150-bp band was detected from that of wild-type. A 5939-bp band and an additional band larger than 2.2 kb were identified in CP*PdsreA* (S1B Fig). The *PdsreB*-specific probe hybridized an expected 2547-bp band from *Eco*RI-digested genomic DNA of Δ*PdsreB* and a 4713-bp band from that of wild-type. The complementation strain CP*PdsreB* had the 2547-bp and an additional band larger than 4.7 kb. An 1860-bp band was detected in ΔΔ*PdsreAB*, because the neomycin resistance cassette (2.2kb) is shorter than the hygromycin resistance cassette (1.1kb) (S1B Fig).

### *PdsreA* and *PdsreB* are required for ergosterol biosynthesis

**Fig 1:**
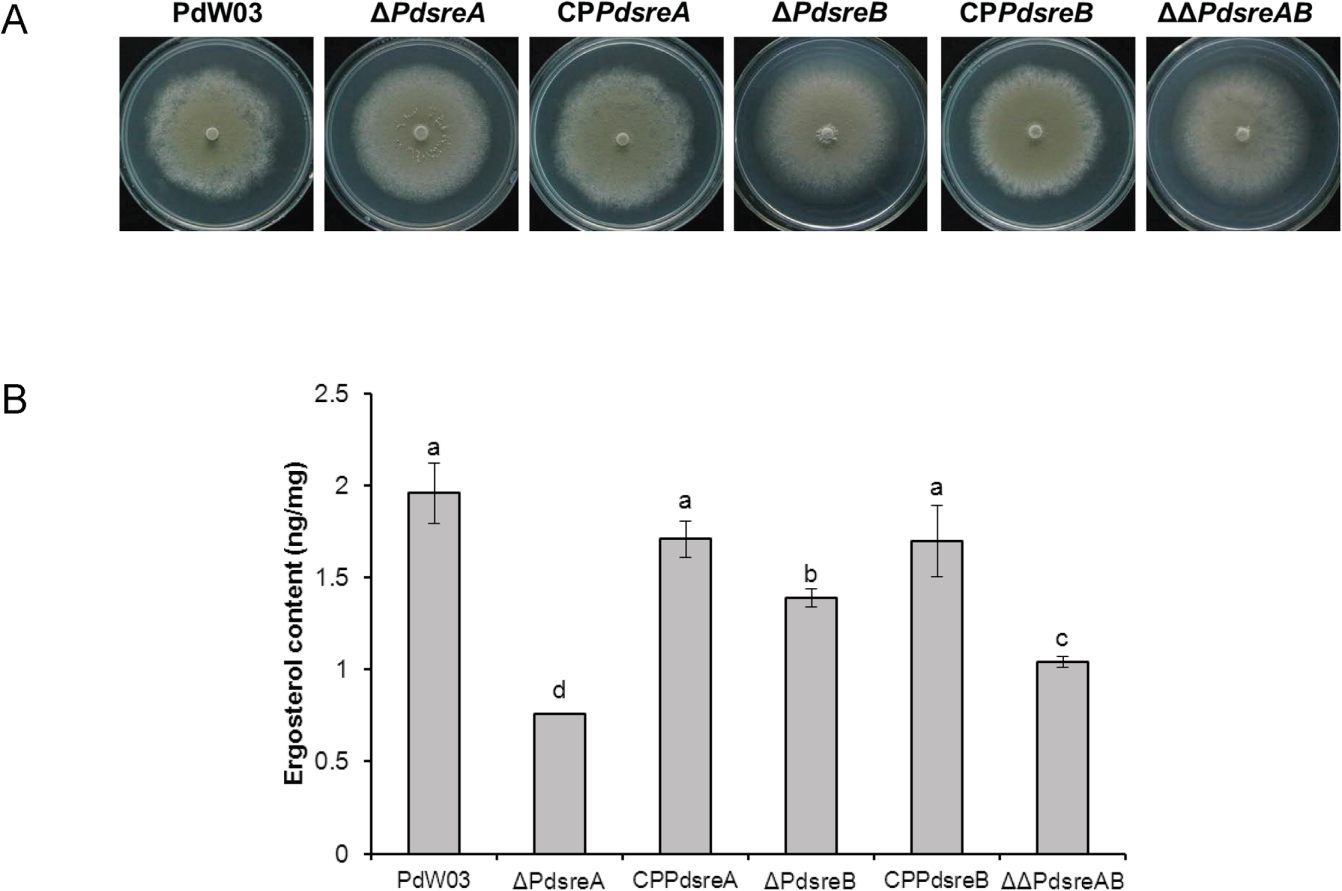
Mycelial growth and ergosterol contents of the *P*. *digitatum* wild-type PdW03, SREBP gene deletion mutants and complementation strains. **(A)** Colony morphology of the wild-type, Δ*PdsreA*, Δ*PdsreB*, ΔΔ*PdsreAB* and complementation strains grown on PDA for 5 days. (B) Ergosterol was extracted and analyzed by HPLC. Different letters on the bars are used to mark statistically significant differences from one another (P<0.05).

The Δ*PdsreA*, Δ*PdsreB*, and ΔΔ*PdsreAB* mutant strains displayed wild-type level of growth on PDA medium (Fig 1A). However, compared to the wild-type, ergosterol production in *PdsreA* mutant was reduced by 61% when grown in YG medium for 48 h (Fig 1B). Inactivation of *PdsreB* alone resulted in fungi that accumulated less ergosterol by approximately 29% in relation to that produced by the wild-type (Fig 1B). Δ*PdsreA* produced a significantly lower amount of ergosterol than Δ*PdsreB*. However, the ergosterol amount produced in ΔΔ*PdsreAB* was reduced by 47%, which was between those in the single deletion mutants (Fig 1B). Introduction and re-expression of a functional copy of *PdsreA* or *PdsreB* into its respective mutant partly restored the levels of ergosterol accumulation (Fig 1B).

### Both *PdsreA* and *PdsreB* contribute to fungicide resistance

**Fig 2:**
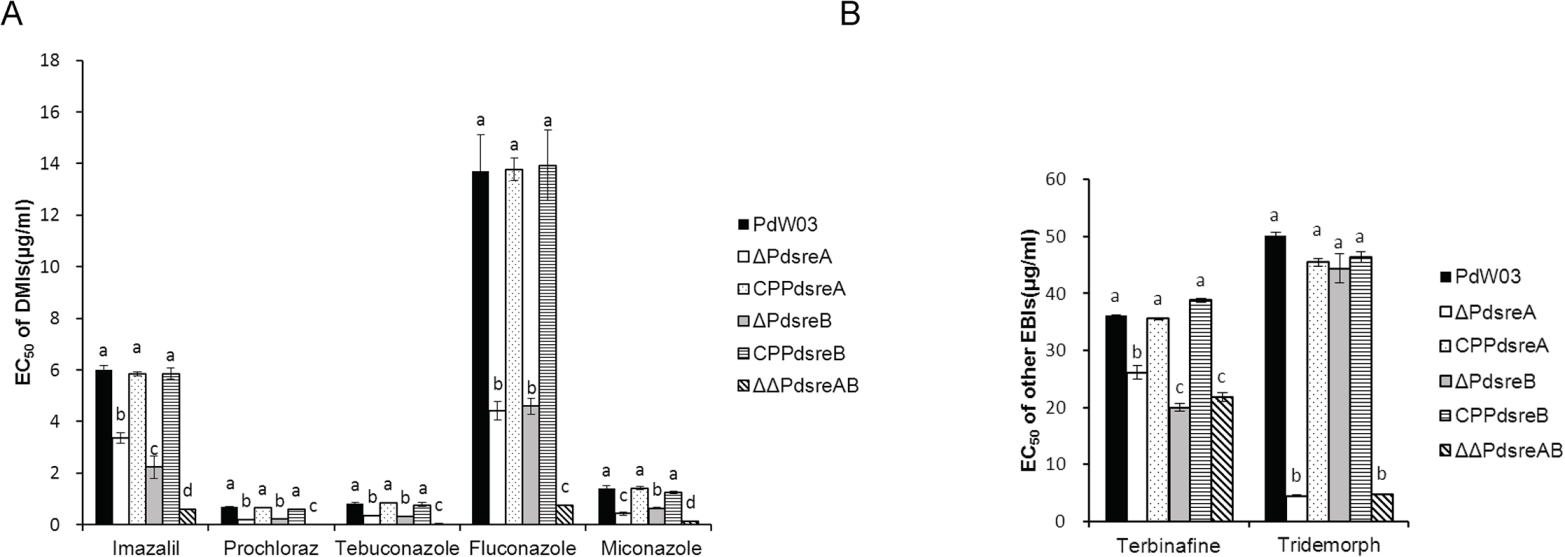
Roles of *PdsreA* and *PdsreB* in fungicide resistance. EC_50_ values of the wild-type PdW03, gene deletion mutants and complementation strains to (A) DMIs and (B) other EBIs. Values on the bars followed by the same letter in each column are not significantly different at P = 0.05.

*P*. *digitatum* strains impaired at *PdsreA*, *PdsreB*, or both increased sensitivity to five DMI fungicides tested. ΔΔ*PdsreAB* double mutant was more sensitive to the test DMIs than the strain mutated at a single gene (Fig 2A). Compared to the wild-type strain, radial growth of Δ*PdsreA* and Δ*PdsreB* reduced by 40~70% on medium containing a DMI fungicide, while that of ΔΔ*PdsreAB* reduced by 90~98%. Δ*PdsreA*, Δ*PdsreB* and ΔΔ*PdsreAB* also increased sensitivity to terbinafine fungicide (Fig 2B), an Erg1 inhibitor [37]. However, strains only defective for *PdsreA* but not *PdsreB* were hypersensitive to tridemorph (Fig 2B), an Erg2 inhibitor [8]. The defect of fungicide resistance in gene deletion mutants was restored by introduction and re-expression of a functional copy of *PdsreA* or *PdsreB* into its respective mutant (Fig 2A and 2B).

### *PdsreA* and *PdsreB* are involved in sensitivity to different chemical stresses

**Fig 3:**
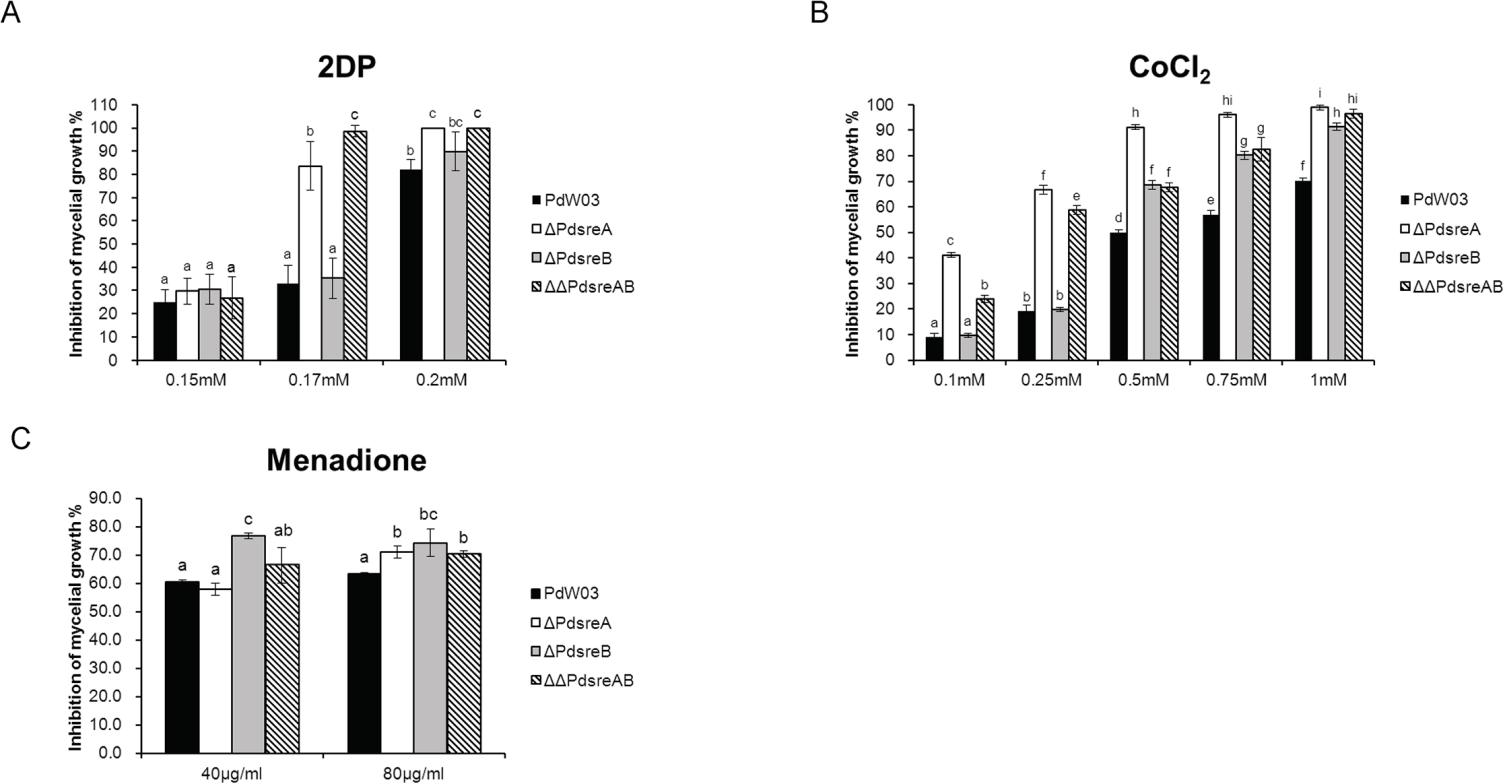
Sensitivity of the wild-type PdW03 and the SREBP mutants to different chemical agents. (A) Inhibition of mycelial growth by iron chelator 2,2-dipyridyl (2DP) at the concentrations indicated in the figure. (B) Fungal growth on PDA medium with serial concentrations of CoCl_2_. (C) Fungal growth on PDA medium amended with menadione. All plates were incubated for 5 days at 25°C. Different letters are used to mark statistically significant differences from one another (P<0.05).

The roles of *PdsreA* and *PdsreB* in adaptation to iron-depleted environment were assessed on medium containing the iron chelator 2DP [38]. The wild-type strain reduced radial growth by ~33% when grown on medium amended with 0.17 mM 2DP (Fig 3A). Both Δ*PdsreA* and ΔΔ*PdsreAB* displayed elevated sensitivity compared to wild-type. When testing at 0.2 mM 2DP, wild-type reduced growth by 87% and growth of both Δ*PdsreA* and ΔΔ*PdsreAB* were completely (100%) suppressed (Fig 3A). However, Δ*PdsreB* displayed wild-type levels of sensitivity to 2DP (Fig 3A).

CoCl_2_ has been widely used as a hypoxia-mimicking agent in many organisms [39]. We tested the sensitivity of PdW03 and SREBP mutants to the gradient concentrations of CoCl_2_. Compared to the wild-type, both Δ*PdsreA* and ΔΔ*PdsreAB* showed dramatic growth reduction as the concentrations of CoCl_2_ increased (Fig 3B). Δ*PdsreB* also increased sensitivity to CoCl_2_, even though the level of sensitivity was less severe than Δ*PdsreA* or ΔΔ*PdsreAB* (Fig 3B).

Menadione is a superoxide-generating agent [40]. Δ*PdsreB*, but not Δ*PdsreA*, exhibited an increased growth inhibition under 40 μg/ml menadione compared to the wild-type (Fig 3C). Under 80 μg/ml menadione, all the three SREBP mutants showed an increased sensitivity compared to the wild-type (Fig 3C). Introduction of *PdsreA* or *PdsreB* into the respective mutants restored chemical sensitivity phenotypes seen in Δ*PdsreA* or Δ*PdsreB* (S3 Fig).

### SREBPs are required for the formation of aerial hyphae on citrus fruit peels

**Fig 4:**
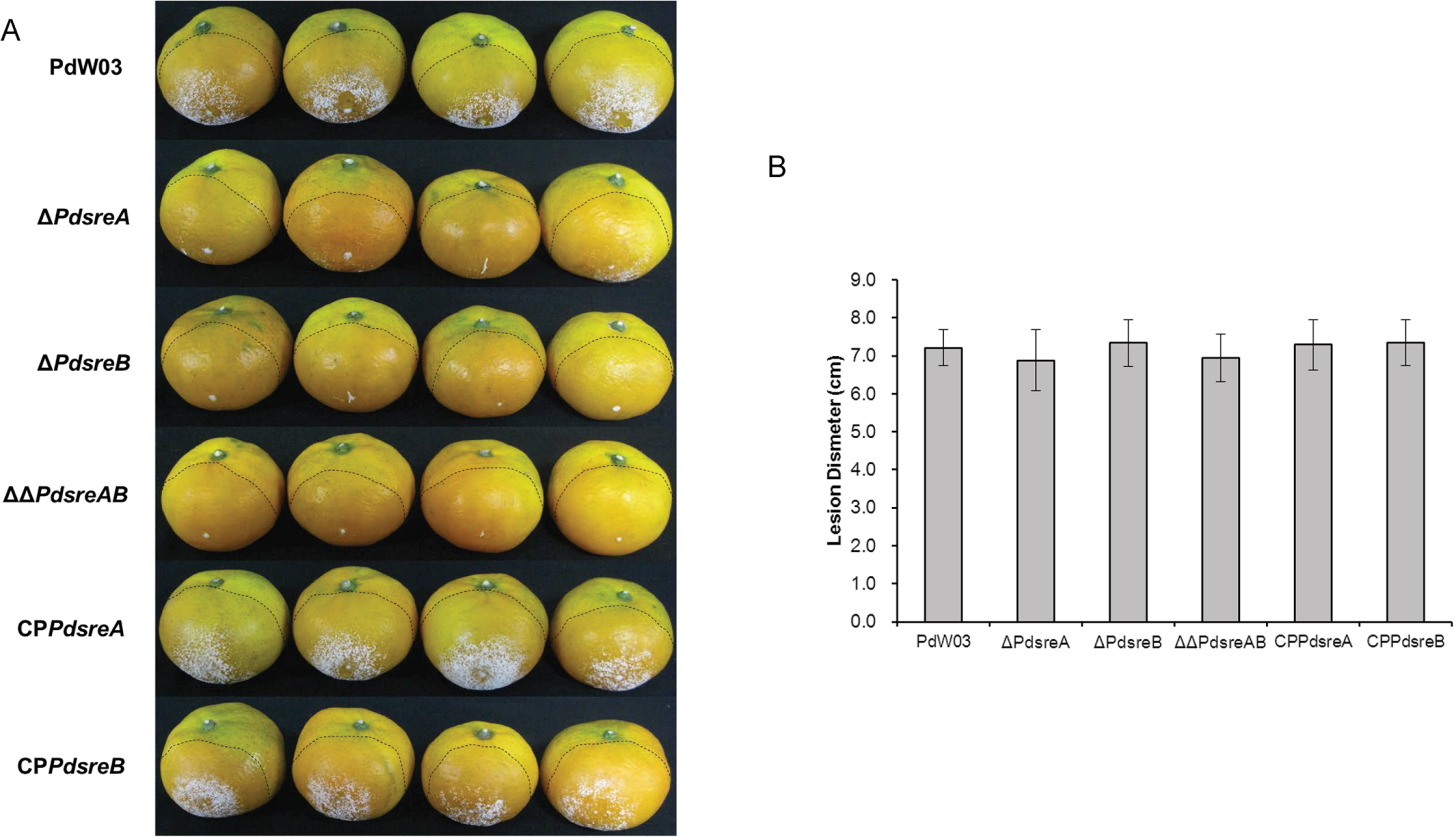
Infection assays of the wild-type PdW03 and SREBP mutants. (A) Decay symptoms in citrus fruits inoculated with the indicated strains, and incubated at room temperature for 4 days. (B) Average diameter of the macerated lesions in citrus fruits. Dashed lines denote the lesion diameter.

Virulence assays on mandarin revealed that *P*. *digitatum* strains lacking *PdsreA*, *PdsreB*, or both induced macerated lesions of the sizes similar to those induced by wild-type (Fig 4A and 4B). Wild-type produced abundant aerial mycelia on affected peel surface 4 days post inoculation (dpi). Unlike the wild-type, both Δ*PdsreB* and ΔΔ*PdsreAB* produced aerial mycelia only at wound sites (Fig 4A). Although most of the fruit inoculated with Δ*PdsreA* developed aerial mycelia, it was observed that mycelia produced by Δ*PdsreA* were much thinner than those produced by the wild-type (Fig 4A).

### Roles of *PdsreA* and *PdsreB* in transcriptional regulation

**Fig 5:**
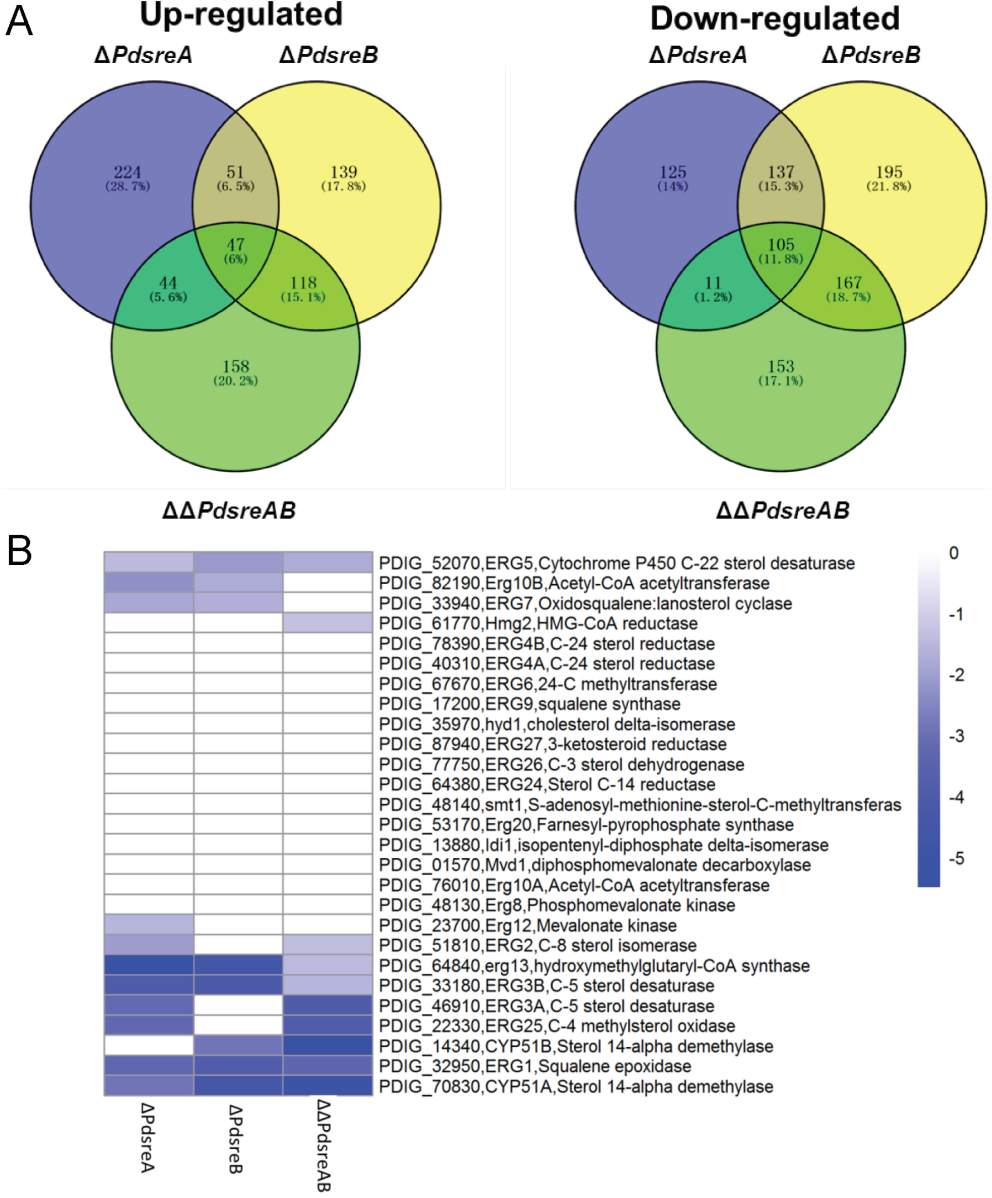
RNA-seq analysis of Δ*PdsreA*, Δ*PdsreB* and ΔΔ*PdsreAB*. (A)Venn diagram showing the up-regulated and down-regulated genes in SREBP mutants compared with wild-type strain. (B) Heat map presentation of gene expressions involved in ergosterol biosynthesis pathway.

To understand the regulatory landscapes of the two SREBPs in *P*. *digitatum*, we compared the whole genome expression profiles of wild-type and three SREBP mutants using RNA-Seq. Using the criteria of false discovery rate set at < 1% and fold change set at >2, substantial changes in the transcriptome between wild-type and SREBP mutants were observed. In total, 744 DEGs were identified in Δ*PdsreA* compared to the wild-type, in which 366 genes were up-regulated and 378 genes were down-regulated (Fig 5A). Of 929 DEGs identified in Δ*PdsreB* compared to the wild-type, 355 genes were up-regulated and 604 genes were down-regulated (Fig 5A). Of 803 DEGs identified in ΔΔ*PdsreAB* compared to the wild-type, the expression levels of 367 genes increased and the expression of other 436 genes decreased (Fig 5A). Of all DEGs identified in three SREBP mutants, 47 genes were commonly up-regulated and 105 genes were commonly down-regulated in all three mutants (Fig 5A and S2 Table). GO analysis revealed that genes showing elevated mRNA levels in Δ*PdsreA* were primarily enriched in transmembrane transport, amino acid transmembrane transport and carbohydrate metabolic processes. Genes displaying reduced transcript levels in Δ*PdsreA* were mainly enriched in the process of stress response (S3 Table). Genes with increased levels in Δ*PdsreB* were enriched in functions involving hydrogen ion and copper ion transmembrane transports, nucleosome assembly, mitochondrial electron transport, ATP hydrolysis coupled proton transport, protein folding, and polysaccharide catabolic process. Genes with decreased levels in Δ*PdsreB* included those involved in DNA integration, transmembrane transport, amino acid transmembrane transport, and response to stress (S3 Table). Inactivation of both *PdsreA* and *PdsreB* increased the expression of genes involved in cellular amino acid biosynthetic process, pyridoxal phosphate biosynthetic process, lysine biosynthetic process via aminoadipic acid and translation. It was observed that the expression of genes associated with DNA integration, fatty acid biosynthetic process and microtubule-based movement was apparently down-regulated in ΔΔ*PdsreAB* (S3 Table).

**Fig 6:**
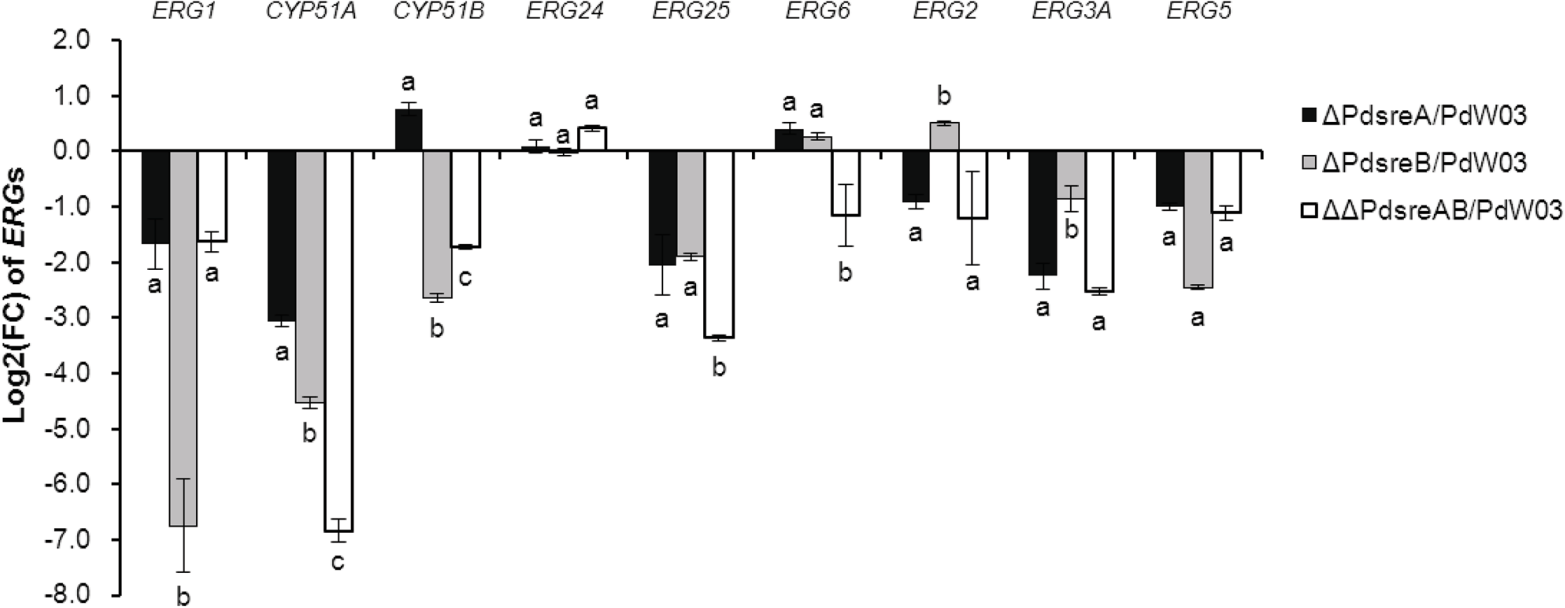
qRT-PCR confirmation of ergosterol biosynthesis genes. Expression levels of ergosterol biosynthesis genes were examined in Δ*PdsreA*, Δ*PdsreB* and wild-type PdW03 strains. Log2 of fold change (FC) expression levels were normalized to actin expression levels in each sample and data is presented relative to wild-type expression levels using 2^-ΔΔCt^ method. Values on the bars followed by the same letter in each column are not significantly different at P = 0.05.

DEGs involved in ergosterol biosynthesis were all down-regulated in the three SREBP mutants (Fig 5B). Transcript levels of the genes encoding Erg5 (C-22 sterol desaturase), Erg10B (acetoacetyl-CoA acetyltransferase), Erg7 (lanosterol synthase), Erg13 (HMG-CoA synthase), Erg3B (C-5 sterol desaturase), Erg1 (squaleneepoxidase) and Cyp51A*/*Erg11A (lanosterol 14-α-demythylase) had 2-fold or greater reductions in both Δ*PdsreA* and Δ*PdsreB* mutants. The genes encoding Erg12 (mevalonate kinase), Erg2 (C-8 sterol isomerase), Erg3A (C-5 sterol desaturase) and Erg25 (C-4 methyl sterol desaturase) were down-regulated in Δ*PdsreA* but not in Δ*PdsreB*. Reduced expression of the gene encoding Cyp51B/Erg11B (lanosterol 14-α-demythylase) was only detected in Δ*PdsreB*. qRT-PCR analysis revealed that genes encoding Erg1, Cyp51A, Erg25, Erg3A, and Erg5 were down-regulated in the strain lacking *PdsreA* or *PdsreB* and genes encoding Cyp51B and Erg2 were differentially expressed in Δ*PdsreA* and Δ*PdsreB*, confirming the validation of the DEG data (Fig 6). In addition, we analyzed the 2-kb sequences upstream of the putative ATG translational start codon of genes involved in the ergosterol biosynthesis pathway using the 11-bp SrbA-binding motif identified in *A*. *fumigatus* [27]. The results showed that the *A*. *fumigatus* SrbA binding sites were found in the promoter regions of the genes encoding Erg10A, Hmg2, Erg12, Idi1, Erg1, Cyp51A, Cyp51B, Erg25, Erg27, Erg3B, and Erg4B (S4 Table). We also found putative SRE motif in the promoter regions of *PdsreA* and *PdsreB* (S4 Table).

Further examination of gene expression profiles identified genes that are commonly or differentially regulated by *PdsreA* and *PdsreB* in *P*. *digitatum*. In total, 340 genes were commonly regulated by both *PdsreA* and *PdsreB* (S4A Fig). Analysis revealed that 392 genes were uniquely regulated by *PdsreA* while 607 ones were exclusively regulated by *PdsreB* (S4A Fig). Identification of conserved protein domains among all DEGs were performed by searching against the Pfam database. Detailed annotation of those DEGs is listed in S5–S7 Tables. Analysis of the proteins conceptually translated from the genes commonly regulated by *PdsreA* and *PdsreB*, with the highest frequency set greater than 5, revealed that many proteins contain domains were associated with major facilitator superfamily (MFS), cytochrome P450, GMC oxidoreductase, integrase core domain, amino acid permease, reverse transcriptase, and fungal Zn(2)-Cys(6) binuclear cluster domain (S4B Fig). The proteins whose gene expression was primarily regulated by *Pdsre*A contain domains were associated with MFS, sugar transporter, amino acid permease and enoyl reductase (S4C Fig). The genes uniquely regulated by *PdsreB* encode proteins containing hAT family C-terminal dimerisation region, MFS, fungal specific transcription factor domain, reverse transcriptase, phosphopantetheine attachment site, integrase core domain, protein kinase domain, RNA recognition motif, AMP-binding enzyme, beta-ketoacyl synthase, ubiquitin-conjugating enzyme, condensation domain and acyl transferase domain (S4D Fig).

## Discussion

Sterols are essential components of cell membranes [22]. The SREBP transcription factors, containing a bHLH domain, act as key regulators of sterol homestasis, and universally distribute in fungi [25]. In *P*. *digitatum*, one SREBP homolog was identified and partially characterized previously [21]. In the current study, using a systemic analysis, we identified another SREBP homolog in *P*. *digitatum*, named *PdsreB*, and compared its protein structure and regulatory roles with *PdsreA*. PdSreA contains a bHLH domain at the N terminus and two transmembrane domains. PdSreB contains a bHLH domain at the C terminus but has no transmembrane domain. Functional analysis revealed further that both *PdsreA* and *PdsreB* play global regulatory roles in a wide array of biological functions, particularly in relation to ergosterol biosynthesis and fungicide resistance. The divergent functions of the SREBP transcription regulators were also presented and discussed.

SREBPs have been well documented for their regulatory roles in sterol biosynthesis in mammals [22]. The SREBP ortholog in fungal kingdom, called Sre1, was first identified in the fission yeast *S*. *pombe* [41]. So far, orthologs of SREBP have been identified as key regulators of ergosterol biosynthesis in several human pathogenic fungi, such as *C*. *neoformans*, *A*. *fumigatus* and *Histoplasma capsulatum*, as well as the plant pathogenic fungi, such as *M*. *oryzae* and *P*. *digitatum* [21,26,42,43,44]. In this study, we demonstrated that deletion of *PdsreA*, *PdsreB*, or both, decreased ergosterol content considerably, suggesting the conserved regulation of ergosterol biosynthesis of SREBPs in *P*. *digitatum* (Fig 1B). This phenotype was supported by the transcriptome data and qRT-PCR in which the expression levels of genes involved in ergosterol biosynthesis were significantly decreased in a fungal strain defective for *PdsreA* or *PdsreB* (Figs 5B and 6). Moreover, searching for *A*. *fumigatus* SrbA-binding motif revealed that the bHLH transcription factors associated with ergosterol biosynthesis enzymes often interact physically with each other (S4 Table). However, ergosterol content in double deletion mutant is higher than that in *PdsreA* single deletion mutant (Fig 1B), indicating the existence of other transcription factors mediating ergosterol biosynthesis that might be activated when both SREBPs were blocked.

When tested on medium amended with DMIs or EBIs, growth of Δ*PdsreA*, Δ*PdsreB* and ΔΔ*PdsreAB* was significantly decreased compared with that of wild-type, indicating that SREBPs are required for fungicide resistance (Fig 2A and 2B). Although *PdsreA* and *PdsreB* play indistinguishable roles coping with *CYP51*-targeted DMIs, they appear to have divergent functions in response to other EBIs. Fungal strains impaired at *PdsreA*, *PdsreB*, or both display increased sensitivity to terbinafine, an Erg1 inhibitor [37]. However, only the mutant strain lacking *PdsreA* increased sensitivity to tridemorph, an Erg2 inhibitor [4]. The results are supported further by the fact that expression of *ERG1* is regulated by both *PdsreA* and *PdsreB*, while expression of *ERG2* is primarily regulated by *PdsreA* (Fig 6).

Iron is an essential nutrient and plays a major role in many enzymatic reactions [45]. Experiments have demonstrated that SREBP homologs are important regulators for iron metabolism in *C*. *neoformans* and *A*. *fumigatus*. Deletion of *SRE1* in *C*. *neoformans* and of *srbA* in *A*. *fumigatus* resulted in fungal strains that were impaired for growth on iron-limiting minimal medium [42,46]. In *P*. *digitatum*, we found that fungal strains defective at *PdsreA* but not *PdsreB*, were more sensitive to 2DP (Fig 3A). In our transcriptome data, genes encoding proteins that are related to iron homostasis were differentially affected in Δ*PdsreA* and Δ*PdsreB*. For example: a decreased expression of genes encoding a nonribosomal siderophore peptide synthase (sidC) and a ferrooxidoreductase (Fet3) were detected in Δ*PdsreA* or Δ*PdsreB*. Loss of *PdsreA*, but not *PdsreB*, decreased the expression of genes encoding a siderochrome-iron transporter (Sit1), a high-affinity iron transporter (FtrA) and a L-ornithine N5-oxygenase (SidA) while loss of *PdsreB*, but not *PdsreA*, reduced the mRNA levels of genes encoding a transcription factor (HapB) and a coproporphyrinogen III oxidase (Hem13). These data indicates the differentiated functions of *PdsreA* and *PdsreB* in iron homostasis.

When testing for sensitivity to oxidative stress, Δ*PdsreA*, Δ*PdsreB*, and ΔΔ*PdsreAB* display moderate sensitivity only to menadione (Fig 3B), a compound that has been demonstrated to generate superoxide [47]. The *P*. *digitatum* SREBP mutants display wild-type resistance to diethyl maleate (3 mM), H_2_O_2_ (200 mM), and NaNO_2_ (20 mM) (data not shown), indicating that SREBPs play no roles in other oxidative stress resistance. Similar results were also found in *A*. *fumigatus* in which the *srbA*-deleted mutant displayed wild-type sensitivity to H_2_O_2_ [43]. In contrast, the *C*. *neoformans* strain impaired at *SRE1* increased sensitivity to reactive oxygen species (ROS)- and reactive nitrogen species (RNS)-generating compounds [40]. The *M*. *oryzae* mutant impaired at *MoSRE1* also increased sensitivity to H_2_O_2_ [26].

In our study, we found that deleting *PdsreA*, *PdsreB* or both resulted in no significant changes of macerated lesions in inoculated citrus fruit (Fig 4A and 4B), inconsistent with the recent finding that deleting the *PdsreA* reduced lesion formation by ~44% [21]. This discrepancy could be due to different citrus cultivars used for virulence assays and different *P*. *digitatum* wild-type strains for mutagenesis strategy. Although the size of maceration lesions induced by the wild-type and the SREBP mutants are similar, mycelial growth of the Δ*PdsreA* on the surface of affected citrus fruits was obviously retarded compared with that of the wild-type strains, while the Δ*PdsreB* and ΔΔ*PdsreAB* strains apparently failed to produce aerial mycelia on affected fruits (Fig 4A). Similar phenotype has been characterized previously that the *P*. *digitatum* strain defective of a mitogen-activated protein (MAP) kinase B coding gene failed to break through the stratum epidermis of citrus fruit after inoculation, even though the mycelia could proliferate below fruit peels [48]. In *M*. *oryzae*, invasive growth of *MoSRE1*-impaired mutant within rice sheath cells was much delayed compared to that of wild-type strain, but the *MoSRE1* mutant displayed wild-type disease severity [26].

It appears that SREBP homologs play different regulatory roles in virulence in plant and human pathogenic fungi. For human pathogenic fungi, the ability to survive in hypoxic condition is critical for their virulence as demonstrated in *A*. *fumigatus*, *C*. *neoformans* and *H*. *capsulatum* [42,43,44]. Transcriptome analysis of the *A*. *fumigatus* SREBP mutants under hypoxic conditions has revealed the involvement of ergosterol biosynthesis and iron acquisition in hypoxic adaptation [46]. Although *PdsreA* and *PdsreB* are associated with sensitivity to the hypoxia-mimetic agent CoCl_2_ (Fig 3B), deletion of *PdsreA* or *PdsreB* does not decrease the disease severity on citrus fruits. This phenomenon was also observed for *MoSRE1* in *M*. *orzyae* [26], suggesting the divergence on pathogenicity between plant and human pathogenic fungi.

Both *PdsreA* and *PdsreB* were also shown to play important roles in global gene regulation in *P*. *digitatum* in our transcriptome data. Transcriptome analysis has identified many genes that are commonly regulated by both *PdsreA* and *PdsreB* (S4A Fig). These genes are primarily involved in metabolic process, oxidation-reduction process, transmembrane transport, iron ion binding and lyase activity, indicating that *PdsreA* and *PdsreB* share redundant functions in these aspects (S5 Table). Differences in gene regulation between *PdsreA* and *PdsreB* were evidenced by the distinct sets of DEGs (S6 and S7 Tables), indicating that *PdsreA* and *PdsreB* also play unique regulatory roles. Transcriptome analysis revealed that *PdsreB* appears to regulate more genes than *PdsreA*. In total, 607 genes were found to be uniquely regulated by *PdsreB* while 392 genes were uniquely regulated by *PdsreA* (S6 and S7 Tables). The domain categories uniquely regulated by *PdsreB* (463) are also greater than those (342) regulated by *PdsreA* (S6 and S7 Tables), indicating a more extensive regulating range of *PdsreB*. In addition, many MFS-coding genes were over-represented in all DEGs, suggesting a profound role of SREBPs on the regulation of the MFS functions in *P*. *digitatum*. This might support the roles that *PdsreA* and *PdsreB* play in fungicide resistance, as MFS transporters have been demonstrated to be related to multidrug resistance in fungi [49].

In *A*. *fumigatus*, evidence has been provided to demonstrate that expression of *srbA* and *srbB* was reciprocally regulated [27]. Experiment has shown that *A*. *fumigatus* SrbA could bind to its own promoter and the promoter of *srbB*, and vice versa. Unlike the *srbA* and *srbB* of *A*. *fumigatus*, expression of *PdsreA* is not affected by mutation of *PdsreB*, and vice versa (S3 Table). The results further indicate that, apart from biological functions, the mechanisms underlying the regulation of the two SREBP transcription factors could be different among different organisms. However, the promoter regions of both *PdsreA* and *PdsreB* contain putative SRE sequences (S4 Table). It is possible that PdSreA and PdSreB interact to each other at post-translational levels but not transcriptional levels. This speculation awaits further investigation.

In conclusion, in addition to previously identified PdSreA, the present study identified another SREBP transcription factor PdSreB, which co-regulates ergosterol biosynthesis and DMI resistance with PdSreA in *P*. *digitatum*. Both PdSreA and PdSreB are different in protein structures. Functional analysis of *PdsreA* and *PdsreB* demonstrates the divergent regulatory roles on adaptation to iron starvation and hypoxia-mimicking condition. Moreover, transcriptome data extend the insight for further investigation into other novel functions of *PdsreB*.

## Supporting information

S1 Fig Construction and identification of Δ*PdsreA*, Δ*PdsreB* and ΔΔ*PdsreAB*. (A) The homologous gene recombination strategy. (B) Southern blot analysis. The size of DNA standards is indicated on the left of the blot. (C) qRT-PCR analysis of gene deletion mutants and complementation strains.
(PDF)

S2 Fig Evolution of SREBPs in fungi. Phylogenic tree was constructed by concatenating 27 conserved ribosome proteins using MEGA6. The number and gene structure of SREBPs in fungi were annotated. Domain information was obtained from the pfam database.
(PDF)

S3 Fig Sensitivity of the wild-type PdW03 and the complementation strains to 2DP, CoCl_2_ and menadione. (PDF)

S4 Fig The common and discrepant regulation of *PdsreA* and *PdsreB* on gene expression in *P*. *digitatum*. (A) Venn diagram showing the common and discrepant regulated genes in Δ*PdsreA* and Δ*PdsreB* mutants. (B) The top 8 protein domains with the highest frequency (>5) in the proteins commonly regulated by *PdsreA* and *PdsreB*. (C) The top 4 protein domains with the highest frequency (>5) in the *PdsreA* uniquely regulated proteins. (D) The top 15 protein domains with the highest frequency (>5) in the *PdsreB*-uniquely- regulated proteins. The number of proteins included in the respective family is indicated.
(PDF)

S1 Table Primers used in this study. (PDF)

S2 Table Annotation of genes commonly up-regulated or down-regulated in all three mutants. (PDF)

S3 Table GO enrichment of differentially expressed genes. (PDF)

S4 Table Putative SREs found in the promoter regions of genes involved in ergosterol biosynthesis, *PdsreA* and *PdsreB*. (PDF)

S5 Table Annotation of genes commonly regulated by *PdsreA* and *PdsreB*. (PDF)

S6 Table Annotation of genes uniquely regulated by *PdsreA*. (PDF)

S7 Table Annotation of genes uniquely regulated by *PdsreB*. (PDF)
