# Supplementary material for: Functional analysis of two sterol regulatory element binding proteins in Penicillium digitatum
Source: PLoS One. 2017 May 3;12(5):e0176485. doi: 10.1371/journal.pone.0176485 (PMC5415137; doi:10.1371/journal.pone.0176485)
Supplement: S1 Fig — (A) The homologous gene recombination strategy. (B) Southern blot analysis. The size of DNA standards is indicated on the left of the blot. (C) qRT-PCR analysis of gene deletion mutants and complementation strains. (PDF) [file pone.0176485.s001.pdf]

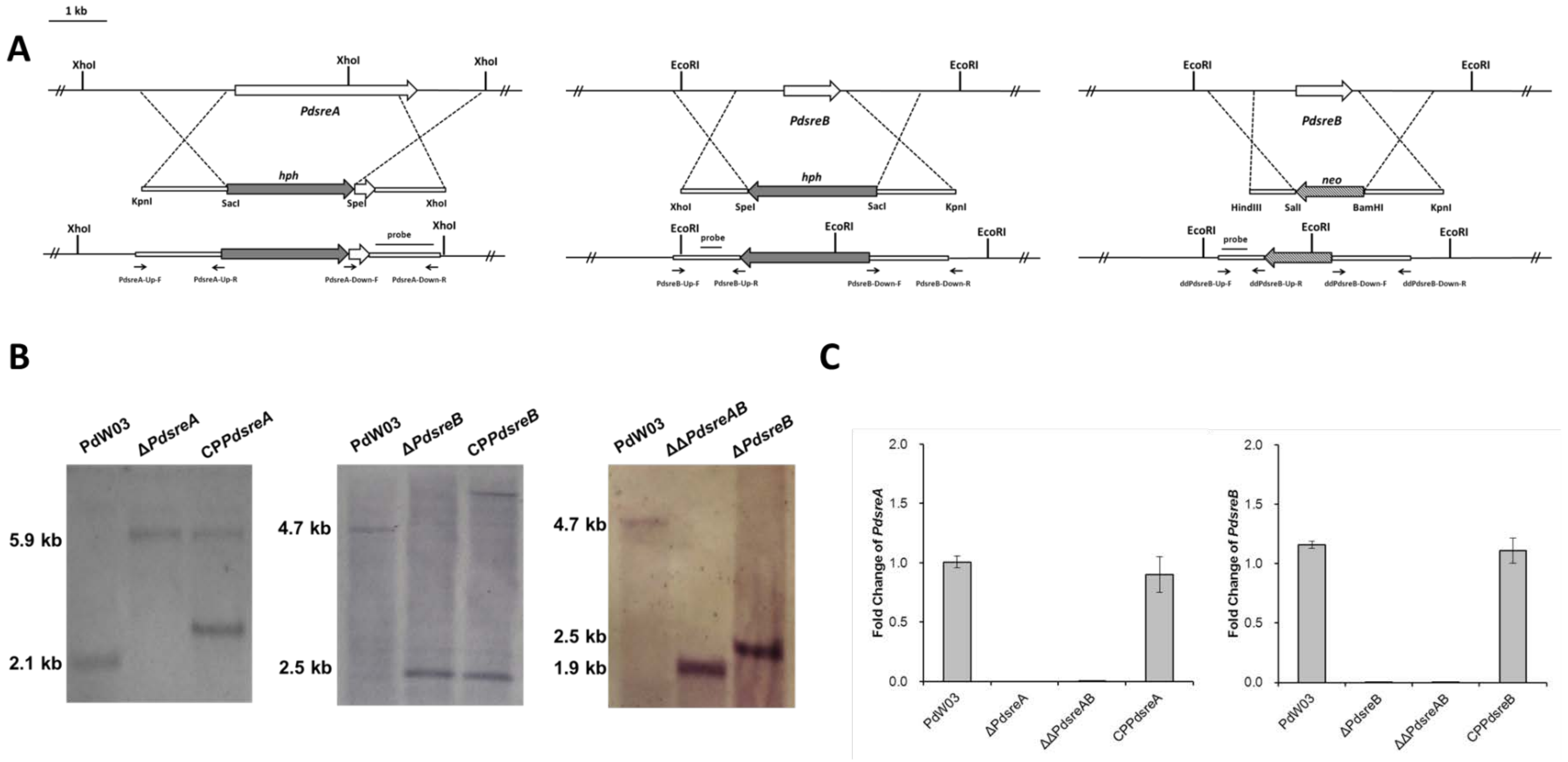

S1 Fig. Construction and identification of Δ*PdsreA*, Δ*PdsreB* and ΔΔ*PdsreAB*. (A) The homologous gene recombination strategy. (B) Southern blot analysis. The size of DNA standards is indicated on the left of the blot. (C) qRT-PCR analysis of gene deletion mutants and complementation strains.
