# Supplementary material for: Functional analysis of two sterol regulatory element binding proteins in Penicillium digitatum
Source: PLoS One. 2017 May 3;12(5):e0176485. doi: 10.1371/journal.pone.0176485 (PMC5415137; doi:10.1371/journal.pone.0176485)
Supplement: S2 Fig — Phylogenic tree was constructed by concatenating 27 conserved ribosome proteins using MEGA6. The number and gene structure of SREBPs in fungi were annotated. Domain information was obtained from the pfam database. (PDF) [file pone.0176485.s002.pdf]

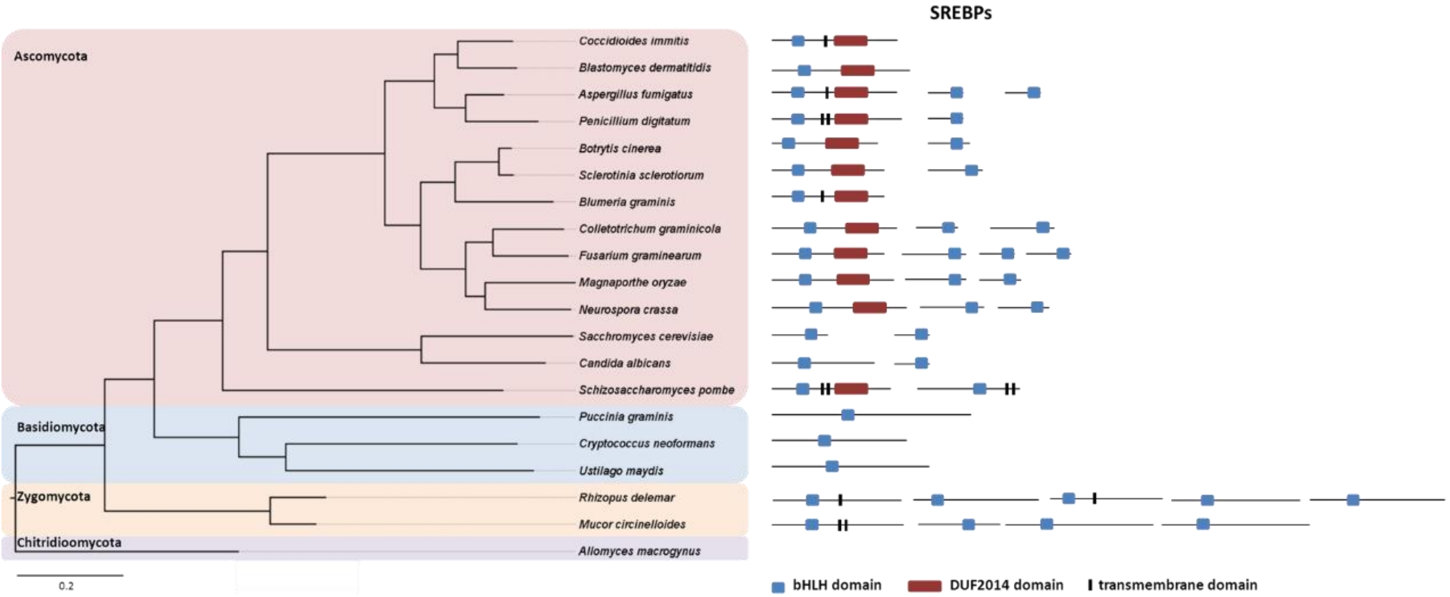

S2 Fig. Evolution of SREBPs in fungi. Phylogenic tree was constructed by concatenating 27 conserved ribosome proteins using MEGA6. The number and gene structure of SREBPs in fungi were annotated. Domain information was obtained from the pfam database.
