# Supplementary material for: Functional analysis of two sterol regulatory element binding proteins in Penicillium digitatum
Source: PLoS One. 2017 May 3;12(5):e0176485. doi: 10.1371/journal.pone.0176485 (PMC5415137; doi:10.1371/journal.pone.0176485)
Supplement: S3 Fig — (PDF) [file pone.0176485.s003.pdf]

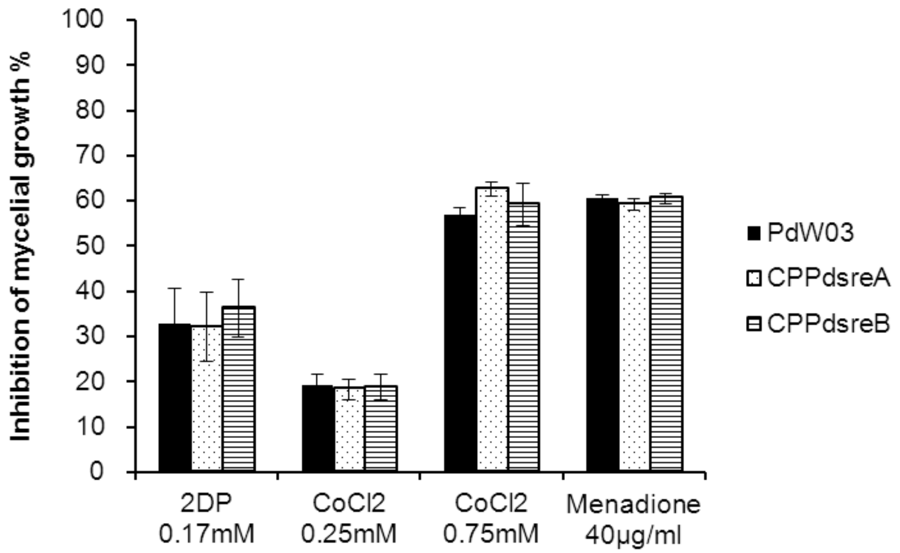

S3 Fig. Sensitivity of the wild-type PdW03 and the complementation strains to 2DP, $CoCl_2$ and menadione.
