# Supplementary material for: Functional analysis of two sterol regulatory element binding proteins in Penicillium digitatum
Source: PLoS One. 2017 May 3;12(5):e0176485. doi: 10.1371/journal.pone.0176485 (PMC5415137; doi:10.1371/journal.pone.0176485)
Supplement: S4 Fig — (A) Venn diagram showing the common and discrepant regulated genes in ΔPdsreA and ΔPdsreB mutants. (B) The top 8 protein domains with the highest frequency (>5) in the proteins commonly regulated by PdsreA and PdsreB. (C) The top 4 protein domains with the highest frequency (>5) in the PdsreA uniquely regulated proteins. (D) The top 15 protein domains with the highest frequency (>5) in the PdsreB-uniquely- regulated proteins. The number of proteins included in the respective family is indicated. (PDF) [file pone.0176485.s004.pdf]

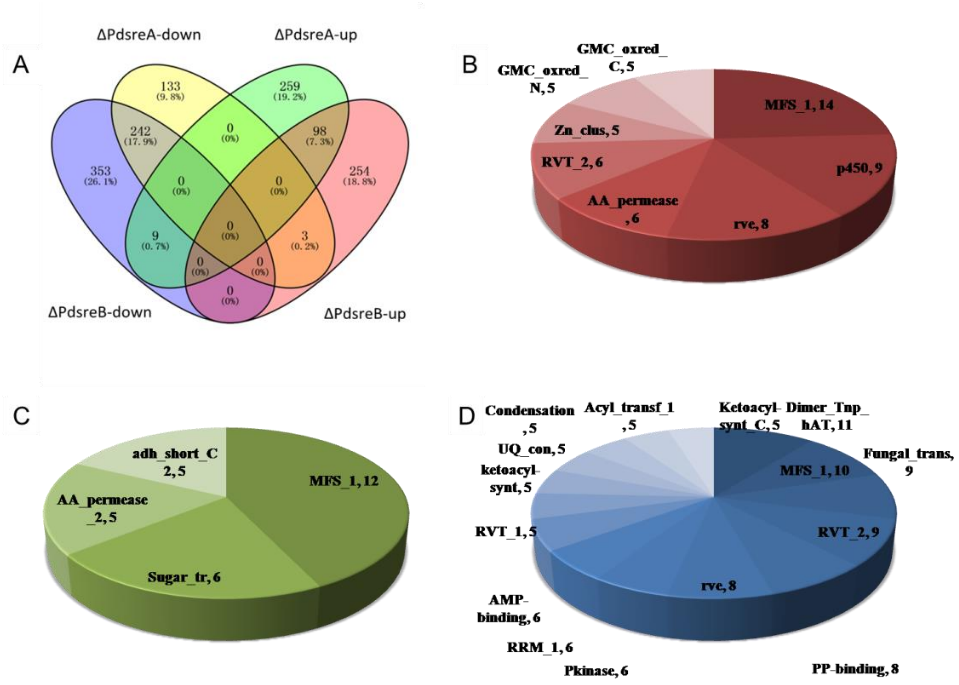

S4 Fig. The common and discrepant regulation of *PdsreA* and *PdsreB* on gene expression in *P. digitatum*. (A) Venn diagram showing the common and discrepant regulated genes in Δ*PdsreA* and Δ*PdsreB* mutants. (B) The top 8 protein domains with the highest frequency (>5) in the proteins commonly regulated by *PdsreA* and *PdsreB*. (C) The top 4 protein domains with the highest frequency (>5) in the *PdsreA* uniquely regulated proteins. (D) The top 15 protein domains with the highest frequency (>5) in the *PdsreB*-uniquely- regulated proteins. The number of proteins included in the respective family is indicated.
