# Supplementary material for: Functional analysis of two sterol regulatory element binding proteins in Penicillium digitatum
Source: PLoS One. 2017 May 3;12(5):e0176485. doi: 10.1371/journal.pone.0176485 (PMC5415137; doi:10.1371/journal.pone.0176485)
Supplement: S1 Table — (PDF) [file pone.0176485.s005.pdf]

S1 Table. Primers used in this study

Primer code	Primer	Sequence (5'-3')[a]	Relevant characteristics
P1	PdsreA_Up-F	Gactagt CGCCCGAAATGCCTACACC	PCR primers to amplify *PdsreA* upstream fragment for the construction of *PdsreA* deletion plasmid
P2	PdsreA_Up-R	CCGctcgagCGGTGACTACAGGCCAGGAGA	
P3	PdsreA_Down-F	GGggtaccCCCTTTGTCGCGAGTTCTTA	PCR primers to amplify *PdsreA* downstream fragment for the construction of *PdsreA* deletion plasmid
P4	PdsreA_Down-R	CgagctcGTGGATGAAGCTCAACAAG	
P5	PdsreB_Up-F:	CCGctcgagAATGCGGAAGTGTTGACC	PCR primers to amplify *PdsreB* upstream fragment for the construction of *PdsreB* deletion plasmid
P6	PdsreB_Up-R	GactagtAGCGGCGATTGACAGATA	
P7	PdsreB_Down-F	CgagctcTTTGTGGGTGGGAGGAGG	PCR primers to amplify *PdsreB* downstream fragment for the construction of *PdsreB* deletion plasmid
P8	PdsreB_Down-R	GGggtaccGGTATGGCGGGATAGTCA	
P9	ddPdsreB_Up-F	CCCaagcttGGCAGAACTTGACAGCAT	PCR primers to amplify *PdsreB* upstream fragment for the construction of double deletion plasmid
P10	ddPdsreB_Up-R	ACGCgtcgacGGTGAAGCCAATCCCATC	
P11	ddPdsreB_Down-F	CGggatccTTTGTGGGTGGGAGGAGG	PCR primers to amplify *PdsreB* downstream fragment for the construction of double deletion plasmid
P12	ddPdsreB_Down-R	GGggtaccCGTTTGCTGGCAGTCGTG	
P13	PdSreA_in-F	GAAGCGAAAGACGAGCAG	
P14	PdSreA_in-R	GCAAGGGCACCTAGCATAT	
P15	PdsreA_uphphup-F	GTCCTTGCAGCACTAAATT	PCR primers for identification of *PdsreA* deletion transformants
P16	PdsreA_uphphup-R	TCGCCAACATCTTCTTCT	
P17	PdsreA_hphDD-F	ACCCGCTCGTCTGGCTAA	
P18	PdsreA_hphDD-R	CGCTCGGTTGAGTGGTGA	
P19	PdsreB_in-F	CTTCAGTGACCCATTTGC	
P20	PdsreB_in-R	TCTCCTGCTGGATACCAT	PCR primers for identification of *PdsreB* deletion transformants
P21	PdsreB_uphphup-F	GGGCTTTACTTTCTTCCA	

P22	PdsreB_uphphup-R	TTTCGGGTTTACCTCTTC	
P23	PdsreB_hphDD-F	AACCCGCTCGTCTGGCTAA	
P24	PdsreB_hphDD-R	TCCCTCCAAACTTGAGTAGAAC	
P25	ddPdsreB_upNeoup-F	TAGTGCCACGCCTCAACC	
P26	ddPdsreB_upNeoup-R	TGCCGAGAAAGTATCCATCA	PCR primers for identification of double deletion
P27	ddPdsreB_NeoDD-F	TATCACGGGTAGCCAACG	transformants
P28	ddPdsreB_NeoDD-R	TCCCTCCAAACTTGAGTAGAAC	
P29	CPSreA-F	CCaagcttGGCGCCAAGACATTGTGCGACAA	PCR primers to amplify full-length of PdsreA for the
P30	CPSreA-R	GGggtaccCCTGGCTCTACAACCTAGAGATC	construction of PdsreA complelementation plasmid
P31	CPSreB-F	CCCaagcttAGGGTGGATGACAGGTGA	PCR primers to amplify full-length of PdsreB for the
P32	CPSreB-R	CgagctcCTGCCCGCTGTAGTTAGT	construction of PdsreB complelementation plasmid
P33	qCYP51A-F	CGCTTCTCGAGCCAAATACG	PCR primers for amplification of *CYP51A* gene for the
P34	qCYP51A-R	CAGGCCGTATTTGATGAACTTCTT	expression levels analysis
P35	qCYP51B-F	CACCCAAAGTCGTGCAAAGTAT	PCR primers for amplification of *CYP51B* gene for the
P36	qCYP51B-R	TTGACAAACTTCTTCTGCTCCA	expression levels analysis
P37	q2-actin-R	CCGCCAGACTCAAGACCAAGAAC	PCR primers for amplification of *actin* gene for the
P38	q2-actin-F	TCCACTACTGCCGAGCGTGAAAT	expression levels *analysis*
P39	q-ERG1-F	CAATATCCTCGCACAGGC	PCR primers for amplification of *ERG1* gene for the
P40	q-ERG1-R	CAGATGAGAACCCAAATAGAAA	expression levels analysis
P41	q- ERG6-F	AGGCAGCAGTGGATGAGT	PCR primers for amplification of *ERG6* gene for the
P42	q- ERG6-R	AAATGGAAGGAGGAACCC	expression levels analysis
P43	q- ERG24-F	CTATGTCGTGGGCTTACTG	PCR primers for amplification of *ERG24* gene for the
P44	q- ERG24-R	CTTCTTCTCGCACTTCTCC	expression levels analysis
P45	q-ERG2-F	TTTCTTCTCCGCCGTCTA	PCR primers for amplification of *ERG2* gene for the
P46	q-ERG2-R	TTGAATACCCACTCCTCCTG	expression levels analysis

P47	q- ERG5-F	ACCTGAAAGTCCGCAACG	PCR primers for amplification of *ERG5* gene for the
P48	q- ERG5-R	GGTAAGCCTCCTCATCGTG	expression levels analysis
P49	q-ERG25-F	GCCTGGTATGCTTGGATG	PCR primers for amplification of *ERG25* gene for the
P50	q-ERG25-R	AGCGATGGGACCTTGTTA	expression levels analysis
P51	q-ERG3-F	TTCCTGATTACTTGGCTGTT	PCR primers for amplification of *ERG3* gene for the
P52	q-ERG3-R	GGCGGTGTCGTAGAGTTT	expression levels analysis
P53	qPdsreA-F	CACCGAACGATCCACAGG	PCR primers for amplification of *PdsreA* gene for the
P54	qPdsreA-R	TGATAGACAGACGAGAACCC	expression levels analysis
P55	qPdSreB-F	CTGGGATTAGTGGCTTTT	PCR primers for amplification of *PdsreB* gene for the
P56	qPdSreB-R	GTTTGGTCGTCACTTTCG	expression levels analysis
P57	qPderg4A-F	CAGGAGATATCAAGGGTG	PCR primers for amplification of *ERG4A* gene for the
P58	qPderg4A-R	TGGTAATAGCTGGATCATAC	expression levels analysis
P59	qPderg4B-F	GAGACGGGCGATGTGATT	PCR primers for amplification of *ERG4B* gene for the
P60	qPderg4B-R	CGGCGAAGAAGACAGGAT	expression levels analysis
P61	qPderg7-F	GCAGGAGTCGATTGAGGG	PCR primers for amplification of *ERG7 gene for the*
P62	qPderg7-R	TCTGTATTGCCGAACTTCTTA	*expression levels analysis*
P63	qPderg8-F	AATAATAACCGCAAGGAAGC	PCR primers for amplification of *ERG8* gene for the
P64	qPderg8-R	GCGTGTTCGCTGAATAAGG	expression levels analysis
P65	qPderg9-F	CTGCGGACAGATCGAGAA	PCR primers for amplification of *ERG9* gene for the
P66	qPderg9-R	ACAACGCCCATAAGAGCC	expression levels analysis
P67	qPderg10A-F	GCTGTTATCAAAGCCAACG	PCR primers for amplification of *ERG10A* gene for the
P68	qPderg10A-R	GGAGCAAGGTGACGAGGA	expression levels analysis
P69	qPderg10B-F	CTTTCGACTACGAGATTGCC	PCR primers for amplification of *ERG37 gene for the*
P70	qPderg10B-R	CGGAGCTTCTCAGGGTTG	*expression levels analysis*
P71	qPderg12-F	TGCTCTGCACAGCGTTTA	PCR primers for amplification of *ERG12* gene for the
P72	qPderg12-R	AGGAAGCCGTGGTTGATA	expression levels analysis

P73	qPderg13-F	AGTCCTACGCTCGTATGCT	PCR primers for amplification of *ERG13* gene for the expression levels analysis
P74	qPderg13-R	TGGAGACCAGGGTTCACA	
P75	qPderg20-F	CCTGGATGCTTCTTATGG	PCR primers for amplification of *ERG20* gene for the expression levels analysis
P76	qPderg20-R	ACTTCCTTCTTCAGACCCT	
P77	qPderg26-F	CGTGCTATGTGGGCTTTG	PCR primers for amplification of *ERG26* gene for the expression levels analysis
P78	qPderg26-R	ACTGTCCGGGCAGTGAGA	
P79	qPderg27-F	GCCGTTTCTACTGAGGTCG	PCR primers for amplification of *ERG27* gene for the expression levels analysis
P80	qPderg27-R	TCTTCCCTCTTGTCTTTGGT	
P81	qPdhmg1-F	CAACGCTCATGCCTCTAA	PCR primers for amplification of *hmg1* gene for the expression levels analysis
P82	qPdhmg1-R	CAATGGTTCCCACCTCAAT	
P83	qPdhyd1-F	GCGACCAGTTTGTTTGAC	PCR primers for amplification of *hyd1 gene* for the expression levels analysis
P84	qPdhyd1-R	GATGGTGGAGATACTGTGG	
P85	qPdidi1-F	GGGCATCAAGGCAGAGCA	PCR primers for amplification of *idi1 gene* for the expression levels analysis
P86	qPdidi1-R	TCGGCTGAGACGTAGGTTGT	
P87	qPdmvd1-F	CGTCAGTGCCGAGAAGAA	PCR primers for amplification of *mvd1* gene for the expression levels analysis
P88	qPdmvd1-R	AGTTGCGGGTTTGAATGG	
P89	qPdsmt1-F	TTCGTCACGCAAGATTTCA	PCR primers for amplification of *smt1* gene for the expression levels analysis
P90	qPdsmt1-R	GGGTAGATCCTGGCACAGA	

[a]The respective restriction enzyme sites included in primers are listed in lowercase in the sequence.
