# Supplementary material for: Functional analysis of two sterol regulatory element binding proteins in Penicillium digitatum
Source: PLoS One. 2017 May 3;12(5):e0176485. doi: 10.1371/journal.pone.0176485 (PMC5415137; doi:10.1371/journal.pone.0176485)
Supplement: S2 Table — (PDF) [file pone.0176485.s006.pdf]

S2 Table. Annotation of genes commonly up-regulated or down-regulated in all three mutants

ID	log2FC in △PdsreA	log2FC in △PdsreB	log2FC in △PdsreAB	nr_annotation
47 common elements in "△PdsreA", "△PdsreB" and "△PdsreAB":				
PDIG_22810	8.28	8.49	5.40	Triacylglycerol lipase (LipA), putative [Penicillium digitatum PHI26]
PDIG_84400	7.58	2.65	4.00	hypothetical protein PDIG_84400 [Penicillium digitatum PHI26]
PDIG_54620	7.43	8.52	2.20	2,4-dihydroxyhept-2-ene-1,7-dioic acid aldolase, putative [Penicillium digitatum PHI26]
PDIG_54640	7.34	8.27	1.84	Gibberellin 3-beta hydroxylase, putative [Penicillium digitatum PHI26]
PDIG_65980	7.05	4.45	6.47	hypothetical protein PDIG_33240 [Penicillium digitatum PHI26]
PDIG_54630	6.55	8.09	2.24	hypothetical protein PDIG_54630 [Penicillium digitatum PHI26]
PDIG_46550	6.25	3.79	1.69	Formate dehydrogenase [Penicillium digitatum PHI26]
PDIG_35500	4.99	4.28	2.51	Catalase [Penicillium digitatum PHI26]
PDIG_48410	4.43	4.69	1.78	putative pectin lyase 2 [Penicillium digitatum]
PDIG_03520	4.35	2.35	2.12	hypothetical protein PDIG_03520 [Penicillium digitatum PHI26]
PDIG_91100	4.22	3.91	2.51	L-asparaginase [Penicillium digitatum PHI26]
PDIG_00830	4.13	3.56	2.08	MFS monosaccharide transporter, putative [Penicillium digitatum PHI26]
PDIG_50400	4.09	3.54	1.39	Adenosine deaminase [Penicillium digitatum PHI26]
PDIG_36540	3.74	3.14	1.64	Purine nucleoside permease, putative [Penicillium digitatum Pd1]
PDIG_51490	3.61	4.04	2.49	hypothetical protein PDIG_51490 [Penicillium digitatum PHI26]
PDIG_01900	3.06	1.73	2.31	Asparagine synthetase Asn2, putative [Penicillium digitatum PHI26]
PDIG_51150	3.05	4.00	1.69	Glycosyl hydrolase, putative [Penicillium digitatum PHI26]
PDIG_90800	3.04	3.13	2.42	Cellobiose dehydrogenase [Penicillium digitatum PHI26]
PDIG_30080	2.95	2.69	1.54	RecName: Full=Polygalacturonase; Short=PG; AltName: Full=Pectinase; Flags: Precursor
PDIG_36670	2.79	2.33	1.36	Formaldehyde dehydrogenase [Penicillium digitatum Pd1]
PDIG_60720	2.53	2.03	1.66	hypothetical protein PDIP_70150 [Penicillium digitatum Pd1]

PDIG_65660	2.27	2.39	1.50	Dihydrodipicolinate synthetase family protein [Penicillium digitatum Pd1]
PDIG_81110	2.26	1.78	1.37	2OG-Fe(II) oxygenase superfamily protein [Aspergillus kawachii IFO 4308]
PDIG_02310	2.20	1.79	1.45	NADP-specific glutamate dehydrogenase [Penicillium digitatum PHI26]
PDIG_05470	2.06	1.92	1.40	Naphthalene 1,2-dioxygenase subunit alpha [Penicillium digitatum PHI26]
PDIG_23250	2.05	2.58	1.76	Pc13g06590 [Penicillium chrysogenum Wisconsin 54-1255]
PDIG_81860	2.04	3.03	1.75	hypothetical protein PDIG_81860 [Penicillium digitatum PHI26]
PDIG_88030	1.95	1.67	2.49	hypothetical protein PDIG_88030 [Penicillium digitatum PHI26]
PDIG_04480	1.94	2.18	4.19	Dihydrofolate reductase [Penicillium digitatum PHI26]
PDIG_20080	1.88	2.46	1.70	Pc20g07470 [Penicillium chrysogenum Wisconsin 54-1255]
PDIG_29700	1.87	4.03	4.25	hypothetical protein PDIG_29700 [Penicillium digitatum PHI26]
PDIG_91230	1.80	3.44	2.38	hypothetical protein PDIG_91230 [Penicillium digitatum PHI26]
PDIG_38960	1.69	2.55	2.75	hypothetical protein PDIG_38960 [Penicillium digitatum PHI26]
PDIG_32650	1.59	2.33	2.51	Pyridoxine biosynthesis protein pyroA [Penicillium digitatum Pd1]
PDIG_39430	1.55	1.69	2.47	Acetyltransferase [Penicillium digitatum PHI26]
PDIG_90070	1.54	2.33	3.08	Acetyltransferase, GNAT family [Penicillium digitatum PHI26]
PDIG_09590	1.47	2.35	1.84	Ubiquitin conjugating enzyme (UbcF), putative [Penicillium digitatum Pd1]
PDIG_26870	1.45	2.16	1.66	Allergen, putative [Penicillium digitatum Pd1]
PDIG_52870	1.44	1.61	1.95	Cysteine synthase [Penicillium digitatum PHI26]
PDIG_62090	1.37	1.99	1.57	S-adenosylmethionine-dependent methyltransferase, putative [Penicillium digitatum Pd1]
PDIG_76470	1.31	1.67	2.39	Formyltetrahydrofolate deformylase, putative [Penicillium digitatum PHI26]
PDIG_67250	1.28	1.73	3.71	Acetyltransferase, GNAT family [Penicillium digitatum Pd1]
PDIG_67880	1.23	3.22	1.92	hypothetical protein PDIP_77180 [Penicillium digitatum Pd1]
PDIG_15430	1.22	1.97	1.68	Adenylyl-sulfate kinase [Penicillium digitatum Pd1]
PDIG_34930	1.20	1.98	3.35	hypothetical protein PDIP_54490 [Penicillium digitatum Pd1]
PDIG_88120	1.12	1.74	2.39	saccharopine reductase [Penicillium chrysogenum]
PDIG_68240	1.10	2.61	2.09	Profilin [Penicillium digitatum Pd1]

105 common elements in "△PdsreA", "△PdsreB" and "△PdsreAB":				
PDIG_20650	-1.07	-6.60	-4.48	Thioredoxin [Penicillium digitatum Pd1]
PDIG_33240	-1.09	-3.25	-8.74	hypothetical protein PDIG_33240 [Penicillium digitatum PHI26]
PDIG_47170	-1.13	-5.64	-5.96	hypothetical protein PDIG_47170 [Penicillium digitatum PHI26]
PDIG_87570	-1.14	-3.15	-2.10	C6 transcription factor, putative [Penicillium digitatum PHI26]
PDIG_31060	-1.16	-2.73	-1.87	hypothetical protein PDIP_50640 [Penicillium digitatum Pd1]
PDIG_77040	-1.16	-2.43	-1.74	hypothetical protein PDIG_77040 [Penicillium digitatum PHI26]
PDIG_55560	-1.17	-2.68	-1.67	Hydroxamate-type ferrichrome siderophore peptide synthetase [Penicillium digitatum PHI26]
PDIG_48720	-1.17	-2.87	-1.95	Alpha-1,2-mannosidase, putative [Penicillium digitatum PHI26]
PDIG_75520	-1.22	-2.41	-1.77	Glutamate decarboxylase [Penicillium digitatum PHI26]
PDIG_87590	-1.22	-3.09	-2.27	hypothetical protein PDIG_87590 [Penicillium digitatum PHI26]
PDIG_14940	-1.24	-2.15	-1.42	Heat shock protein/chaperonin HSP78, putative [Penicillium digitatum PHI26]
PDIG_10200	-1.24	-2.49	-1.53	hypothetical protein PDIG_10200 [Penicillium digitatum PHI26]
PDIG_79420	-1.25	-2.73	-2.83	hypothetical protein PDIG_79420 [Penicillium digitatum PHI26]
PDIG_17680	-1.27	-1.75	-1.43	unnamed protein product [Aspergillus oryzae RIB40]
PDIG_00340	-1.30	-2.47	-1.79	Serine carboxypeptidase (CpdS), putative [Penicillium digitatum Pd1]
PDIG_66400	-1.30	-3.73	-3.62	hypothetical protein PDIP_75700 [Penicillium digitatum Pd1]
PDIG_35400	-1.31	-2.36	-1.46	Heat shock protein 90 [Penicillium digitatum Pd1]
PDIG_58780	-1.32	-3.40	-2.01	Heat shock protein, putative [Penicillium digitatum Pd1]
PDIG_85070	-1.36	-2.05	-1.74	hypothetical protein PDIG_85070 [Penicillium digitatum PHI26]
PDIG_51790	-1.41	-2.15	-2.42	Cyclin (Pcl1), putative [Penicillium digitatum PHI26]
PDIG_58230	-1.42	-2.92	-1.47	Pc14g00450 [Penicillium chrysogenum Wisconsin 54-1255]
PDIG_74400	-1.43	-2.28	-1.63	DNA repair protein Rad50 [Penicillium digitatum PHI26]
PDIG_52070	-1.45	-2.11	-1.76	Cytochrome P450 sterol C-22 desaturase, putative [Penicillium digitatum PHI26]
PDIG_14820	-1.50	-1.82	-4.14	C4-dicarboxylate transporter/malic acid transport protein, putative [Penicillium digitatum PHI26]

PDIG_83050	-1.51	-1.64	-1.84	hypothetical protein PDIP_86840 [Penicillium digitatum Pd1]
PDIG_09190	-1.53	-3.10	-2.05	hypothetical protein PDIP_37220 [Penicillium digitatum Pd1]
PDIG_81150	-1.53	-2.97	-1.64	hypothetical protein PDIG_81150 [Penicillium digitatum PHI26]
PDIG_79590	-1.55	-4.22	-1.57	2,3-diketo-5-methylthio-1-phosphopentane phosphatase, putative [Penicillium digitatum Pd1]
PDIG_23060	-1.55	-2.30	-2.91	hypothetical protein PDIG_23060 [Penicillium digitatum PHI26]
PDIG_16880	-1.62	-2.10	-1.56	General amidase GmdB [Penicillium digitatum Pd1]
PDIG_80130	-1.63	-2.51	-2.29	hypothetical protein PDIG_80130 [Penicillium digitatum PHI26]
PDIG_81000	-1.65	-3.36	-1.80	hypothetical protein PDIG_81000 [Penicillium digitatum PHI26]
PDIG_12700	-1.65	-2.89	-2.52	ATP citrate lyase, subunit 1, putative [Penicillium digitatum Pd1]
PDIG_74670	-1.67	-3.19	-1.61	hypothetical protein PDIG_74670 [Penicillium digitatum PHI26]
PDIG_48540	-1.67	-2.40	-1.75	MFS multidrug transporter, putative [Penicillium digitatum Pd1]
PDIG_47180	-1.69	-3.58	-4.43	hypothetical protein PDIG_47180 [Penicillium digitatum PHI26]
PDIG_85420	-1.69	-4.66	-2.35	14-alpha sterol demethylase [Penicillium digitatum]
PDIG_82290	-1.69	-2.23	-1.32	Choline oxidase (CodA), putative [Penicillium digitatum Pd1]
PDIG_57560	-1.71	-2.04	-1.96	Bli-3 protein, putative [Penicillium digitatum Pd1]
PDIG_19210	-1.71	-2.22	-1.67	hypothetical protein PDIG_19210 [Penicillium digitatum PHI26]
PDIG_79580	-1.74	-6.00	-3.04	Phosphorylase [Penicillium digitatum PHI26]
PDIG_02320	-1.75	-2.28	-1.46	Mediator of RNA polymerase II transcription subunit 5 [Penicillium digitatum PHI26]
PDIG_06200	-1.77	-2.90	-1.72	autophagy protein [Aspergillus kawachii IFO 4308]
PDIG_20480	-1.87	-6.71	-4.10	hypothetical protein PDIG_20480 [Penicillium digitatum PHI26]
PDIG_42270	-1.90	-2.42	-1.49	hypothetical protein PDIG_42270 [Penicillium digitatum PHI26]
PDIG_02990	-1.91	-3.78	-1.44	General amino acid permease (Agp2), putative [Penicillium digitatum PHI26]
PDIG_48520	-1.92	-4.00	-1.81	hypothetical protein PDIP_57890 [Penicillium digitatum Pd1]
PDIG_18320	-1.93	-3.39	-2.46	hypothetical protein PDIP_56150 [Penicillium digitatum Pd1]
PDIG_17890	-1.94	-3.53	-2.86	Pentachlorophenol 4-monooxygenase, putative [Penicillium digitatum Pd1]
PDIG_74660	-2.02	-4.12	-2.32	ABC transporter, putative [Penicillium digitatum PHI26]

PDIG_12710	-2.09	-1.95	-1.34	ATP citrate lyase subunit (Acl), putatibe [Penicillium digitatum Pd1]
PDIG_03140	-2.13	-3.71	-1.79	hypothetical protein PDIP_41790 [Penicillium digitatum Pd1]
PDIG_47150	-2.15	-4.97	-4.33	hypothetical protein PDIG_47150 [Penicillium digitatum PHI26]
PDIG_90250	-2.20	-5.89	-1.40	Aminopeptidase [Penicillium digitatum PHI26]
PDIG_70990	-2.21	-4.95	-2.14	hypothetical protein PDIP_80310 [Penicillium digitatum Pd1]
PDIG_86840	-2.22	-2.24	-1.45	hypothetical protein PDIG_86840 [Penicillium digitatum PHI26]
PDIG_10600	-2.25	-2.45	-1.33	Pc21g04140 [Penicillium chrysogenum Wisconsin 54-1255]
PDIG_48170	-2.28	-3.32	-4.13	hypothetical protein PDIP_57550 [Penicillium digitatum Pd1]
PDIG_63600	-2.29	-1.81	-2.68	hypothetical protein PDIG_63600 [Penicillium digitatum PHI26]
PDIG_63230	-2.30	-2.29	-1.42	hypothetical protein PDIP_72610 [Penicillium digitatum Pd1]
PDIG_21480	-2.40	-3.17	-1.73	Flavin-binding monooxygenase, putative [Penicillium digitatum PHI26]
PDIG_50840	-2.46	-2.39	-1.60	3-methyl-2-oxobutanoate hydroxymethyltransferase PanB [Penicillium digitatum PHI26]
PDIG_28880	-2.53	-4.11	-2.58	hypothetical protein PDIP_63320 [Penicillium digitatum Pd1]
PDIG_70870	-2.59	-3.43	-1.91	hypothetical protein PDIP_80190 [Penicillium digitatum Pd1]
PDIG_26220	-2.62	-4.69	-2.54	hypothetical protein PDIP_60700 [Penicillium digitatum Pd1]
PDIG_57930	-2.63	-6.04	-1.30	Bifunctional P450:NADPH-P450 reductase [Penicillium digitatum Pd1]
PDIG_43010	-2.67	-3.17	-2.89	hypothetical protein PDIG_43010 [Penicillium digitatum PHI26]
PDIG_58080	-2.71	-3.73	-2.06	Alpha,alpha-trehalose glucohydrolase TreA/Ath1 [Penicillium digitatum Pd1]
PDIG_16190	-2.72	-4.90	-2.66	Fatty acid synthase subunit alpha, putative [Penicillium digitatum Pd1]
PDIG_32750	-2.73	-6.85	-3.07	hypothetical protein PDIG_32750 [Penicillium digitatum PHI26]
PDIG_15450	-2.76	-2.85	-2.43	hypothetical protein PDIG_15450 [Penicillium digitatum PHI26]
PDIG_03410	-2.88	-3.57	-4.29	hypothetical protein PDIG_03410 [Penicillium digitatum PHI26]
PDIG_22270	-2.89	-3.58	-2.00	hypothetical protein PDIG_22270 [Penicillium digitatum PHI26]
PDIG_81790	-2.89	-7.58	-1.54	hypothetical protein PDIG_81790 [Penicillium digitatum PHI26]
PDIG_70830	-2.91	-4.45	-5.06	cytochrome P-450 14DM [Penicillium digitatum]
PDIG_24590	-2.98	-3.68	-1.85	hypothetical protein PDIG_24590 [Penicillium digitatum PHI26]

PDIG_21490	-3.00	-4.92	-1.68	hypothetical protein PDIG_21490 [Penicillium digitatum PHI26]
PDIG_57830	-3.01	-4.58	-1.69	hypothetical protein PDIP_67340 [Penicillium digitatum Pd1]
PDIG_75350	-3.17	-2.84	-2.88	NADPH oxidase (NoxA), putative [Penicillium digitatum PHI26]
PDIG_00120	-3.20	-8.12	-4.48	NADP-dependent alcohol dehydrogenase [Grosmannia clavigera kw1407]
PDIG_75660	-3.22	-3.94	-1.90	Endo-1,4-beta-xylanase, putative [Penicillium digitatum PHI26]
PDIG_32950	-3.30	-3.85	-3.32	Squalene epoxidase-like protein [Penicillium digitatum Pd1]
PDIG_16160	-3.34	-3.62	-2.60	Benzoate 4-monooxygenase cytochrome P450 [Penicillium digitatum Pd1]
PDIG_52750	-3.49	-2.95	-1.79	Metallo-beta-lactamase superfamily protein [Penicillium digitatum PHI26]
PDIG_83640	-3.51	-4.70	-1.85	MFS monocarboxylate transporter, putative [Penicillium digitatum Pd1]
PDIG_56310	-3.52	-3.35	-4.07	hypothetical protein PDIP_65870 [Penicillium digitatum Pd1]
PDIG_68830	-3.54	-3.78	-1.36	hypothetical protein PDIP_78110 [Penicillium digitatum Pd1]
PDIG_70500	-3.96	-5.31	-4.60	hypothetical protein PDIP_79810 [Penicillium digitatum Pd1]
PDIG_33180	-4.01	-4.12	-1.57	hypothetical protein PDIP_52770 [Penicillium digitatum Pd1]
PDIG_70460	-4.02	-3.37	-2.47	DNA photolyase, putative [Penicillium digitatum Pd1]
PDIG_76380	-4.17	-7.02	-3.36	hypothetical protein PDIG_76380 [Penicillium digitatum PHI26]
PDIG_83570	-4.18	-6.23	-2.39	MFS multidrug transporter, putative [Penicillium digitatum Pd1]
PDIG_45330	-4.27	-3.72	-2.51	hypothetical protein PDIG_45330 [Penicillium digitatum PHI26]
PDIG_02350	-4.41	-6.45	-1.82	ABC transporter, putative [Penicillium digitatum PHI26]
PDIG_16180	-4.47	-3.97	-3.65	Branched-chain amino acid aminotransferase [Penicillium digitatum Pd1]
PDIG_66050	-4.73	-7.73	-3.26	hypothetical protein PDIP_75350 [Penicillium digitatum Pd1]
PDIG_68750	-4.90	-2.50	-2.97	Glycosyl hydrolase, putative [Penicillium digitatum Pd1]
PDIG_42030	-4.93	-5.52	-6.62	Aspergillopepsin, putative [Penicillium digitatum PHI26]
PDIG_01990	-5.13	-7.04	-3.16	hypothetical protein PDIG_01990 [Penicillium digitatum PHI26]
PDIG_66060	-5.48	-9.28	-3.89	hypothetical protein PDIG_66060 [Penicillium digitatum PHI26]
PDIG_64840	-5.49	-4.50	-1.44	hypothetical protein PDIP_74170 [Penicillium digitatum Pd1]
PDIG_18140	-6.83	-6.52	-6.76	putative RNA-directed DNA polymerase from transposon X-element [Penicillium digitatum PHI26]

PDIG_15540	-6.86	-7.22	-3.65	Polyamine acetyltransferase [Penicillium digitatum PHI26]
PDIG_16300	-7.43	-7.12	-7.36	hypothetical protein PDIG_33240 [Penicillium digitatum PHI26]
PDIG_11580	-8.79	-8.48	-8.72	Retrotransposon polyprotein, putative [Penicillium digitatum PHI26]
