# Supplementary material for: Functional analysis of two sterol regulatory element binding proteins in Penicillium digitatum
Source: PLoS One. 2017 May 3;12(5):e0176485. doi: 10.1371/journal.pone.0176485 (PMC5415137; doi:10.1371/journal.pone.0176485)
Supplement: S3 Table — (PDF) [file pone.0176485.s007.pdf]

S3 Table. GO enrichment of differentially expressed genes

Δ*Pdsre*A_Up-regulated

Gene ID	Protein name	GO ID	GO name	GO namespace	Evidence code	Exact p-value	Adjusted p-value
PDIG_00830	MFS monosaccharide transporter, putative	GO:0055085	transmembrane transport	BP	IEA	5.28E-05	0.00934
PDIG_03520	Uncharacterized protein	GO:0055085	transmembrane transport	BP	IEA	5.28E-05	0.00934
PDIG_03870	Amine transporter, putative	GO:0055085	transmembrane transport	BP	IEA	5.28E-05	0.00934
PDIG_04910	MFS transporter, putative	GO:0055085	transmembrane transport	BP	IEA	5.28E-05	0.00934
PDIG_06950	Mitochondrial cation transporter, putative	GO:0055085	transmembrane transport	BP	IEA	5.28E-05	0.00934
PDIG_08180	Proline-specific permease	GO:0055085	transmembrane transport	BP	IEA	5.28E-05	0.00934
PDIG_09300	Efflux pump antibiotic resistance protein, putative	GO:0055085	transmembrane transport	BP	IEA	5.28E-05	0.00934
PDIG_15350	Uncharacterized protein	GO:0055085	transmembrane transport	BP	IEA	5.28E-05	0.00934
PDIG_16670	Uncharacterized protein	GO:0055085	transmembrane transport	BP	IEA	5.28E-05	0.00934
PDIG_17100	MFS monosaccharide transporter, putative	GO:0055085	transmembrane transport	BP	IEA	5.28E-05	0.00934
PDIG_24560	Putative transporter mch1	GO:0055085	transmembrane transport	BP	IEA	5.28E-05	0.00934
PDIG_26350	Uncharacterized protein	GO:0055085	transmembrane transport	BP	IEA	5.28E-05	0.00934
PDIG_27140	MFS transporter, putative	GO:0055085	transmembrane transport	BP	IEA	5.28E-05	0.00934
PDIG_34250	ABC transporter, putative	GO:0055085	transmembrane transport	BP	IEA	5.28E-05	0.00934
PDIG_36540	Purine nucleoside permease, putative	GO:0055085	transmembrane transport	BP	IEA	5.28E-05	0.00934
PDIG_37780	Amino acid permease (Dip5), putative	GO:0055085	transmembrane transport	BP	IEA	5.28E-05	0.00934
PDIG_40560	Oligopeptide transporter, putative	GO:0055085	transmembrane transport	BP	IEA	5.28E-05	0.00934
PDIG_40960	MFS transporter (Mch2), putative	GO:0055085	transmembrane transport	BP	IEA	5.28E-05	0.00934
PDIG_44800	MFS monosaccharide transporter, putative	GO:0055085	transmembrane transport	BP	IEA	5.28E-05	0.00934
PDIG_45710	ABC drug exporter AtrF	GO:0055085	transmembrane transport	BP	IEA	5.28E-05	0.00934
PDIG_46700	Uncharacterized protein	GO:0055085	transmembrane transport	BP	IEA	5.28E-05	0.00934
PDIG_48560	Nucleoside transporter, putative	GO:0055085	transmembrane transport	BP	IEA	5.28E-05	0.00934

PDIG_51830	Uncharacterized protein	GO:0055085	transmembrane transport	BP	IEA	5.28E-05	0.00934
PDIG_54610	Uncharacterized protein	GO:0055085	transmembrane transport	BP	IEA	5.28E-05	0.00934
PDIG_56670	MFS allantoate transporter, putative	GO:0055085	transmembrane transport	BP	IEA	5.28E-05	0.00934
PDIG_58110	Uncharacterized protein	GO:0055085	transmembrane transport	BP	IEA	5.28E-05	0.00934
PDIG_62410	Uncharacterized protein	GO:0055085	transmembrane transport	BP	IEA	5.28E-05	0.00934
PDIG_71910	Sodium/phosphate symporter, putative	GO:0055085	transmembrane transport	BP	IEA	5.28E-05	0.00934
PDIG_76170	MFS multidrug transporter, putative	GO:0055085	transmembrane transport	BP	IEA	5.28E-05	0.00934
PDIG_77560	Uncharacterized protein	GO:0055085	transmembrane transport	BP	IEA	5.28E-05	0.00934
PDIG_78380	Oligonucleotide transporter	GO:0055085	transmembrane transport	BP	IEA	5.28E-05	0.00934
PDIG_79150	Protein HOL1, putative	GO:0055085	transmembrane transport	BP	IEA	5.28E-05	0.00934
PDIG_82760	MFS transporter, putative	GO:0055085	transmembrane transport	BP	IEA	5.28E-05	0.00934
PDIG_84170	MFS myo-inositol transporter, putative	GO:0055085	transmembrane transport	BP	IEA	5.28E-05	0.00934
PDIG_84400	Uncharacterized protein	GO:0055085	transmembrane transport	BP	IEA	5.28E-05	0.00934
PDIG_85320	Purine permease, putative	GO:0055085	transmembrane transport	BP	IEA	5.28E-05	0.00934
PDIG_88160	Pantothenate transporter	GO:0055085	transmembrane transport	BP	IEA	5.28E-05	0.00934
PDIG_00200	Choline transporter Hnm1, putative	GO:0003333	amino acid transmembrane transport	BP	IEA	0.000466	0.02291
PDIG_08180	Proline-specific permease	GO:0003333	amino acid transmembrane transport	BP	IEA	0.000466	0.02291
PDIG_25750	GABA permease, putative	GO:0003333	amino acid transmembrane transport	BP	IEA	0.000466	0.02291
PDIG_29590	Amino acid permease	GO:0003333	amino acid transmembrane transport	BP	IEA	0.000466	0.02291
PDIG_37780	Amino acid permease (Dip5), putative	GO:0003333	amino acid transmembrane transport	BP	IEA	0.000466	0.02291
PDIG_67790	Uncharacterized protein	GO:0003333	amino acid transmembrane	BP	IEA	0.000466	0.02291

			transport				
PDIG_78430	GabA permease, putative	GO:0003333	amino acid transmembrane transport	BP	IEA	0.000466	0.02291
PDIG_84400	Uncharacterized protein	GO:0003333	amino acid transmembrane transport	BP	IEA	0.000466	0.02291
PDIG_89860	Uncharacterized protein	GO:0003333	amino acid transmembrane transport	BP	IEA	0.000466	0.02291
PDIG_00420	Beta-hexosaminidase (EC 3.2.1.52)	GO:0005975	carbohydrate metabolic process	BP	IEA	0.000492	0.02291
PDIG_03380	Pectin lyase B	GO:0005975	carbohydrate metabolic process	BP	IEA	0.000492	0.02291
PDIG_13580	Extracellular endoglucanase, putative	GO:0005975	carbohydrate metabolic process	BP	IEA	0.000492	0.02291
PDIG_14140	1,3-beta-glucanosyltransferase gel2	GO:0005975	carbohydrate metabolic process	BP	IEA	0.000492	0.02291
PDIG_21170	Aldose 1-epimerase, putative	GO:0005975	carbohydrate metabolic process	BP	IEA	0.000492	0.02291
PDIG_28100	Exopolygalacturonase X	GO:0005975	carbohydrate metabolic process	BP	IEA	0.000492	0.02291
PDIG_30080	Polygalacturonase	GO:0005975	carbohydrate metabolic process	BP	IEA	0.000492	0.02291
PDIG_30830	Extracellular endoglucanase/cellulase, putative	GO:0005975	carbohydrate metabolic process	BP	IEA	0.000492	0.02291
PDIG_33090	Putative beta-glucosidase G	GO:0005975	carbohydrate metabolic process	BP	IEA	0.000492	0.02291
PDIG_36090	Uncharacterized protein	GO:0005975	carbohydrate metabolic process	BP	IEA	0.000492	0.02291
PDIG_45070	Polygalacturonase-2	GO:0005975	carbohydrate metabolic process	BP	IEA	0.000492	0.02291
PDIG_46690	Putative intracellular invertase	GO:0005975	carbohydrate metabolic process	BP	IEA	0.000492	0.02291
PDIG_48410	Pectin lyase, putative	GO:0005975	carbohydrate metabolic process	BP	IEA	0.000492	0.02291
PDIG_51800	Uncharacterized protein	GO:0005975	carbohydrate metabolic process	BP	IEA	0.000492	0.02291
PDIG_58160	Extracellular invertase	GO:0005975	carbohydrate metabolic process	BP	IEA	0.000492	0.02291
PDIG_59450	Arabinogalactan endo-1,4-beta-galactosidase GalA	GO:0005975	carbohydrate metabolic process	BP	IEA	0.000492	0.02291
PDIG_68670	Beta-glucosidase	GO:0005975	carbohydrate metabolic process	BP	IEA	0.000492	0.02291
PDIG_76480	Glucanase (EC 3.2.1.-)	GO:0005975	carbohydrate metabolic process	BP	IEA	0.000492	0.02291
PDIG_82710	1,3-beta-glucanosyltransferase gel4	GO:0005975	carbohydrate metabolic process	BP	IEA	0.000492	0.02291

PDIG_89390	Putative beta-glucosidase btgE	GO:0005975	carbohydrate metabolic process	BP	IEA	0.000492	0.02291
**Δ*PdsreA*_Down-regulated**							
Gene ID	Protein name	GO ID	GO name	GO namespace	Evidence code	Exact p-value	Adjusted p-value
PDIG_14940	Heat shock protein/chaperonin HSP78, putative	GO:0006950	response to stress	BP	IEA	1.25E-05	0.00378
PDIG_16120	Uncharacterized protein	GO:0006950	response to stress	BP	IEA	1.25E-05	0.00378
PDIG_35400	Heat shock protein 90	GO:0006950	response to stress	BP	IEA	1.25E-05	0.00378
PDIG_40570	Heat shock 70 kDa protein	GO:0006950	response to stress	BP	IEA	1.25E-05	0.00378
PDIG_58780	Heat shock protein, putative	GO:0006950	response to stress	BP	IEA	1.25E-05	0.00378
PDIG_78570	Heat shock protein Hsp88, putative	GO:0006950	response to stress	BP	IEA	1.25E-05	0.00378
PDIG_82110	Heat shock protein	GO:0006950	response to stress	BP	IEA	1.25E-05	0.00378
PDIG_88130	Uncharacterized protein	GO:0006950	response to stress	BP	IEA	1.25E-05	0.00378
**Δ*PdsreB*_Up-regulated**							
Gene ID	Protein name	GO ID	GO name	GO namespace	Evidence code	Exact p-value	Adjusted p-value
PDIG_19790	Cytochrome c oxidase subunit 6A, mitochondrial (Cytochrome c oxidase polypeptide VIa)	GO:1902600	hydrogen ion transmembrane transport	BP	IEA	3.9E-06	0.00058
PDIG_43930	Cytochrome c oxidase polypeptide vib	GO:1902600	hydrogen ion transmembrane transport	BP	IEA	3.9E-06	0.00058
PDIG_62630	Uncharacterized protein	GO:1902600	hydrogen ion transmembrane transport	BP	IEA	3.9E-06	0.00058
PDIG_78150	Ubiquinol-cytochrome C reductase complex subunit UcrQ, putative	GO:1902600	hydrogen ion transmembrane transport	BP	IEA	3.9E-06	0.00058
PDIG_88080	Cytochrome b-c1 complex subunit 6	GO:1902600	hydrogen ion transmembrane transport	BP	IEA	3.9E-06	0.00058
PDIG_M0010	Cytochrome c oxidase subunit 1 (EC 1.9.3.1)	GO:1902600	hydrogen ion transmembrane	BP	IEA	3.9E-06	0.00058

			transport				
PDIG_M0040	Cytochrome c oxidase subunit 2	GO:1902600	hydrogen ion transmembrane transport	BP	IEA	3.9E-06	0.00058
PDIG_60830	Uncharacterized protein	GO:0006334	nucleosome assembly	BP	IEA	2.39E-05	0.00088
PDIG_62650	Uncharacterized protein	GO:0006334	nucleosome assembly	BP	IEA	2.39E-05	0.00088
PDIG_64680	Uncharacterized protein	GO:0006334	nucleosome assembly	BP	IEA	2.39E-05	0.00088
PDIG_64730	Uncharacterized protein	GO:0006334	nucleosome assembly	BP	IEA	2.39E-05	0.00088
PDIG_67850	Uncharacterized protein	GO:0006334	nucleosome assembly	BP	IEA	2.39E-05	0.00088
PDIG_01950	Cytochrome b-c1 complex subunit 7	GO:0006122	mitochondrial electron transport, ubiquinol to cytochrome c	BP	IEA	6.87E-05	0.00201
PDIG_39160	Uncharacterized protein	GO:0006122	mitochondrial electron transport, ubiquinol to cytochrome c	BP	IEA	6.87E-05	0.00201
PDIG_88080	Cytochrome b-c1 complex subunit 6	GO:0006122	mitochondrial electron transport, ubiquinol to cytochrome c	BP	IEA	6.87E-05	0.00201
PDIG_03380	Pectin lyase B	GO:0000272	polysaccharide catabolic process	BP	IEA	0.000241	0.00543
PDIG_36510	Uncharacterized protein	GO:0000272	polysaccharide catabolic process	BP	IEA	0.000241	0.00543
PDIG_48410	Pectin lyase, putative	GO:0000272	polysaccharide catabolic process	BP	IEA	0.000241	0.00543
PDIG_51800	Uncharacterized protein	GO:0000272	polysaccharide catabolic process	BP	IEA	0.000241	0.00543
PDIG_76480	Glucanase (EC 3.2.1.-)	GO:0000272	polysaccharide catabolic process	BP	IEA	0.000241	0.00543
PDIG_35270	Esterase, putative	GO:0046294	formaldehyde catabolic process	BP	IEA	0.001685	0.02351
PDIG_85730	Putative glutathione-dependent formaldehyde-activating enzyme (EC 4.4.1.22) (S-(hydroxymethyl)glutathione synthase)	GO:0046294	formaldehyde catabolic process	BP	IEA	0.001685	0.02351
PDIG_23740	Uncharacterized protein	GO:0006850	mitochondrial pyruvate transport	BP	IEA	0.001685	0.02351
PDIG_28090	Uncharacterized protein	GO:0006850	mitochondrial pyruvate transport	BP	IEA	0.001685	0.02351
PDIG_05290	Mitochondrial metallochaperone Sco1, putative	GO:0006825	copper ion transport	BP	IEA	0.001685	0.02351

PDIG_05420	Cytochrome c oxidase copper chaperone Cox17, putative	GO:0006825	copper ion transport	BP	IEA	0.001685	0.02351
PDIG_00950	Peptidyl-prolyl cis-trans isomerase (EC 5.2.1.8)	GO:0006457	protein folding	BP	IEA	0.002159	0.02751
PDIG_15390	Peptidyl-prolyl cis-trans isomerase	GO:0006457	protein folding	BP	IEA	0.002159	0.02751
PDIG_22800	Peptidyl-prolyl cis-trans isomerase	GO:0006457	protein folding	BP	IEA	0.002159	0.02751
PDIG_35820	Prefoldin subunit 4	GO:0006457	protein folding	BP	IEA	0.002159	0.02751
PDIG_43890	GrpE protein homolog	GO:0006457	protein folding	BP	IEA	0.002159	0.02751
PDIG_47500	Protein disulfide-isomerase (EC 5.3.4.1)	GO:0006457	protein folding	BP	IEA	0.002159	0.02751
PDIG_61790	Peptidyl-prolyl cis-trans isomerase (EC 5.2.1.8)	GO:0006457	protein folding	BP	IEA	0.002159	0.02751
PDIG_00950	Peptidyl-prolyl cis-trans isomerase (EC 5.2.1.8)	GO:0000413	protein peptidyl-prolyl isomerization	BP	IEA	0.002657	0.03243
PDIG_15390	Peptidyl-prolyl cis-trans isomerase	GO:0000413	protein peptidyl-prolyl isomerization	BP	IEA	0.002657	0.03243
PDIG_22800	Peptidyl-prolyl cis-trans isomerase	GO:0000413	protein peptidyl-prolyl isomerization	BP	IEA	0.002657	0.03243
PDIG_61790	Peptidyl-prolyl cis-trans isomerase (EC 5.2.1.8)	GO:0000413	protein peptidyl-prolyl isomerization	BP	IEA	0.002657	0.03243
PDIG_11920	Putative ATP synthase protein 9	GO:0015991	ATP hydrolysis coupled proton transport	BP	IEA	0.003429	0.03721
PDIG_32160	Vacuolar ATPase proteolipid subunit c, putative	GO:0015991	ATP hydrolysis coupled proton transport	BP	IEA	0.003429	0.03721
PDIG_64700	Vacuolar ATP synthase 16 kDa proteolipid subunit, putative	GO:0015991	ATP hydrolysis coupled proton transport	BP	IEA	0.003429	0.03721
PDIG_72090	V-ATPase proteolipid subunit Ppa1, putative	GO:0015991	ATP hydrolysis coupled proton transport	BP	IEA	0.003429	0.03721
PDIG_50400	Adenine deaminase (ADE) (EC 3.5.4.2) (Adenine aminohydrolase)	GO:0009117	nucleotide metabolic process	BP	IEA	0.004917	0.04968

PDIG_84580	Inosine triphosphate pyrophosphatase (ITPase) (Inosine triphosphatase) (EC 3.6.1.19) (Non-canonical purine NTP pyrophosphatase) (Non-standard purine NTP pyrophosphatase) (Nucleoside-triphosphate diphosphatase) (Nucleoside-triphosphate pyrophosphatase)	GO:0009117	nucleotide metabolic process	BP	IEA	0.004917	0.04968
**Δ*PdsreB*_Down-regulated**							
Gene ID	Protein name	GO ID	GO name	GO namespace	Evidence code	Exact p-value	Adjusted p-value
PDIG_06440 | PDIG_25360 | PDIG_33240	Uncharacterized protein	GO:0015074	DNA integration	BP	IEA	5.948e-7	0.00023
PDIG_11580	Retrotransposon polyprotein, putative	GO:0015074	DNA integration	BP	IEA	5.948e-7	0.00023
PDIG_11590	Retrotransposon polyprotein, putative	GO:0015074	DNA integration	BP	IEA	5.948e-7	0.00023
PDIG_16300	Uncharacterized protein	GO:0015074	DNA integration	BP	IEA	5.948e-7	0.00023
PDIG_39030 | PDIG_91320	Uncharacterized protein	GO:0015074	DNA integration	BP	IEA	5.948e-7	0.00023
PDIG_47150	Uncharacterized protein	GO:0015074	DNA integration	BP	IEA	5.948e-7	0.00023
PDIG_49840	Uncharacterized protein	GO:0015074	DNA integration	BP	IEA	5.948e-7	0.00023
PDIG_55890	Uncharacterized protein	GO:0015074	DNA integration	BP	IEA	5.948e-7	0.00023
PDIG_68720 | PDIG_71260	Uncharacterized protein	GO:0015074	DNA integration	BP	IEA	5.948e-7	0.00023
PDIG_74650	Uncharacterized protein	GO:0015074	DNA integration	BP	IEA	5.948e-7	0.00023

PDIG_77400	Retrovirus-related Pol polyprotein from transposon TNT 1-94	GO:0015074	DNA integration	BP	IEA	5.948e-7	0.00023
PDIG_78050	Transposable element tc3 transposase, putative	GO:0015074	DNA integration	BP	IEA	5.948e-7	0.00023
PDIG_79200	Uncharacterized protein	GO:0015074	DNA integration	BP	IEA	5.948e-7	0.00023
PDIG_89880	Uncharacterized protein	GO:0015074	DNA integration	BP	IEA	5.948e-7	0.00023
PDIG_91200	Uncharacterized protein	GO:0015074	DNA integration	BP	IEA	5.948e-7	0.00023
PDIG_02350	ABC transporter, putative	GO:0055085	transmembrane transport	BP	IEA	3.16E-05	0.0062
PDIG_02690	Amino acid permease, putative	GO:0055085	transmembrane transport	BP	IEA	3.16E-05	0.0062
PDIG_02990	General amino acid permease (Agp2), putative	GO:0055085	transmembrane transport	BP	IEA	3.16E-05	0.0062
PDIG_08000	Proline permease, putative	GO:0055085	transmembrane transport	BP	IEA	3.16E-05	0.0062
PDIG_08590	MFS phospholipid transporter (Git1), putative	GO:0055085	transmembrane transport	BP	IEA	3.16E-05	0.0062
PDIG_11800	MFS multidrug transporter, putative	GO:0055085	transmembrane transport	BP	IEA	3.16E-05	0.0062
PDIG_12860	MFS amine transporter, putative	GO:0055085	transmembrane transport	BP	IEA	3.16E-05	0.0062
PDIG_14180	Amino acid permease (Can1), putative	GO:0055085	transmembrane transport	BP	IEA	3.16E-05	0.0062
PDIG_14820	C4-dicarboxylate transporter/malic acid transport protein, putative	GO:0055085	transmembrane transport	BP	IEA	3.16E-05	0.0062
PDIG_14870	Calcium channel subunit Cch1	GO:0055085	transmembrane transport	BP	IEA	3.16E-05	0.0062
PDIG_16130	ABC transporter, putative	GO:0055085	transmembrane transport	BP	IEA	3.16E-05	0.0062
PDIG_20480	Uncharacterized protein	GO:0055085	transmembrane transport	BP	IEA	3.16E-05	0.0062
PDIG_26350	Uncharacterized protein	GO:0055085	transmembrane transport	BP	IEA	3.16E-05	0.0062
PDIG_26840	Uncharacterized protein	GO:0055085	transmembrane transport	BP	IEA	3.16E-05	0.0062
PDIG_31620	MFS multidrug transporter, putative	GO:0055085	transmembrane transport	BP	IEA	3.16E-05	0.0062
PDIG_32420	Uncharacterized protein	GO:0055085	transmembrane transport	BP	IEA	3.16E-05	0.0062
PDIG_32430	Uncharacterized protein	GO:0055085	transmembrane transport	BP	IEA	3.16E-05	0.0062
PDIG_34520	MFS transporter, putative	GO:0055085	transmembrane transport	BP	IEA	3.16E-05	0.0062
PDIG_34690	Uncharacterized protein	GO:0055085	transmembrane transport	BP	IEA	3.16E-05	0.0062

PDIG_35660	Uncharacterized protein	GO:0055085	transmembrane transport	BP	IEA	3.16E-05	0.0062
PDIG_36420	MFS transporter, putative	GO:0055085	transmembrane transport	BP	IEA	3.16E-05	0.0062
PDIG_36560	MFS transporter, putative	GO:0055085	transmembrane transport	BP	IEA	3.16E-05	0.0062
PDIG_38440	MFS allantoate transporter, putative	GO:0055085	transmembrane transport	BP	IEA	3.16E-05	0.0062
PDIG_39610	Uncharacterized protein	GO:0055085	transmembrane transport	BP	IEA	3.16E-05	0.0062
PDIG_42350	Uncharacterized protein	GO:0055085	transmembrane transport	BP	IEA	3.16E-05	0.0062
PDIG_46460	Uncharacterized protein	GO:0055085	transmembrane transport	BP	IEA	3.16E-05	0.0062
PDIG_47290	MFS multidrug transporter, putative	GO:0055085	transmembrane transport	BP	IEA	3.16E-05	0.0062
PDIG_47310	MFS monosaccharide transporter, putative	GO:0055085	transmembrane transport	BP	IEA	3.16E-05	0.0062
PDIG_48540	MFS multidrug transporter, putative	GO:0055085	transmembrane transport	BP	IEA	3.16E-05	0.0062
PDIG_48690	Uncharacterized protein	GO:0055085	transmembrane transport	BP	IEA	3.16E-05	0.0062
PDIG_49480	Plasma membrane antiporter, putative	GO:0055085	transmembrane transport	BP	IEA	3.16E-05	0.0062
PDIG_49990	ABC multidrug transporter, putative	GO:0055085	transmembrane transport	BP	IEA	3.16E-05	0.0062
PDIG_50830	Sulfate permease SutB	GO:0055085	transmembrane transport	BP	IEA	3.16E-05	0.0062
PDIG_55710	MFS transporter, putative	GO:0055085	transmembrane transport	BP	IEA	3.16E-05	0.0062
PDIG_55720	MFS multidrug transporter, putative	GO:0055085	transmembrane transport	BP	IEA	3.16E-05	0.0062
PDIG_59910	MFS multidrug transporter, putative	GO:0055085	transmembrane transport	BP	IEA	3.16E-05	0.0062
PDIG_60750	Sugar transporter, putative	GO:0055085	transmembrane transport	BP	IEA	3.16E-05	0.0062
PDIG_67650	Uncharacterized protein	GO:0055085	transmembrane transport	BP	IEA	3.16E-05	0.0062
PDIG_68190	Amino acid transporter, putative	GO:0055085	transmembrane transport	BP	IEA	3.16E-05	0.0062
PDIG_68530	2-ketogluconate transporter, putative	GO:0055085	transmembrane transport	BP	IEA	3.16E-05	0.0062
PDIG_70130	Uncharacterized protein	GO:0055085	transmembrane transport	BP	IEA	3.16E-05	0.0062
PDIG_73390	MFS multidrug resistance transporter, putative	GO:0055085	transmembrane transport	BP	IEA	3.16E-05	0.0062
PDIG_74660	ABC transporter, putative	GO:0055085	transmembrane transport	BP	IEA	3.16E-05	0.0062
PDIG_74670	Uncharacterized protein	GO:0055085	transmembrane transport	BP	IEA	3.16E-05	0.0062
PDIG_77390	Uncharacterized protein	GO:0055085	transmembrane transport	BP	IEA	3.16E-05	0.0062

PDIG_77810	Mechanosensitive ion channel family	GO:0055085	transmembrane transport	BP	IEA	3.16E-05	0.0062
PDIG_80370	Uncharacterized protein	GO:0055085	transmembrane transport	BP	IEA	3.16E-05	0.0062
PDIG_82720	Vacuolar H+/Ca2+ exchanger	GO:0055085	transmembrane transport	BP	IEA	3.16E-05	0.0062
PDIG_83570	MFS multidrug transporter, putative	GO:0055085	transmembrane transport	BP	IEA	3.16E-05	0.0062
PDIG_83640	MFS monocarboxylate transporter, putative	GO:0055085	transmembrane transport	BP	IEA	3.16E-05	0.0062
PDIG_86770	Uncharacterized protein	GO:0055085	transmembrane transport	BP	IEA	3.16E-05	0.0062
PDIG_88550	ABC multidrug transporter, putative	GO:0055085	transmembrane transport	BP	IEA	3.16E-05	0.0062
PDIG_90700	ABC multidrug transporter, putative	GO:0055085	transmembrane transport	BP	IEA	3.16E-05	0.0062
PDIG_14940	Heat shock protein/chaperonin HSP78, putative	GO:0006950	response to stress	BP	IEA	8.62E-05	0.00845
PDIG_16120	Uncharacterized protein	GO:0006950	response to stress	BP	IEA	8.62E-05	0.00845
PDIG_35400	Heat shock protein 90	GO:0006950	response to stress	BP	IEA	8.62E-05	0.00845
PDIG_35910	Heat shock protein Hsp98/Hsp104/ClpA, putative	GO:0006950	response to stress	BP	IEA	8.62E-05	0.00845
PDIG_40570	Heat shock 70 kDa protein	GO:0006950	response to stress	BP	IEA	8.62E-05	0.00845
PDIG_58780	Heat shock protein, putative	GO:0006950	response to stress	BP	IEA	8.62E-05	0.00845
PDIG_78570	Heat shock protein Hsp88, putative	GO:0006950	response to stress	BP	IEA	8.62E-05	0.00845
PDIG_82110	Heat shock protein	GO:0006950	response to stress	BP	IEA	8.62E-05	0.00845
PDIG_86500	Thiamine thiazole synthase (Thiazole biosynthetic enzyme)	GO:0006950	response to stress	BP	IEA	8.62E-05	0.00845
PDIG_02690	Amino acid permease, putative	GO:0003333	amino acid transmembrane transport	BP	IEA	0.001025	0.04068
PDIG_02990	General amino acid permease (Agp2), putative	GO:0003333	amino acid transmembrane transport	BP	IEA	0.001025	0.04068
PDIG_08000	Proline permease, putative	GO:0003333	amino acid transmembrane transport	BP	IEA	0.001025	0.04068
PDIG_14180	Amino acid permease (Can1), putative	GO:0003333	amino acid transmembrane transport	BP	IEA	0.001025	0.04068

PDIG_32420	Uncharacterized protein	GO:0003333	amino acid transmembrane transport	BP	IEA	0.001025	0.04068
PDIG_32430	Uncharacterized protein	GO:0003333	amino acid transmembrane transport	BP	IEA	0.001025	0.04068
PDIG_34460	Uncharacterized protein	GO:0003333	amino acid transmembrane transport	BP	IEA	0.001025	0.04068
PDIG_34470	GABA permease, putative	GO:0003333	amino acid transmembrane transport	BP	IEA	0.001025	0.04068
PDIG_35660	Uncharacterized protein	GO:0003333	amino acid transmembrane transport	BP	IEA	0.001025	0.04068
PDIG_42120	Uncharacterized protein	GO:0003333	amino acid transmembrane transport	BP	IEA	0.001025	0.04068
PDIG_68190	Amino acid transporter, putative	GO:0003333	amino acid transmembrane transport	BP	IEA	0.001025	0.04068
Δ*PdsreAB*_Up-regulated							
PDIG_01900	Asparagine synthetase (EC 6.3.5.4)	GO:0008652	cellular amino acid biosynthetic process	BP	IEA	2.91E-06	0.00099
PDIG_04670	Aspartate-semialdehyde dehydrogenase	GO:0008652	cellular amino acid biosynthetic process	BP	IEA	2.91E-06	0.00099
PDIG_32620	Arginine biosynthesis bifunctional protein ArgJ, mitochondrial	GO:0008652	cellular amino acid biosynthetic process	BP	IEA	2.91E-06	0.00099
PDIG_47340	Amino-acid acetyltransferase, mitochondrial (EC 2.3.1.1) (Glutamate N-acetyltransferase) (N-acetylglutamate synthase)	GO:0008652	cellular amino acid biosynthetic process	BP	IEA	2.91E-06	0.00099
PDIG_59400	Chorismate synthase (EC 4.2.3.5)	GO:0008652	cellular amino acid biosynthetic process	BP	IEA	2.91E-06	0.00099

PDIG_65050	Saccharopine dehydrogenase [NAD(+), L-lysine-forming] (SDH) (EC 1.5.1.7) (Lysine--2-oxoglutarate reductase)	GO:0008652	cellular amino acid biosynthetic process	BP	IEA	2.91E-06	0.00099
PDIG_78070	Multifunctional tryptophan biosynthesis protein	GO:0008652	cellular amino acid biosynthetic process	BP	IEA	2.91E-06	0.00099
PDIG_81970	Homoserine dehydrogenase (HDH) (EC 1.1.1.3)	GO:0008652	cellular amino acid biosynthetic process	BP	IEA	2.91E-06	0.00099
PDIG_87700	L-aminoadipate-semialdehyde dehydrogenase (EC 1.2.1.31) (Alpha-aminoadipate reductase)	GO:0008652	cellular amino acid biosynthetic process	BP	IEA	2.91E-06	0.00099
PDIG_04540	Uncharacterized protein	GO:0042823	pyridoxal phosphate biosynthetic process	BP	IEA	7.65E-05	0.0052
PDIG_32650	Pyridoxine biosynthesis protein pyroA	GO:0042823	pyridoxal phosphate biosynthetic process	BP	IEA	7.65E-05	0.0052
PDIG_38050	Pyridoxine	GO:0042823	pyridoxal phosphate biosynthetic process	BP	IEA	7.65E-05	0.0052
PDIG_48160	Pyridoxamine phosphate oxidase, putative	GO:0042823	pyridoxal phosphate biosynthetic process	BP	IEA	7.65E-05	0.0052
PDIG_39230	Catalytic activity: homocitrate synthases convert 2-hydroxybutane-1	GO:0019878	lysine biosynthetic process via aminoadipic acid	BP	IEA	0.000114	0.00649
PDIG_65050	Saccharopine dehydrogenase [NAD(+), L-lysine-forming] (SDH) (EC 1.5.1.7) (Lysine--2-oxoglutarate reductase)	GO:0019878	lysine biosynthetic process via aminoadipic acid	BP	IEA	0.000114	0.00649
PDIG_87700	L-aminoadipate-semialdehyde dehydrogenase (EC 1.2.1.31) (Alpha-aminoadipate reductase)	GO:0019878	lysine biosynthetic process via aminoadipic acid	BP	IEA	0.000114	0.00649
PDIG_03450	40S ribosomal protein S10a	GO:0006412	translation	BP	IEA	0.000225	0.0085
PDIG_04290	60S ribosomal protein L35Ae	GO:0006412	translation	BP	IEA	0.000225	0.0085
PDIG_23880	60S ribosomal protein L38, putative	GO:0006412	translation	BP	IEA	0.000225	0.0085
PDIG_29800	40S ribosomal protein S14 (CRP2)	GO:0006412	translation	BP	IEA	0.000225	0.0085

PDIG_29930	40S ribosomal protein S17, putative	GO:0006412	translation	BP	IEA	0.000225	0.0085
PDIG_31150	60S ribosomal protein L30-2	GO:0006412	translation	BP	IEA	0.000225	0.0085
PDIG_37250	60S ribosomal protein L29, putative	GO:0006412	translation	BP	IEA	0.000225	0.0085
PDIG_37860	Uncharacterized protein	GO:0006412	translation	BP	IEA	0.000225	0.0085
PDIG_38600	Uncharacterized protein	GO:0006412	translation	BP	IEA	0.000225	0.0085
PDIG_48970	Ribosomal protein S28e	GO:0006412	translation	BP	IEA	0.000225	0.0085
PDIG_55910	40S ribosomal protein S22	GO:0006412	translation	BP	IEA	0.000225	0.0085
PDIG_60480	40S ribosomal protein S26E	GO:0006412	translation	BP	IEA	0.000225	0.0085
PDIG_60950	40S ribosomal protein S11	GO:0006412	translation	BP	IEA	0.000225	0.0085
PDIG_62610	Ribosomal protein L34 protein, putative	GO:0006412	translation	BP	IEA	0.000225	0.0085
PDIG_64480	Uncharacterized protein	GO:0006412	translation	BP	IEA	0.000225	0.0085
PDIG_66320	Aspartyl-tRNA synthetase Dps1, putative	GO:0006412	translation	BP	IEA	0.000225	0.0085
PDIG_84640	60S ribosomal protein L23	GO:0006412	translation	BP	IEA	0.000225	0.0085
PDIG_90480	Tryptophanyl-tRNA synthetase	GO:0006412	translation	BP	IEA	0.000225	0.0085
PDIG_32620	Arginine biosynthesis bifunctional protein ArgJ, mitochondrial	GO:0006526	arginine biosynthetic process	BP	IEA	0.000441	0.01501
PDIG_47340	Amino-acid acetyltransferase, mitochondrial (EC 2.3.1.1) (Glutamate N-acetyltransferase) (N-acetylglutamate synthase)	GO:0006526	arginine biosynthetic process	BP	IEA	0.000441	0.01501
PDIG_70490	Argininosuccinate synthase	GO:0006526	arginine biosynthetic process	BP	IEA	0.000441	0.01501
PDIG_62650	Uncharacterized protein	GO:0006334	nucleosome assembly	BP	IEA	0.000917	0.02834
PDIG_64680	Uncharacterized protein	GO:0006334	nucleosome assembly	BP	IEA	0.000917	0.02834
PDIG_64730	Uncharacterized protein	GO:0006334	nucleosome assembly	BP	IEA	0.000917	0.02834
PDIG_67850	Uncharacterized protein	GO:0006334	nucleosome assembly	BP	IEA	0.000917	0.02834
PDIG_14740	Valyl-tRNA synthetase	GO:0006418	tRNA aminoacylation for protein translation	BP	IEA	0.001487	0.04213

PDIG_31600	Uncharacterized protein	GO:0006418	tRNA aminoacylation for protein translation	BP	IEA	0.001487	0.04213
PDIG_33890	Cysteinyl-tRNA synthetase	GO:0006418	tRNA aminoacylation for protein translation	BP	IEA	0.001487	0.04213
PDIG_66320	Aspartyl-tRNA synthetase Dps1, putative	GO:0006418	tRNA aminoacylation for protein translation	BP	IEA	0.001487	0.04213
PDIG_73310	Uncharacterized protein	GO:0006418	tRNA aminoacylation for protein translation	BP	IEA	0.001487	0.04213
PDIG_83190	Cytoplasmic asparaginyl-tRNA synthetase, putative	GO:0006418	tRNA aminoacylation for protein translation	BP	IEA	0.001487	0.04213
PDIG_90480	Tryptophanyl-tRNA synthetase	GO:0006418	tRNA aminoacylation for protein translation	BP	IEA	0.001487	0.04213
PDIG_41200	Anthranilate phosphoribosyltransferase, putative	GO:0000162	tryptophan biosynthetic process	BP	IEA	0.002051	0.04235
PDIG_43660	Bifunctional tryptophan synthase TRPB	GO:0000162	tryptophan biosynthetic process	BP	IEA	0.002051	0.04235
PDIG_78070	Multifunctional tryptophan biosynthesis protein	GO:0000162	tryptophan biosynthetic process	BP	IEA	0.002051	0.04235
PDIG_01500	Ornithine carbamoyltransferase	GO:0006520	cellular amino acid metabolic process	BP	IEA	0.002241	0.04235
PDIG_02310	Glutamate dehydrogenase	GO:0006520	cellular amino acid metabolic process	BP	IEA	0.002241	0.04235
PDIG_03740	Threonine synthase Thr4, putative	GO:0006520	cellular amino acid metabolic process	BP	IEA	0.002241	0.04235
PDIG_81970	Homoserine dehydrogenase (HDH) (EC 1.1.1.3)	GO:0006520	cellular amino acid metabolic process	BP	IEA	0.002241	0.04235
PDIG_91100	L-asparaginase	GO:0006520	cellular amino acid metabolic process	BP	IEA	0.002241	0.04235
PDIG_23740	Uncharacterized protein	GO:0006850	mitochondrial pyruvate transport	BP	IEA	0.002367	0.04235

PDIG_28090	Uncharacterized protein	GO:0006850	mitochondrial pyruvate transport	BP	IEA	0.002367	0.04235
Δ*PdsreAB*_Down-regulated							
PDIG_33240	Uncharacterized protein	GO:0015074	DNA integration	BP	IEA	5.6029e-10	1.7929e-7
PDIG_07800	Uncharacterized protein	GO:0015074	DNA integration	BP	IEA	5.6029e-10	1.7929e-7
PDIG_11210	Uncharacterized protein	GO:0015074	DNA integration	BP	IEA	5.6029e-10	1.7929e-7
PDIG_11580	Retrotransposon polyprotein, putative	GO:0015074	DNA integration	BP	IEA	5.6029e-10	1.7929e-7
PDIG_11590	Retrotransposon polyprotein, putative	GO:0015074	DNA integration	BP	IEA	5.6029e-10	1.7929e-7
PDIG_16300	Uncharacterized protein	GO:0015074	DNA integration	BP	IEA	5.6029e-10	1.7929e-7
PDIG_33120	Gag/polymerase/env polyprotein, putative	GO:0015074	DNA integration	BP	IEA	5.6029e-10	1.7929e-7
PDIG_39030 | PDIG_91320	Uncharacterized protein	GO:0015074	DNA integration	BP	IEA	5.6029e-10	1.7929e-7
PDIG_47150	Uncharacterized protein	GO:0015074	DNA integration	BP	IEA	5.6029e-10	1.7929e-7
PDIG_49840	Uncharacterized protein	GO:0015074	DNA integration	BP	IEA	5.6029e-10	1.7929e-7
PDIG_68720 | PDIG_71260	Uncharacterized protein	GO:0015074	DNA integration	BP	IEA	5.6029e-10	1.7929e-7
PDIG_73760	Retrovirus polyprotein, putative	GO:0015074	DNA integration	BP	IEA	5.6029e-10	1.7929e-7
PDIG_74650	Uncharacterized protein	GO:0015074	DNA integration	BP	IEA	5.6029e-10	1.7929e-7
PDIG_79200	Uncharacterized protein	GO:0015074	DNA integration	BP	IEA	5.6029e-10	1.7929e-7
PDIG_89880	Uncharacterized protein	GO:0015074	DNA integration	BP	IEA	5.6029e-10	1.7929e-7
PDIG_91200	Uncharacterized protein	GO:0015074	DNA integration	BP	IEA	5.6029e-10	1.7929e-7
PDIG_07040	Sphinganine hydroxylase Sur2, putative	GO:0006633	fatty acid biosynthetic process	BP	IEA	7.52E-05	0.00601
PDIG_21160	Uncharacterized protein	GO:0006633	fatty acid biosynthetic process	BP	IEA	7.52E-05	0.00601
PDIG_22330	C-4 methyl sterol oxidase Erg25, putative	GO:0006633	fatty acid biosynthetic process	BP	IEA	7.52E-05	0.00601
PDIG_33180	Uncharacterized protein	GO:0006633	fatty acid biosynthetic process	BP	IEA	7.52E-05	0.00601

PDIG_37910	Acetyl-CoA carboxylase	GO:0006633	fatty acid biosynthetic process	BP	IEA	7.52E-05	0.00601
PDIG_46910	Sterol delta 5,6-desaturase ERG3	GO:0006633	fatty acid biosynthetic process	BP	IEA	7.52E-05	0.00601
PDIG_56130	Fatty acid hydroxylase, putative	GO:0006633	fatty acid biosynthetic process	BP	IEA	7.52E-05	0.00601
PDIG_14310	Kinesin family protein (KipA), putative	GO:0007018	microtubule-based movement	BP	IEA	0.000334	0.01526
PDIG_24440	Kinesin family protein	GO:0007018	microtubule-based movement	BP	IEA	0.000334	0.01526
PDIG_24530	Kinesin family protein	GO:0007018	microtubule-based movement	BP	IEA	0.000334	0.01526
PDIG_62450	Kinesin family protein	GO:0007018	microtubule-based movement	BP	IEA	0.000334	0.01526
PDIG_75000	Kinesin family protein	GO:0007018	microtubule-based movement	BP	IEA	0.000334	0.01526
PDIG_02360	Ras guanine-nucleotide exchange protein, putative	GO:0043547	positive regulation of GTPase activity	BP	IEA	0.000863	0.0251
PDIG_19160	Uncharacterized protein	GO:0043547	positive regulation of GTPase activity	BP	IEA	0.000863	0.0251
PDIG_49080	Rho guanyl nucleotide exchange factor, putative	GO:0043547	positive regulation of GTPase activity	BP	IEA	0.000863	0.0251
PDIG_51670	Uncharacterized protein	GO:0043547	positive regulation of GTPase activity	BP	IEA	0.000863	0.0251
PDIG_61710	Ras GTPase activating protein, putative	GO:0043547	positive regulation of GTPase activity	BP	IEA	0.000863	0.0251
PDIG_64750	Guanine nucleotide exchange factor, putative	GO:0043547	positive regulation of GTPase activity	BP	IEA	0.000863	0.0251
PDIG_25460	Rho guanyl nucleotide exchange factor, putative	GO:0035023	regulation of Rho protein signal transduction	BP	IEA	0.003243	0.04732
PDIG_32170	Rho guanyl nucleotide exchange factor (Rom2), putative	GO:0035023	regulation of Rho protein signal transduction	BP	IEA	0.003243	0.04732
PDIG_49080	Rho guanyl nucleotide exchange factor, putative	GO:0035023	regulation of Rho protein signal transduction	BP	IEA	0.003243	0.04732

PDIG_25460	Rho guanyl nucleotide exchange factor, putative	GO:0032321	positive regulation of Rho GTPase activity	BP	IEA	0.003243	0.04732
PDIG_32170	Rho guanyl nucleotide exchange factor (Rom2), putative	GO:0032321	positive regulation of Rho GTPase activity	BP	IEA	0.003243	0.04732
PDIG_49080	Rho guanyl nucleotide exchange factor, putative	GO:0032321	positive regulation of Rho GTPase activity	BP	IEA	0.003243	0.04732
PDIG_49470	Glutamate decarboxylase (EC 4.1.1.15)	GO:0006536	glutamate metabolic process	BP	IEA	0.003253	0.04732
PDIG_75520	Glutamate decarboxylase (EC 4.1.1.15)	GO:0006536	glutamate metabolic process	BP	IEA	0.003253	0.04732
PDIG_63820	Uncharacterized protein	GO:0015671	oxygen transport	BP	IEA	0.003253	0.04732
PDIG_67840	Bacterial hemoglobin	GO:0015671	oxygen transport	BP	IEA	0.003253	0.04732
PDIG_05360	Plasma membrane ATPase	GO:0006812	cation transport	BP	IEA	0.003677	0.04902
PDIG_37740	Phospholipid-transporting ATPase, putative	GO:0006812	cation transport	BP	IEA	0.003677	0.04902
PDIG_39390	Phospholipid-transporting ATPase (DRS2), putative	GO:0006812	cation transport	BP	IEA	0.003677	0.04902
PDIG_49050	Na/K ATPase alpha 1 subunit, putative	GO:0006812	cation transport	BP	IEA	0.003677	0.04902
PDIG_49480	Plasma membrane antiporter, putative	GO:0006812	cation transport	BP	IEA	0.003677	0.04902
PDIG_51660	Calcium ion transporter Vcx1, putative	GO:0006812	cation transport	BP	IEA	0.003677	0.04902
PDIG_78950	Calcium-transporting ATPase (EC 3.6.3.8)	GO:0006812	cation transport	BP	IEA	0.003677	0.04902
