# Supplementary material for: Functional analysis of two sterol regulatory element binding proteins in Penicillium digitatum
Source: PLoS One. 2017 May 3;12(5):e0176485. doi: 10.1371/journal.pone.0176485 (PMC5415137; doi:10.1371/journal.pone.0176485)
Supplement: S4 Table — (PDF) [file pone.0176485.s008.pdf]

**S4 Table. Putative SREs found in promoter regions of genes involved in ergosterol biosynthesis, *PdsreA* and *PdsreB*[a]**

| Gene | Putative SRE | Position | |
|---|---|---|---|
| | | Start | Stop |
| *ERG10A* | GTCATGACATT | -751 | -740 |
| *HMG2* | ATGGAATGATG | -1763 | -1752 |
| *ERG12* | ATGTGCTGACC | -1903 | -1892 |
| | ATCATCCCACC | -1104 | -1093 |
| | ATAATCCCATC | -1091 | -1080 |
| *IDI1* | ATGGGCTGATA | -1954 | -1943 |
| | ATCTTGACACC | -311 | -300 |
| *ERG1* | ATCAGTCCACT | -239 | -228 |
| | GTCACGCCACC | -157 | -146 |
| *CYP51A* | GTGGGAAGATA | -1804 | -1793 |
| | TGGCACCATCC | -1758 | -1747 |
| | ATGATGCCAGC | -965 | -954 |
| | ATGGCCTGACC | -132 | -121 |
| *CYP51B* | GTGGCGTGATA | -391 | -380 |
| *ERG25* | GTGGGGTGATC | -480 | -469 |
| | GTGGCCTTACC | -447 | -436 |
| *ERG27* | GTCGCCTGACG | -965 | -954 |
| *ERG3B* | ATGAGTACACT | -1288 | -1277 |
| *ERG4B* | TTGTGCTGATC | -1001 | -990 |
| *PdsreA* | ATCATACGACC | -636 | -626 |
| *PdsreB* | GTCACCCCACT | -1793 | -1783 |

[a]Sequences of putative SREs in the promoters of genes in egosterol biosynthetic pathway are listed together with their position relative to the start codon of ORF. The search was based on the SRE motif found in *A. fumigatus*.
