# Supplementary material for: Functional analysis of two sterol regulatory element binding proteins in Penicillium digitatum
Source: PLoS One. 2017 May 3;12(5):e0176485. doi: 10.1371/journal.pone.0176485 (PMC5415137; doi:10.1371/journal.pone.0176485)
Supplement: S5 Table — (PDF) [file pone.0176485.s009.pdf]

S5 Table. Annotation of genes commonly regulated by *PdsreA* and *PdsreB*

ID	log2FC in △PdsreA	log2FC in △PdsreB	Pfam family	domain name(abbreviation)	domain name	nr_annotation
242 common elements in "△PdsreA-down" and "△PdsreB-down":						
PDIG_20650	-1.07	-6.60	PF00085,	Thioredoxin,	Thioredoxin,	Thioredoxin
PDIG_22250	-1.08	-1.68	PF05532,	CsbD,	CsbD-like,	Mismatched base pair and cruciform DNA recognition protein, putative
PDIG_33240	-1.09	-3.25	PF00665,PF07727,	rve,RVT_2,	Integrase core domain,Reverse transcriptase (RNA-dependent DNA polymerase),	hypothetical protein PDIG_33240
PDIG_36490	-1.12	-2.03	PF08031,PF01565,	BBE,FAD_binding_4	Berberine and berberine like,FAD binding domain,	hypothetical protein PDIP_83090
PDIG_47170	-1.13	-5.64	PF11917,	DUF3435,	Protein of unknown function (DUF3435),	hypothetical protein PDIG_47170
PDIG_87570	-1.14	-3.15	PF00172,PF04082,	Zn_clus,Fungal_trans,	Fungal Zn(2)-Cys(6) binuclear cluster domain,Fungal specific transcription factor domain,	C6 transcription factor, putative
PDIG_31060	-1.16	-2.73	PF04366,	Ysc84,	Las17-binding protein actin regulator,	hypothetical protein PDIP_50640
PDIG_77040	-1.16	-2.43	PF10637,PF13661,	Ofd1_CTDD,2OG-FeII_Oxy_4,	Oxoglutarate and iron-dependent oxygenase degradation C-term,2OG-Fe(II) oxygenase superfamily,	hypothetical protein PDIG_77040
PDIG_35760	-1.16	-4.19	PF11927,	DUF3445,	Protein of unknown function (DUF3445),	hypothetical protein PDIG_35760

PDIG_55560	-1.17	-2.68				Hydroxamate-type ferrichrome siderophore peptide synthetase
PDIG_23690	-1.17	-2.34	PF00117,PF06418,	GATase,CTP_synth_N,	Glutamine amidotransferase class-I,CTP synthase N-terminus,	CTP synthase, putative
PDIG_48720	-1.17	-2.87	PF07971,	Glyco_hydro_92,	Glycosyl hydrolase family 92,	Alpha-1,2-mannosidase, putative
PDIG_34840	-1.18	-2.08	PF13577,	SnoaL_4,	SnoaL-like domain,	hypothetical protein PDIP_54410
PDIG_72950	-1.19	-1.70	PF00702,PF00403,PF00122,	Hydrolase,HMA,E1-E2_ATPase,	haloacid dehalogenase-like hydrolase,Heavy-metal-associated domain,E1-E2 ATPase,	Copper-transporting ATPase, putative
PDIG_39610	-1.20	-1.95	PF00005,PF00664,	ABC_tran,ABC_membrane,	ABC transporter,ABC transporter transmembrane region,	hypothetical protein PDIG_39610
PDIG_75300	-1.21	-1.84	PF00621,	RhoGEF,	RhoGEF domain,	Rho guanyl nucleotide exchange factor, putative
PDIG_81160	-1.21	-3.70				hypothetical protein PDIG_81160
PDIG_90640	-1.21	-1.64	PF00168,	C2,	C2 domain,	putative serine proline-rich protein
PDIG_38250	-1.22	-3.09	PF01266,PF08669,PF01571,PF16350,	DAO,GCV_T_C,GCV_T,FAO_M,	FAD dependent oxidoreductase,Glycine cleavage T-protein C-terminal barrel domain,Aminomethyltransferase folate-binding domain,FAD dependent oxidoreductase central domain,	N,N-dimethylglycine oxidase, putative
PDIG_08230	-1.22	-3.54	PF05592,	Bac_rhamnosid,	Bacterial alpha-L-rhamnosidase,	Alpha-L-rhamnosidase A, putative
PDIG_75520	-1.22	-2.41	PF00282,	Pyridoxal_deC,	Pyridoxal-dependent decarboxylase conserved domain,	Glutamate decarboxylase
PDIG_87590	-1.22	-3.09	PF00168,	C2,	C2 domain,	hypothetical protein PDIG_87590

PDIG_14940	-1.24	-2.15	PF00004,PF10431, PF07724,	AAA,ClpB_D2-small ,AAA_2,	ATPase family associated with various cellular activities (AAA),C-terminal, D2-small domain, of ClpB protein,AAA domain (Cdc48 subfamily),	Heat shock protein/chaperonin HSP78, putative
PDIG_10200	-1.24	-2.49	PF05383,	La,	La domain,	hypothetical protein PDIG_10200
PDIG_41180	-1.24	-1.98	PF00388,	PI-PLC-X,	Phosphatidylinositol-specific phospholipase C, X domain,	Phosphatidylinositol phospholipase C
PDIG_79420	-1.25	-2.73				hypothetical protein PDIG_79420
PDIG_78570	-1.26	-2.15	PF00012,	HSP70,	Hsp70 protein,	Heat shock protein Hsp88, putative
PDIG_58530	-1.26	-1.73	PF00400,PF04192,	WD40,Utp21,	WD domain, G-beta repeat,Utp21 specific WD40 associated putative domain,	SnoRNA binding protein, putative
PDIG_17680	-1.27	-1.75	PF00078,	RVT_1,	Reverse transcriptase (RNA-dependent DNA polymerase),	unnamed protein product [Aspergillus oryzae RIB40]
PDIG_42350	-1.27	-3.05	PF01554,	MatE,	MatE,	hypothetical protein PDIG_42350
PDIG_75530	-1.28	-2.92	PF16177,PF00501,	ACAS_N,AMP-bindi ng,	Acetyl-coenzyme A synthetase N-terminus,AMP-binding enzyme,	Pc22g00960 [Penicillium chrysogenum Wisconsin 54-1255]
PDIG_87970	-1.29	-1.97	PF00096,	zf-C2H2,	Zinc finger, C2H2 type,	hypothetical protein PDIG_87970
PDIG_57720	-1.29	-1.88	PF00623,PF04997, PF04998,PF05000, PF04983,	RNA_pol_Rpb1_2,R NA_pol_Rpb1_1,RN A_pol_Rpb1_5,RNA _pol_Rpb1_4,RNA_p ol_Rpb1_3,	RNA polymerase Rpb1, domain 2,RNA polymerase Rpb1, domain 1,RNA polymerase Rpb1, domain 5,RNA polymerase Rpb1, domain 4,RNA polymerase Rpb1, domain 3,	DNA-directed RNA polymerase
PDIG_38440	-1.29	-2.39	PF07690,	MFS_1,	Major Facilitator Superfamily,	MFS allantoate transporter, putative

PDIG_00340	-1.30	-2.47	PF00450,	Peptidase_S10,	Serine carboxypeptidase,	Serine carboxypeptidase (CpdS), putative [Penicillium digitatum Pd1]
PDIG_26630	-1.30	-1.64	PF01053,	Cys_Met_Meta_PP,	Cys/Met metabolism PLP-dependent enzyme,	Cystathionine gamma-lyase
PDIG_66400	-1.30	-3.73				hypothetical protein PDIP_75700
PDIG_35400	-1.31	-2.36	PF00183,PF02518,	HSP90,HATPase_c,	Hsp90 protein,Histidine kinase-, DNA gyrase B-, and HSP90-like ATPase,	Heat shock protein 90
PDIG_14980	-1.31	-1.77				hypothetical protein PDIG_14980
PDIG_63550	-1.31	-1.73	PF02129,PF08530,	Peptidase_S15,PepX_C,	X-Pro dipeptidyl-peptidase (S15 family),X-Pro dipeptidyl-peptidase C-terminal non-catalytic domain,	Hydrolase, CocE/NonD family, putative
PDIG_82180	-1.32	-1.58	PF00009,PF00679, PF03764,PF16004, PF03144,	GTP_EFTU,EFG_C, EFG_IV,EFTUD2,GTP_EFTU_D2,	Elongation factor Tu GTP binding domain,Elongation factor G C-terminus,Elongation factor G, domain IV,116 kDa U5 small nuclear ribonucleoprotein component N-terminus,Elongation factor Tu domain 2,	U5 snRNP component Snu114, putative
PDIG_58780	-1.32	-3.40	PF00011,	HSP20,	Hsp20/alpha crystallin family,	Heat shock protein, putative
PDIG_36520	-1.33	-3.01	PF01494,	FAD_binding_3,	FAD binding domain,	Salicylate hydroxylase
PDIG_41980	-1.33	-3.56	PF00550,PF00668, PF00501,	PP-binding,Condensation,AMP-binding,	Phosphopantetheine attachment site,Condensation domain,AMP-binding enzyme,	Nonribosomal peptide synthase Pes1
PDIG_08590	-1.34	-2.46	PF00083,	Sugar_tr,	Sugar (and other) transporter,	MFS phospholipid transporter (Git1), putative
PDIG_39740	-1.36	-1.83				hypothetical protein PDIG_39740

PDIG_85070	-1.36	-2.05				hypothetical protein PDIG_85070
PDIG_14100	-1.36	-1.97	PF02787,PF00988, PF00117,PF02142, PF00185,PF02729, PF02786,	CPSase_L_D3,CPSase_sm_chain,GATase, MGS,OTCace,OTCace_N,CPSase_L_D2,	Carbamoyl-phosphate synthetase large chain, oligomerisation domain,Carbamoyl-phosphate synthase small chain, CPSase domain,Glutamine amidotransferase class-I,MGS-like domain,Aspartate/ornithine carbamoyltransferase, Asp/Orn binding domain,Aspartate/ornithine carbamoyltransferase, carbamoyl-P binding domain,Carbamoyl-phosphate synthase L chain, ATP binding domain,	Bifunctional pyrimidine biosynthesis protein (PyrABCN), putative
PDIG_83130	-1.38	-1.75	PF00067,	p450,	Cytochrome P450,	hypothetical protein PDIP_86910
PDIG_06490	-1.39	-4.76	PF00282,	Pyridoxal_deC,	Pyridoxal-dependent decarboxylase conserved domain,	hypothetical protein PDIG_06490
PDIG_31010	-1.39	-2.28	PF00226,PF01556,	DnaJ,DnaJ_C,	DnaJ domain,DnaJ C terminal domain,	hypothetical protein PDIG_31010
PDIG_31950	-1.41	-1.56				hypothetical protein PDIP_51530 [Penicillium digitatum Pd1]
PDIG_86040	-1.41	-2.40	PF03810,PF13646,	IBN_N,HEAT_2,	Importin-beta N-terminal domain,HEAT repeats,	Importin beta-4 subunit, putative
PDIG_51110	-1.41	-2.17	PF16651,PF00076,	RRM_u2,RRM_1,	Unstructured region on RNA-binding-domain protein,RNA recognition motif. (a.k.a. RRM, RBD, or RNP domain),	hypothetical protein PDIG_51110
PDIG_51790	-1.41	-2.15	PF00134,	Cyclin_N,	Cyclin, N-terminal domain,	Cyclin (Pcl1), putative
PDIG_81780	-1.42	-4.05	PF11807,	DUF3328,	Domain of unknown function	hypothetical protein PDIG_81780

					(DUF3328),	
PDIG_58230	-1.42	-2.92				Pc14g00450 [Penicillium chrysogenum Wisconsin 54-1255]
PDIG_40570	-1.43	-2.00				Heat shock 70 kDa protein
PDIG_74400	-1.43	-2.28				DNA repair protein Rad50
PDIG_62370	-1.44	-1.81				hypothetical protein PDIP_71750
PDIG_52070	-1.45	-2.11	PF00067,	p450,	Cytochrome P450,	Cytochrome P450 sterol C-22 desaturase, putative
PDIG_72780	-1.47	-1.70	PF14613,	DUF4449,	Protein of unknown function (DUF4449),	hypothetical protein PDIG_72780
PDIG_26600	-1.47	-2.95				hypothetical protein PDIP_61050
PDIG_11800	-1.47	-3.79				MFS multidrug transporter, putative
PDIG_07530	-1.48	-1.93	PF00085,PF08324, PF05903,	Thioredoxin,PUL,Peptidase_C97,	Thioredoxin,PUL domain,PPPDE putative peptidase domain,	Thioredoxin, putative
PDIG_72110	-1.49	-2.35	PF02862,	DDHD,	DDHD domain,	hypothetical protein AN7604.2 [Aspergillus nidulans FGSC A4]
PDIG_14180	-1.50	-1.91	PF00324,	AA_permease,	Amino acid permease,	Amino acid permease (Can1), putative
PDIG_14820	-1.50	-1.82	PF03595,	SLAC1,	Voltage-dependent anion channel,	C4-dicarboxylate transporter/malic acid transport protein, putative
PDIG_83050	-1.51	-1.64	PF00172,	Zn_clus,	Fungal Zn(2)-Cys(6) binuclear cluster domain,	hypothetical protein PDIP_86840
PDIG_03500	-1.51	-2.36				hypothetical protein PDIG_03500
PDIG_09190	-1.53	-3.10	PF13489,	Methyltransf_23,	Methyltransferase domain,	hypothetical protein PDIP_37220
PDIG_81150	-1.53	-2.97	PF00931,	NB-ARC,	NB-ARC domain,	hypothetical protein PDIG_81150
PDIG_34520	-1.55	-3.00	PF07690,	MFS_1,	Major Facilitator Superfamily,	MFS transporter, putative

PDIG_21300	-1.55	-2.11	PF00270,PF00271, PF13959,	DEAD,Helicase_C,DUF4217,	DEAD/DEAH box helicase,Helicase conserved C-terminal domain,Domain of unknown function (DUF4217),	DEAD/DEAH box helicase (Sbp4), putative
PDIG_79590	-1.55	-4.22	PF12710,	HAD,	haloacid dehalogenase-like hydrolase,	2,3-diketo-5-methylthio-1-phosphopentane phosphatase, putative [Penicillium digitatum Pd1]
PDIG_23060	-1.55	-2.30	PF08611,	DUF1774,	Fungal protein of unknown function (DUF1774),	hypothetical protein PDIG_23060
PDIG_45850	-1.56	-2.04	PF11799,PF00817,	IMS_C,IMS,	impB/mucB/samB family C-terminal domain,impB/mucB/samB family,	hypothetical protein PDIG_45850
PDIG_34340	-1.58	-5.13	PF00704,	Glyco_hydro_18,	Glycosyl hydrolases family 18,	Endochitinase 2
PDIG_39730	-1.59	-1.87	PF05920,	Homeobox_KN,	Homeobox KN domain,	Homeobox transcription factor, putative
PDIG_05710	-1.59	-2.87	PF13450,PF00106,	NAD_binding_8,adh_short,	NAD(P)-binding Rossmann-like domain,short chain dehydrogenase,	Flavin-containing amine oxidasedehydrogenase, putative
PDIG_04140	-1.60	-2.94	PF01987,	AIM24,	Mitochondrial biogenesis AIM24,	hypothetical protein PDIG_04140
PDIG_29310	-1.61	-3.00	PF06628,PF00199,	Catalase-rel,Catalase,	Catalase-related immune-responsive,Catalase,	Catalase
PDIG_44550	-1.61	-2.98	PF02458,	Transferase,	Transferase family,	hypothetical protein PDIG_44550
PDIG_16880	-1.62	-2.10	PF01425,	Amidase,	Amidase,	General amidase GmdB
PDIG_80130	-1.63	-2.51				hypothetical protein PDIG_80130
PDIG_65410	-1.63	-1.77	PF02798,PF14497,	GST_N,GST_C_3,	Glutathione S-transferase, N-terminal domain,Glutathione S-transferase, C-terminal domain,	Glutathione transferase 3
PDIG_81000	-1.65	-3.36	PF10241,	KxDL,	Uncharacterized conserved protein,	hypothetical protein PDIG_81000

PDIG_12700	-1.65	-2.89	PF02629,PF00285, PF00549,	CoA_binding,Citrate _synt,Ligase_CoA,	CoA binding domain,Citrate synthase, C-terminal domain,CoA-ligase,	ATP citrate lyase, subunit 1, putative
PDIG_60750	-1.66	-4.50	PF00083,	Sugar_tr,	Sugar (and other) transporter,	Sugar transporter, putative
PDIG_74670	-1.67	-3.19	PF07690,	MFS_1,	Major Facilitator Superfamily,	hypothetical protein PDIG_74670
PDIG_48540	-1.67	-2.40	PF07690,	MFS_1,	Major Facilitator Superfamily,	MFS multidrug transporter, putative
PDIG_32510	-1.67	-1.82	PF00293,	NUDIX,	NUDIX domain,	hypothetical protein [Penicillium chrysogenum Wisconsin 54-1255]
PDIG_26830	-1.69	-3.19				hypothetical protein PDIP_61280
PDIG_47180	-1.69	-3.58				hypothetical protein PDIG_47180
PDIG_85420	-1.69	-4.66	PF00067,	p450,	Cytochrome P450,	14-alpha sterol demethylase [Penicillium digitatum]
PDIG_82290	-1.69	-2.23	PF00732,PF05199,	GMC_oxred_N,GMC _oxred_C,	GMC oxidoreductase,GMC oxidoreductase,	Choline oxidase (CodA), putative
PDIG_16130	-1.70	-2.78	PF00005,PF00664,	ABC_tran,ABC_me mbrane,	ABC transporter,ABC transporter transmembrane region,	ABC transporter, putative
PDIG_57560	-1.71	-2.04	PF16242,	Pyrid_ox_like,	Pyridoxamine 5'-phosphate oxidase like,	Bli-3 protein, putative
PDIG_19210	-1.71	-2.22	PF00172,	Zn_clus,	Fungal Zn(2)-Cys(6) binuclear cluster domain,	hypothetical protein PDIG_19210
PDIG_73350	-1.72	-3.14				hypothetical protein PDIG_73350
PDIG_45860	-1.73	-2.59	PF00730,	HhH-GPD,	HhH-GPD superfamily base excision DNA repair protein,	Base excision DNA repair protein
PDIG_15470	-1.73	-3.30	PF05592,	Bac_rhamnosid,	Bacterial alpha-L-rhamnosidase,	Rhamnosidase B, putative
PDIG_79580	-1.74	-6.00	PF00343,	Phosphorylase,	Carbohydrate phosphorylase,	Phosphorylase
PDIG_02320	-1.75	-2.28	PF08689,	Med5,	Mediator complex subunit Med5,	Mediator of RNA polymerase II transcription subunit 5

PDIG_06200	-1.77	-2.90	PF11700,	ATG22,	Vacuole effluxer Atg22 like,	autophagy protein [Aspergillus kawachii IFO 4308]
PDIG_73520	-1.79	-2.43	PF00026,	Asp,	Eukaryotic aspartyl protease,	Aspartic endopeptidase (AP1), putative
PDIG_23680	-1.79	-1.97	PF06964,	Alpha-L-AF_C,	Alpha-L-arabinofuranosidase C-terminal domain,	Alpha-L-arabinofuranosidase, putative
PDIG_82110	-1.80	-1.85	PF00012,	HSP70,	Hsp70 protein,	Hsp70 chaperone (HscA), putative [Talaromyces stipitatus ATCC 10500]
PDIG_02280	-1.86	-2.75	PF13847,	Methyltransf_31,	Methyltransferase domain,	hypothetical protein PDIG_02280
PDIG_33940	-1.86	-1.67	PF13243,PF13249,	SQHop_cyclase_C,SQHop_cyclase_N,	Squalene-hopene cyclase C-terminal domain,Squalene-hopene cyclase N-terminal domain,	Oxidosqualene:lanosterol cyclase
PDIG_75670	-1.86	-2.03	PF11001,	DUF2841,	Protein of unknown function (DUF2841),	hypothetical protein PDIG_75670
PDIG_40470	-1.87	-2.01	PF00920,	ILVD_EDD,	Dehydratase family,	Dihydroxy-acid dehydratase, putative
PDIG_20480	-1.87	-6.71	PF03169,	OPT,	OPT oligopeptide transporter protein,	hypothetical protein PDIG_20480
PDIG_74430	-1.88	-3.40	PF00155,	Aminotran_1_2,	Aminotransferase class I and II,	hypothetical protein PDIG_74430
PDIG_20330	-1.88	-2.41				hypothetical protein PDIG_20330
PDIG_22940	-1.89	-2.03				hypothetical protein PDIG_22940
PDIG_42270	-1.90	-2.42	PF00172,PF04082,	Zn_clus,Fungal_trans,	Fungal Zn(2)-Cys(6) binuclear cluster domain,Fungal specific transcription factor domain,	hypothetical protein PDIG_42270
PDIG_02990	-1.91	-3.78	PF00324,	AA_permease,	Amino acid permease,	General amino acid permease (Agp2), putative

PDIG_04180	-1.92	-3.36	PF00076,	RRM_1,	RNA recognition motif. (a.k.a. RRM, RBD, or RNP domain),	hypothetical protein PDIG_04180
PDIG_48520	-1.92	-4.00				hypothetical protein PDIP_57890
PDIG_18320	-1.93	-3.39	PF05637,	Glyco_transf_34,	galactosyl transferase GMA12/MNN10 family,	hypothetical protein PDIP_56150
PDIG_17890	-1.94	-3.53	PF07976,PF01494, PF13561,	Phe_hydrox_dim,FAD_binding_3,adh_short_C2,	Phenol hydroxylase, C-terminal dimerisation domain,FAD binding domain,Enoyl-(Acyl carrier protein) reductase,	Pentachlorophenol 4-monooxygenase, putative
PDIG_54380	-1.94	-3.66				hypothetical protein PDIG_54380
PDIG_90260	-1.97	-4.70				hypothetical protein PDIG_90260
PDIG_86580	-1.97	-2.30	PF04563,PF04561, PF04560,PF04565, PF06883,PF00562,	RNA_pol_Rpb2_1,RNA_pol_Rpb2_2,RNA_pol_Rpb2_7,RNA_pol_Rpb2_3,RNA_pol_Rpa2_4,RNA_pol_Rpb2_6,	RNA polymerase beta subunit,RNA polymerase Rpb2, domain 2,RNA polymerase Rpb2, domain 7,RNA polymerase Rpb2, domain 3,RNA polymerase I, Rpa2 specific domain,RNA polymerase Rpb2, domain 6,	DNA-directed RNA polymerase
PDIG_37180	-1.99	-3.69				hypothetical protein PDIP_83770
PDIG_62380	-2.00	-3.87	PF11951,PF00172,	Fungal_trans_2,Zn_clus,	Fungal specific transcription factor domain,Fungal Zn(2)-Cys(6) binuclear cluster domain,	C6 sexual development transcription factor NosA
PDIG_74660	-2.02	-4.12	PF00005,PF00664,	ABC_tran,ABC_membrane,	ABC transporter,ABC transporter transmembrane region,	ABC transporter, putative
PDIG_51750	-2.04	-2.79				hypothetical protein PDIG_51750
PDIG_77390	-2.05	-2.64	PF07690,	MFS_1,	Major Facilitator Superfamily,	hypothetical protein PDIG_77390

PDIG_32410	-2.07	-4.51	PF00069,	Pkinase,	Protein kinase domain,	hypothetical protein PDIP_52000
PDIG_91250	-2.07	-2.00	PF00069,	Pkinase,	Protein kinase domain,	hypothetical protein PDIG_91250
PDIG_64810	-2.08	-2.07	PF02775,PF00205, PF02776,	TPP_enzyme_C,TPP _enzyme_M,TPP_en zyme_N,	Thiamine pyrophosphate enzyme, C-terminal TPP binding domain,Thiamine pyrophosphate enzyme, central domain,Thiamine pyrophosphate enzyme, N-terminal TPP binding domain,	Pyruvate decarboxylase, putative
PDIG_51850	-2.08	-2.70	PF00106,	adh_short,	short chain dehydrogenase,	hypothetical protein PDIG_51850
PDIG_12710	-2.09	-1.95	PF08442,PF16114,	ATP-grasp_2,Citrate _bind,	ATP-grasp domain,ATP citrate lyase citrate-binding,	ATP citrate lyase subunit (Acl), putatibe
PDIG_08970	-2.09	-3.11	PF00648,	Peptidase_C2,	Calpain family cysteine protease,	Calpain-like protein
PDIG_39970	-2.10	-3.10				hypothetical protein PDIG_39970
PDIG_54280	-2.12	-3.81	PF00501,PF00550, PF05141,PF02458,	AMP-binding,PP-bin ding,DIT1_PvcA,Tra nsferase,	AMP-binding enzyme,Phosphopantetheine attachment site,Pyoverdine/dityrosine biosynthesis protein,Transferase family,	Transferase family protein
PDIG_03140	-2.13	-3.71				hypothetical protein PDIP_41790
PDIG_47150	-2.15	-4.97	PF14223,PF07727, PF00665,	Retrotran_gag_2,RV T_2,rve,	gag-polypeptide of LTR copia-type,Reverse transcriptase (RNA-dependent DNA polymerase),Integrase core domain,	hypothetical protein PDIG_47150
PDIG_55390	-2.17	-1.85	PF01433,PF09127,	Peptidase_M1,Leuk-A4-hydro_C,	Peptidase family M1,Leukotriene A4 hydrolase, C-terminal,	Leukotriene A4 hydrolase
PDIG_09680	-2.19	-2.73	PF02560,	Cyanate_lyase,	Cyanate lyase C-terminal domain,	Cyanate hydratase

PDIG_06910	-2.19	-3.40	PF04488,	Gly_transf_sug,	Glycosyltransferase sugar-binding region containing DXD motif,	hypothetical protein PDIG_06910
PDIG_32430	-2.19	-4.64	PF00324,	AA_permease,	Amino acid permease,	hypothetical protein PDIP_52020
PDIG_90250	-2.20	-5.89	PF11838,PF01433,	ERAP1_C,Peptidase_M1,	ERAP1-like C-terminal domain,Peptidase family M1,	Aminopeptidase
PDIG_67650	-2.20	-3.73	PF00083,	Sugar_tr,	Sugar (and other) transporter,	hypothetical protein PDIP_76950
PDIG_45080	-2.21	-2.55	PF03972,	MmgE_PrpD,	MmgE/PrpD family,	2-methylcitrate dehydratase, putative
PDIG_42320	-2.21	-2.46	PF00732,PF05199,	GMC_oxred_N,GMC_oxred_C,	GMC oxidoreductase,GMC oxidoreductase,	hypothetical protein PDIG_42320
PDIG_70990	-2.21	-4.95	PF01490,	Aa_trans,	Transmembrane amino acid transporter protein,	hypothetical protein PDIP_80310
PDIG_86840	-2.22	-2.24	PF00067,	p450,	Cytochrome P450,	hypothetical protein PDIG_86840
PDIG_76080	-2.24	-2.33	PF00743,	FMO-like,	Flavin-binding monooxygenase-like,	Flavin-binding monooxygenase-like protein
PDIG_10600	-2.25	-2.45				Pc21g04140 [Penicillium chrysogenum Wisconsin 54-1255]
PDIG_02690	-2.27	-2.30	PF00324,	AA_permease,	Amino acid permease,	Amino acid permease, putative
PDIG_05020	-2.27	-1.80	PF03630,	Fumble,	Fumble,	Pantothenate kinase, putative
PDIG_48170	-2.28	-3.32	PF13450,	NAD_binding_8,	NAD(P)-binding Rossmann-like domain,	hypothetical protein PDIP_57550
PDIG_20360	-2.29	-4.36				hypothetical protein PDIG_20360
PDIG_63600	-2.29	-1.81				hypothetical protein PDIG_63600
PDIG_82190	-2.29	-1.75	PF02803,PF00108,	Thiolase_C,Thiolase_N,	Thiolase, C-terminal domain,Thiolase, N-terminal domain,	Pc22g10140 [Penicillium chrysogenum Wisconsin 54-1255]
PDIG_63230	-2.30	-2.29				hypothetical protein PDIP_72610

PDIG_55660	-2.32	-2.00				hypothetical protein PDIG_55660
PDIG_19810	-2.35	-2.78	PF00644,PF00179,	PARP,UQ_con,	Poly(ADP-ribose) polymerase catalytic domain,Ubiquitin-conjugating enzyme,	Ubiquitin conjugating enzyme, putative
PDIG_48690	-2.36	-2.04	PF07690,	MFS_1,	Major Facilitator Superfamily,	hypothetical protein PDIP_58070
PDIG_24580	-2.37	-2.11				hypothetical protein PDIP_59060
PDIG_87130	-2.39	-3.75	PF03098,PF00067,	An_peroxidase,p450,	Animal haem peroxidase,Cytochrome P450,	hypothetical protein PDIG_87130
PDIG_21480	-2.40	-3.17	PF00743,	FMO-like,	Flavin-binding monooxygenase-like,	Flavin-binding monooxygenase, putative
PDIG_80140	-2.40	-3.77	PF02668,	TauD,	Taurine catabolism dioxygenase TauD, TfdA family,	Pc13g10890 [Penicillium chrysogenum Wisconsin 54-1255]
PDIG_67330	-2.43	-2.18				hypothetical protein PDIP_76630
PDIG_50840	-2.46	-2.39	PF02548,	Pantoate_transf,	Ketopantoate hydroxymethyltransferase,	3-methyl-2-oxobutanoate hydroxymethyltransferase PanB
PDIG_45930	-2.49	-4.22				hypothetical protein PDIG_45930
PDIG_25360	-2.49	-6.06	PF00665,PF07727,	rve,RVT_2,	Integrase core domain,Reverse transcriptase (RNA-dependent DNA polymerase),	hypothetical protein PDIG_33240
PDIG_06440	-2.49	-6.06	PF00665,PF07727,	rve,RVT_2,	Integrase core domain,Reverse transcriptase (RNA-dependent DNA polymerase),	hypothetical protein PDIG_33240
PDIG_44570	-2.50	-3.56	PF00067,	p450,	Cytochrome P450,	hypothetical protein PDIG_44570
PDIG_76450	-2.51	-2.86	PF08240,PF00107,	ADH_N,ADH_zinc_N,	Alcohol dehydrogenase GroES-like domain,Zinc-binding dehydrogenase,	hypothetical protein PDIG_76450
PDIG_75570	-2.52	-3.46	PF00291,	PALP,	Pyridoxal-phosphate dependent enzyme,	Tryptophan synthase

PDIG_28880	-2.53	-4.11	PF00561,	Abhydrolase_1,	alpha/beta hydrolase fold,	hypothetical protein PDIP_63320
PDIG_70870	-2.59	-3.43				hypothetical protein PDIP_80190
PDIG_86690	-2.61	-3.24	PF00581,	Rhodanese,	Rhodanese-like domain,	hypothetical protein PDIG_86690
PDIG_26220	-2.62	-4.69				hypothetical protein PDIP_60700
PDIG_34790	-2.62	-2.59	PF13489,	Methyltransf_23,	Methyltransferase domain,	hypothetical protein PDIP_54360
PDIG_57930	-2.63	-6.04	PF00067,	p450,	Cytochrome P450,	Bifunctional P450:NADPH-P450 reductase
PDIG_43010	-2.67	-3.17				hypothetical protein PDIG_43010
PDIG_91260	-2.68	-4.14				hypothetical protein PDIG_91260
PDIG_33380	-2.69	-2.48	PF00501,PF13193,	AMP-binding,AMP-binding_C,	AMP-binding enzyme,AMP-binding enzyme C-terminal domain,	Phenylacetyl-CoA ligase
PDIG_43670	-2.70	-3.89	PF03184,PF03221,	DDE_1,HTH_Tnp_Tc5,	DDE superfamily endonuclease,Tc5 transposase DNA-binding domain,	Histone H3
PDIG_58080	-2.71	-3.73	PF03632,PF03636,	Glyco_hydro_65m,Glyco_hydro_65N,	Glycosyl hydrolase family 65 central catalytic domain,Glycosyl hydrolase family 65, N-terminal domain,	Alpha,alpha-trehalose glucohydrolase TreA/Ath1
PDIG_16190	-2.72	-4.90	PF02801,PF00106, PF00109,	Ketoacyl-synt_C,adh_short,ketoacyl-synt,	Beta-ketoacyl synthase, C-terminal domain,short chain dehydrogenase,Beta-ketoacyl synthase, N-terminal domain,	Fatty acid synthase subunit alpha, putative
PDIG_32750	-2.73	-6.85	PF11001,	DUF2841,	Protein of unknown function (DUF2841),	hypothetical protein PDIG_32750
PDIG_15450	-2.76	-2.85	PF16862,	Glyco_hydro_79C,	Glycosyl hydrolase family 79 C-terminal beta domain,	hypothetical protein PDIG_15450
PDIG_34570	-2.79	-6.15	PF02780,PF02779, PF00456,	Transketolase_C,Transket_pyr,Transketol	Transketolase, C-terminal domain,Transketolase, pyrimidine	hypothetical protein PDIG_34570

				ase_N,	binding domain,Transketolase, thiamine diphosphate binding domain,	
PDIG_03410	-2.88	-3.57	PF00069,	Pkinase,	Protein kinase domain,	hypothetical protein PDIG_03410
PDIG_22270	-2.89	-3.58	PF13424,PF13374,	TPR_12,TPR_10,	Tetratricopeptide repeat,Tetratricopeptide repeat,	hypothetical protein PDIG_22270
PDIG_81790	-2.89	-7.58	PF00152,	tRNA-synt_2,	tRNA synthetases class II (D, K and N),	hypothetical protein PDIG_81790
PDIG_70830	-2.91	-4.45	PF00067,	p450,	Cytochrome P450,	cytochrome P-450 14DM [Penicillium digitatum]
PDIG_86670	-2.96	-3.65	PF13176,	TPR_7,	Tetratricopeptide repeat,	hypothetical protein PDIG_86670
PDIG_24590	-2.98	-3.68				hypothetical protein PDIG_24590
PDIG_34560	-2.99	-6.31	PF17189,PF02055,	Glyco_hydro_30C,Glyco_hydro_30,	Glycosyl hydrolase family 30 beta sandwich domain,Glycosyl hydrolase family 30 TIM-barrel domain,	Beta-1,6-glucanase Neg1, putative
PDIG_21490	-3.00	-4.92	PF00891,	Methyltransf_2,	O-methyltransferase,	hypothetical protein PDIG_21490
PDIG_57830	-3.01	-4.58				hypothetical protein PDIP_67340
PDIG_39980	-3.03	-3.70	PF00106,	adh_short,	short chain dehydrogenase,	Short-chain dehydrogenase/reductase 2, putative
PDIG_24520	-3.07	-2.28	PF00394,PF07732, PF07731,	Cu-oxidase,Cu-oxidase_3,Cu-oxidase_2,	Multicopper oxidase,Multicopper oxidase,Multicopper oxidase,	Hypothetical protein PDIP_59000
PDIG_35840	-3.08	-3.12				hypothetical protein PDIG_35840
PDIG_16120	-3.11	-2.76	PF04479,	RTA1,	RTA1 like protein,	hypothetical protein PDIP_87630
PDIG_75350	-3.17	-2.84	PF01794,PF08030, PF08022,	Ferric_reduct,NAD_binding_6,FAD_binding_8,	Ferric reductase like transmembrane component,Ferric reductase NAD binding domain,FAD-binding domain,	NADPH oxidase (NoxA), putative
PDIG_04070	-3.18	-4.10				hypothetical protein PDIG_04070

PDIG_00120	-3.20	-8.12	PF08240,	ADH_N,	Alcohol dehydrogenase GroES-like domain,	NADP-dependent alcohol dehydrogenase [Grosmannia clavigera kw1407]
PDIG_75660	-3.22	-3.94	PF00734,PF00331,	CBM_1,Glyco_hydro_10,	Fungal cellulose binding domain,Glycosyl hydrolase family 10,	Endo-1,4-beta-xylanase, putative
PDIG_32460	-3.23	-3.22	PF03184,	DDE_1,	DDE superfamily endonuclease,	hypothetical protein PDIG_32460
PDIG_91270	-3.25	-5.20				hypothetical protein PDIG_91270
PDIG_67240	-3.25	-3.63	PF14021,	DUF4237,	Protein of unknown function (DUF4237),	hypothetical protein PDIP_76540
PDIG_81760	-3.27	-6.98				hypothetical protein PDIG_81760
PDIG_32950	-3.30	-3.85	PF08491,	SE,	Squalene epoxidase,	Squalene epoxidase-like protein
PDIG_25300	-3.34	-2.16				hypothetical protein PDIP_59790
PDIG_16160	-3.34	-3.62	PF00067,	p450,	Cytochrome P450,	Benzoate 4-monooxygenase cytochrome P450
PDIG_31620	-3.35	-3.43	PF07690,	MFS_1,	Major Facilitator Superfamily,	hypothetical protein PDE_09880 [Penicillium oxalicum 114-2]
PDIG_52750	-3.49	-2.95	PF00753,	Lactamase_B,	Metallo-beta-lactamase superfamily,	Metallo-beta-lactamase superfamily protein
PDIG_83640	-3.51	-4.70	PF07690,	MFS_1,	Major Facilitator Superfamily,	MFS monocarboxylate transporter, putative
PDIG_56310	-3.52	-3.35	PF02775,PF02776, PF00205,	TPP_enzyme_C,TPP_enzyme_N,TPP_enzyme_M,	Thiamine pyrophosphate enzyme, C-terminal TPP binding domain,Thiamine pyrophosphate enzyme, N-terminal TPP binding domain,Thiamine pyrophosphate enzyme, central domain,	hypothetical protein PDIP_65870

PDIG_68830	-3.54	-3.78	PF12311,	DUF3632,	Protein of unknown function (DUF3632),	hypothetical protein PDIP_78110
PDIG_78050	-3.60	-3.68	PF01498,PF13358,	HTH_Tnp_Tc3_2,DDE_3,	Transposase,DDE superfamily endonuclease,	Transposable element tc3 transposase, putative
PDIG_77400	-3.61	-3.33	PF07727,PF00665,	RVT_2,rve,	Reverse transcriptase (RNA-dependent DNA polymerase),Integrase core domain,	Retrovirus-related Pol polyprotein from transposon TNT 1-94
PDIG_45840	-3.68	-3.73	PF01370,	Epimerase,	NAD dependent epimerase/dehydratase family,	hypothetical protein PDIG_45840
PDIG_73390	-3.89	-4.13	PF07690,	MFS_1,	Major Facilitator Superfamily,	MFS multidrug resistance transporter, putative
PDIG_70500	-3.96	-5.31	PF01494,	FAD_binding_3,	FAD binding domain,	hypothetical protein PDIP_79810
PDIG_02680	-3.99	-2.17				2-haloalkanoic acid dehalogenase, putative
PDIG_33180	-4.01	-4.12	PF04116,	FA_hydroxylase,	Fatty acid hydroxylase superfamily,	hypothetical protein PDIP_52770
PDIG_70460	-4.02	-3.37	PF00875,PF03441,	DNA_photolyase,FAD_binding_7,	DNA photolyase,FAD binding domain of DNA photolyase,	DNA photolyase, putative
PDIG_76380	-4.17	-7.02				hypothetical protein PDIG_76380
PDIG_83570	-4.18	-6.23	PF07690,	MFS_1,	Major Facilitator Superfamily,	MFS multidrug transporter, putative
PDIG_45330	-4.27	-3.72				hypothetical protein PDIG_45330
PDIG_02350	-4.41	-6.45	PF00005,PF01061, PF06422,	ABC_tran,ABC2_membrane,PDR_CDR,	ABC transporter,ABC-2 type transporter,CDR ABC transporter,	ABC transporter, putative
PDIG_51840	-4.43	-5.98				Integral membrane protein Pth11-like, putative
PDIG_16180	-4.47	-3.97	PF01063,	Aminotran_4,	Amino-transferase class IV,	Branched-chain amino acid aminotransferase

PDIG_66050	-4.73	-7.73				hypothetical protein PDIP_75350
PDIG_68750	-4.90	-2.50	PF03663,	Glyco_hydro_76,	Glycosyl hydrolase family 76,	Glycosyl hydrolase, putative
PDIG_42030	-4.93	-5.52	PF01828,	Peptidase_A4,	Peptidase A4 family,	Aspergillopepsin, putative
PDIG_01990	-5.13	-7.04				hypothetical protein PDIG_01990
PDIG_66060	-5.48	-9.28				hypothetical protein PDIG_66060
PDIG_64840	-5.49	-4.50	PF08540,PF01154,	HMG_CoA_synt_C, HMG_CoA_synt_N,	Hydroxymethylglutaryl-coenzyme A synthase C terminal,Hydroxymethylglutaryl-coenzyme A synthase N terminal,	hypothetical protein PDIP_74170
PDIG_18140	-6.83	-6.52	PF00075,PF14529, PF00078,	RNase_H,Exo_endo_phos_2,RVT_1,	RNase H,Endonuclease-reverse transcriptase,Reverse transcriptase (RNA-dependent DNA polymerase),	putative RNA-directed DNA polymerase from transposon X-element
PDIG_15540	-6.86	-7.22	PF13673,	Acetyltransf_10,	Acetyltransferase (GNAT) domain,	Polyamine acetyltransferase
PDIG_16300	-7.43	-7.12	PF00665,	rve,	Integrase core domain,	hypothetical protein PDIG_33240
PDIG_11580	-8.79	-8.48	PF00665,PF08284, PF00078,PF00098,	rve,RVP_2,RVT_1,zf-CCHC,	Integrase core domain,Retroviral aspartyl protease,Reverse transcriptase (RNA-dependent DNA polymerase),Zinc knuckle,	Retrotransposon polyprotein, putative
98 common elements in "△PdsreA-up" and "△PdsreB-up":						
PDIG_22810	8.28	8.49	PF00135,	COesterase,	Carboxylesterase family,	Triacylglycerol lipase (LipA), putative
PDIG_84400	7.58	2.65	PF00324,	AA_permease,	Amino acid permease,	hypothetical protein PDIG_84400
PDIG_54620	7.43	8.52	PF03328,	HpcH_HpaI,	HpcH/HpaI aldolase/citrate lyase family,	2,4-dihydroxyhept-2-ene-1,7-dioic acid aldolase, putative

PDIG_54640	7.34	8.27	PF03171,PF14226,	2OG-FeII_Oxy,DIOX_N,	2OG-Fe(II) oxygenase superfamily,non-haem dioxygenase in morphine synthesis N-terminal,	Gibberellin 3-beta hydroxylase, putative
PDIG_54610	7.25	5.93	PF07690,	MFS_1,	Major Facilitator Superfamily,	hypothetical protein PDIG_54610
PDIG_00580	7.20	4.92	PF05199,PF00732,	GMC_oxred_C,GMC_oxred_N,	GMC oxidoreductase,GMC oxidoreductase,	Glucose oxidase, putative
PDIG_65980	7.05	4.45	PF00665,PF07727,	rve,RVT_2,	Integrase core domain,Reverse transcriptase (RNA-dependent DNA polymerase),	hypothetical protein PDIG_33240
PDIG_58160	6.93	3.50	PF00251,PF08244,	Glyco_hydro_32N,Glyco_hydro_32C,	Glycosyl hydrolases family 32 N-terminal domain,Glycosyl hydrolases family 32 C terminal,	Extracellular invertase
PDIG_54630	6.55	8.09	PF01042,	Ribonuc_L-PSP,	Endoribonuclease L-PSP,	hypothetical protein PDIG_54630
PDIG_46550	6.25	3.79	PF02826,PF00389,	2-Hacid_dh_C,2-Hacid_dh,	D-isomer specific 2-hydroxyacid dehydrogenase, NAD binding domain,D-isomer specific 2-hydroxyacid dehydrogenase, catalytic domain,	Formate dehydrogenase
PDIG_27790	5.11	2.55				hypothetical protein PDIP_62230
PDIG_35500	4.99	4.28	PF06628,PF00199,	Catalase-rel,Catalase,	Catalase-related immune-responsive,Catalase,	Catalase
PDIG_87520	4.88	1.96				hypothetical protein PDIG_87520
PDIG_88040	4.70	3.33	PF03211,	Pectate_lyase,	Pectate lyase,	putative pectate lyase 1 [Penicillium digitatum]
PDIG_57950	4.63	4.91	PF08450,	SGL,	SMP-30/Gluconolaconase/LRE-like region,	hypothetical protein PDIP_67450

PDIG_17140	4.50	3.25	PF02668,	TauD,	Taurine catabolism dioxygenase TauD, TfdA family,	hypothetical protein PDIP_55050
PDIG_48410	4.43	4.69	PF00544,	Pec_lyase_C,	Pectate lyase,	putative pectin lyase 2 [Penicillium digitatum]
PDIG_28100	4.43	2.38	PF00295,	Glyco_hydro_28,	Glycosyl hydrolases family 28,	putative exopolygalacturonase 1 [Penicillium digitatum]
PDIG_03520	4.35	2.35	PF07690,	MFS_1,	Major Facilitator Superfamily,	hypothetical protein PDIG_03520
PDIG_91100	4.22	3.91	PF00710,	Asparaginase,	Asparaginase, N-terminal,	L-asparaginase
PDIG_00830	4.13	3.56	PF00083,	Sugar_tr,	Sugar (and other) transporter,	MFS monosaccharide transporter, putative
PDIG_50400	4.09	3.54	PF00962,	A_deaminase,	Adenosine/AMP deaminase,	Adenosine deaminase
PDIG_54650	3.84	3.95	PF13561,	adh_short_C2,	Enoyl-(Acyl carrier protein) reductase,	3-ketoacyl-acyl carrier protein reductase, putative
PDIG_36660	3.81	3.88	PF00291,	PALP,	Pyridoxal-phosphate dependent enzyme,	1-aminocyclopropane-1-carboxylate deaminase, putative [Penicillium digitatum Pd1]
PDIG_50100	3.78	3.10	PF02728,PF01179, PF02727,	Cu_amine_oxidN3,Cu_amine_oxid,Cu_amine_oxidN2,	Copper amine oxidase, N3 domain,Copper amine oxidase, enzyme domain,Copper amine oxidase, N2 domain,	Primary amine oxidase
PDIG_51500	3.77	2.97				hypothetical protein PDIG_51500
PDIG_36540	3.74	3.14	PF06516,	NUP,	Purine nucleoside permease (NUP),	Purine nucleoside permease, putative
PDIG_36090	3.72	1.87	PF01301,PF13364, PF13363,PF10435,	Glyco_hydro_35,BetaGal_dom4_5,BetaGal_dom3,BetaGal_do	Glycosyl hydrolases family 35,Beta-galactosidase jelly roll domain,Beta-galactosidase, domain	hypothetical protein PDIG_36090

				m2,	3,Beta-galactosidase, domain 2,	
PDIG_05350	3.68	2.77	PF01095,	Pectinesterase,	Pectinesterase,	Pectin methylesterase, putative
PDIG_51490	3.61	4.04				hypothetical protein PDIG_51490
PDIG_71910	3.55	2.05	PF01384,	PHO4,	Phosphate transporter family,	Sodium/phosphate symporter, putative
PDIG_82760	3.13	4.40	PF07690,	MFS_1,	Major Facilitator Superfamily,	Pc22g09270 [Penicillium chrysogenum Wisconsin 54-1255]
PDIG_01900	3.06	1.73	PF13537,PF00733,	GATase_7,Asn_synthase,	Glutamine amidotransferase domain,Asparagine synthase,	Asparagine synthetase Asn2, putative
PDIG_51150	3.05	4.00				Glycosyl hydrolase, putative
PDIG_90800	3.04	3.13	PF05199,PF00732,	GMC_oxred_C,GMC_oxred_N,	GMC oxidoreductase,GMC oxidoreductase,	Cellobiose dehydrogenase
PDIG_08170	3.03	4.78	PF01619,	Pro_dh,	Proline dehydrogenase,	Proline oxidase PrnD
PDIG_51800	3.02	4.61	PF01670,	Glyco_hydro_12,	Glycosyl hydrolase family 12,	hypothetical protein PDIG_51800
PDIG_30080	2.95	2.69	PF00295,PF13460,	Glyco_hydro_28,NAD_binding_10,	Glycosyl hydrolases family 28,NAD(P)H-binding,	RecName: Full=Polygalacturonase; Short=PG; AltName: Full=Pectinase; Flags: Precursor
PDIG_08180	2.80	3.08	PF00324,	AA_permease,	Amino acid permease,	Proline-specific permease
PDIG_36670	2.79	2.33	PF00107,PF08240,	ADH_zinc_N,ADH_N,	Zinc-binding dehydrogenase,Alcohol dehydrogenase GroES-like domain,	Formaldehyde dehydrogenase
PDIG_85320	2.72	2.21	PF00860,	Xan_ur_permease,	Permease family,	Purine permease, putative
PDIG_88160	2.55	2.80	PF07690,	MFS_1,	Major Facilitator Superfamily,	Pantothenate transporter
PDIG_60720	2.53	2.03	PF01266,	DAO,	FAD dependent oxidoreductase,	hypothetical protein PDIP_70150
PDIG_65660	2.27	2.39	PF00701,	DHDPS,	Dihydrodipicolinate synthetase family,	Dihydrodipicolinate synthetase family protein

PDIG_81110	2.26	1.78	PF14226,	DIOX_N,	non-haem dioxygenase in morphine synthesis N-terminal,	2OG-Fe(II) oxygenase superfamily protein [Aspergillus kawachii IFO 4308]
PDIG_57310	2.20	2.72	PF11937,PF11693,	DUF3455,DUF2990,	Protein of unknown function (DUF3455),Protein of unknown function (DUF2990),	putative fungal protein
PDIG_02310	2.20	1.79	PF02812,PF00208,	ELFV_dehydrog_N, ELFV_dehydrog,	Glu/Leu/Phe/Val dehydrogenase, dimerisation domain,Glutamate/Leucine/Phenylalanine/Valine dehydrogenase,	NADP-specific glutamate dehydrogenase
PDIG_26810	2.20	1.85	PF00487,	FA_desaturase,	Fatty acid desaturase,	Oleate delta-12 desaturase
PDIG_85730	2.17	2.21	PF04828,	GFA,	Glutathione-dependent formaldehyde-activating enzyme,	Putative glutathione-dependent formaldehyde-activating enzyme
PDIG_06950	2.15	2.18	PF03820,	Mtc,	Tricarboxylate carrier,	Mitochondrial cation transporter, putative
PDIG_33430	2.12	3.08	PF02727,PF01179, PF02728,	Cu_amine_oxidN2,Cu_amine_oxid,Cu_amine_oxidN3,	Copper amine oxidase, N2 domain,Copper amine oxidase, enzyme domain,Copper amine oxidase, N3 domain,	Primary amine oxidase
PDIG_87460	2.11	2.76	PF01490,	Aa_trans,	Transmembrane amino acid transporter protein,	Neutral amino acid permease
PDIG_47800	2.07	2.31	PF05199,PF00732,	GMC_oxred_C,GMC_oxred_N,	GMC oxidoreductase,GMC oxidoreductase,	hypothetical protein PDIP_57180
PDIG_05470	2.06	1.92				Naphthalene 1,2-dioxygenase subunit alpha
PDIG_58210	2.05	2.26	PF01979,	Amidohydro_1,	Amidohydrolase family,	Chlorohydrolase family protein

PDIG_23250	2.05	2.58				Pc13g06590 [Penicillium chrysogenum Wisconsin 54-1255]
PDIG_81860	2.04	3.03	PF03561,	Allantoicase,	Allantoicase repeat,	hypothetical protein PDIG_81860
PDIG_76480	1.99	3.07	PF00840,PF00734,	Glyco_hydro_7,CBM_1,	Glycosyl hydrolase family 7,Fungal cellulose binding domain,	1,4-beta-D-glucan-cellobiohydrolyase, putative
PDIG_71560	1.98	2.97	PF12796,	Ank_2,	Ankyrin repeats (3 copies),	hypothetical protein PDIP_80860
PDIG_88030	1.95	1.67	PF13410,PF13409,	GST_C_2,GST_N_2,	Glutathione S-transferase, C-terminal domain,Glutathione S-transferase, N-terminal domain,	hypothetical protein PDIG_88030
PDIG_04480	1.94	2.18	PF03959,	FSH1,	Serine hydrolase (FSH1),	Dihydrofolate reductase
PDIG_42380	1.94	2.63	PF17186,	Lipocalin_9,	Lipocalin-like domain,	hypothetical protein PDIG_42380
PDIG_20080	1.88	2.46	PF01253,	SUI1,	Translation initiation factor SUI1,	Pc20g07470 [Penicillium chrysogenum Wisconsin 54-1255]
PDIG_29700	1.87	4.03				hypothetical protein PDIG_29700
PDIG_91230	1.80	3.44	PF05704,	Caps_synth,	Capsular polysaccharide synthesis protein,	hypothetical protein PDIG_91230
PDIG_35270	1.76	1.97	PF00756,	Esterase,	Putative esterase,	Esterase, putative
PDIG_23090	1.75	2.01	PF13902,	R3H-assoc,	R3H-associated N-terminal domain,	hypothetical protein PDIG_23090
PDIG_47730	1.74	5.83				putative membrane protein
PDIG_47500	1.74	2.20	PF13848,PF00085,	Thioredoxin_6,Thioredoxin,	Thioredoxin-like domain,Thioredoxin,	Protein disulfide isomerase
PDIG_44860	1.73	2.27				IgE binding protein, putative
PDIG_38960	1.69	2.55				hypothetical protein PDIG_38960
PDIG_31400	1.59	1.82	PF13561,	adh_short_C2,	Enoyl-(Acyl carrier protein) reductase,	Carbonyl reductase, putative
PDIG_32650	1.59	2.33	PF01680,	SOR_SNZ,	SOR/SNZ family,	Pyridoxine biosynthesis protein pyroA

PDIG_87610	1.58	2.10	PF00817,PF11799, PF00200,PF13688,	IMS,IMS_C,Disintegrin,Reprolysin_5,	impB/mucB/samB family,impB/mucB/samB family C-terminal domain,Disintegrin,Metallo-peptidase family M12,	hypothetical protein PDIG_87610
PDIG_22890	1.56	1.66				hypothetical protein PDIG_22890
PDIG_39430	1.55	1.69	PF13302,	Acetyltransf_3,	Acetyltransferase (GNAT) domain,	Acetyltransferase
PDIG_90070	1.54	2.33				Acetyltransferase, GNAT family
PDIG_53390	1.53	1.87	PF08881,	CVNH,	CVNH domain,	hypothetical protein PDIG_53390
PDIG_65670	1.50	1.86	PF01408,	GFO_IDH_MocA,	Oxidoreductase family, NAD-binding Rossmann fold,	hypothetical protein PDIP_74990
PDIG_03380	1.47	1.65	PF00544,	Pec_lyase_C,	Pectate lyase,	PL1 [Penicillium digitatum]
PDIG_09590	1.47	2.35	PF00179,	UQ_con,	Ubiquitin-conjugating enzyme,	Ubiquitin conjugating enzyme (UbcF), putative
PDIG_82530	1.46	1.90	PF08636,	Pkr1,	ER protein Pkr1,	ER membrane protein (Pkr1), putative
PDIG_26870	1.45	2.16	PF08534,	Redoxin,	Redoxin,	Allergen, putative
PDIG_89020	1.45	2.23	PF07047,	OPA3,	Optic atrophy 3 protein (OPA3),	hypothetical protein PDIG_89020
PDIG_52870	1.44	1.61	PF00291,	PALP,	Pyridoxal-phosphate dependent enzyme,	Cysteine synthase
PDIG_20600	1.40	1.84				hypothetical protein PDIG_20600
PDIG_81880	1.39	1.70	PF07859,	Abhydrolase_3,	alpha/beta hydrolase fold,	Lipase/esterase, putative
PDIG_62090	1.37	1.99	PF13489,	Methyltransf_23,	Methyltransferase domain,	S-adenosylmethionine-dependent methyltransferase, putative
PDIG_76470	1.31	1.67	PF00551,PF13740,	Formyl_trans_N,ACT_6,	Formyl transferase,ACT domain,	Formyltetrahydrofolate deformylase, putative

PDIG_67250	1.28	1.73	PF13302,	Acetyltransf_3,	Acetyltransferase (GNAT) domain,	Acetyltransferase, GNAT family
PDIG_67880	1.23	3.22				hypothetical protein PDIP_77180
PDIG_15430	1.22	1.97	PF03061,PF01583,	4HBT,APS_kinase,	Thioesterase superfamily,Adenylylsulphate kinase,	Adenylyl-sulfate kinase [Penicillium digitatum Pd1]
PDIG_58330	1.21	2.08	PF07716,	bZIP_2,	Basic region leucine zipper,	bZIP transcription factor (HacA), putative [Neosartorya fischeri NRRL 181]
PDIG_34930	1.20	1.98				hypothetical protein PDIP_54490
PDIG_50390	1.18	1.72	PF01300,PF03481,	Sua5_yciO_yrdC,SUA5,	Telomere recombination,Putative GTP-binding controlling metal-binding,	Translation initiation protein Sua5
PDIG_88120	1.12	1.74	PF16653,PF03435,	Sacchrp_dh_C,Sacchrp_dh_NADP,	Saccharopine dehydrogenase C-terminal domain,Saccharopine dehydrogenase NADP binding domain,	saccharopine reductase [Penicillium chrysogenum]
PDIG_09380	1.12	5.77	PF01786,	AOX,	Alternative oxidase,	Alternative oxidase
PDIG_68240	1.10	2.61	PF00235,	Profilin,	Profilin,	Profilin
