# Supplementary material for: Functional analysis of two sterol regulatory element binding proteins in Penicillium digitatum
Source: PLoS One. 2017 May 3;12(5):e0176485. doi: 10.1371/journal.pone.0176485 (PMC5415137; doi:10.1371/journal.pone.0176485)
Supplement: S6 Table — (PDF) [file pone.0176485.s010.pdf]

S6 Table. Annotation of genes uniquely regulated by *PdsreA*

ID	log2FC in △PdsreA	Pfam family	domain name(abbreviation)	domain name	nr_annotation
133 elements included exclusively in "△PdsreA-down":					
PDIG_63570	-1.10	PF07690,	MFS_1,	Major Facilitator Superfamily,	Pc18g02960 [Penicillium chrysogenum Wisconsin 54-1255]
PDIG_71250	-1.10	PF00575,	S1,	S1 RNA binding domain,	RRNA biogenesis protein RRP5, putative
PDIG_35230	-1.10				hypothetical protein PDIP_54780
PDIG_30110	-1.13	PF00428,	Ribosomal_60s,	60s Acidic ribosomal protein,	60S acidic ribosomal protein P2
PDIG_71520	-1.13	PF00583,	Acetyltransf_1,	Acetyltransferase (GNAT) family,	hypothetical protein PDIP_80820
PDIG_84040	-1.14	PF00743,PF04082,	FMO-like,Fungal_trans,	Flavin-binding monooxygenase-like,Fungal specific transcription factor domain,	Fungal specific transcription factor, putative
PDIG_68480	-1.15	PF00636,PF03368,	Ribonuclease_3,Dicer_dimer,	Ribonuclease III domain,Dicer dimerisation domain,	Dicer-like protein 2
PDIG_84710	-1.15	PF16198,PF01472,PF01509,PF04511,PF08068,	TruB_C_2,PUA,TruB_N,DER1,DKCLD,	tRNA pseudouridylate synthase B C-terminal domain,PUA domain,TruB family pseudouridylate synthase (N terminal domain),Der1-like family,DKCLD (NUC011) domain,	Centromere/microtubule-binding protein CBF5
PDIG_88130	-1.15	PF08327,PF09229,	AHSA1,Aha1_N,	Activator of Hsp90 ATPase homolog 1-like protein,Activator of Hsp90 ATPase, N-terminal,	Pc22g05790 [Penicillium chrysogenum Wisconsin 54-1255]
PDIG_64000	-1.16	PF05178,PF12936,	Kri1,Kri1_C,	KRI1-like family,KRI1-like family C-terminal,	Ribosome biogenesis protein Kri1
PDIG_15640	-1.18	PF11951,	Fungal_trans_2,	Fungal specific transcription factor domain,	hypothetical protein PDIG_15640
PDIG_07260	-1.18	PF00043,PF02798,	GST_C,GST_N,	Glutathione S-transferase, C-terminal domain,Glutathione S-transferase, N-terminal	Translation elongation factor eEF-1B gamma subunit, putative

				domain,	
PDIG_89420	-1.19	PF08238,	Sel1,	Sel1 repeat,	hypothetical protein PDIG_89420
PDIG_12570	-1.19	PF03949,PF00390,	Malic_M,malic,	Malic enzyme, NAD binding domain,Malic enzyme, N-terminal domain,	malate dehydrogenase (oxaloacetate-decarboxylating) [Exophiala dermatitidis NIH/UT8656]
PDIG_84990	-1.20				Protein kinase
PDIG_57320	-1.21	PF01269,	Fibrillarin,	Fibrillarin,	Fibrillarin, putative
PDIG_78490	-1.21	PF04588,	HIG_1_N,	Hypoxia induced protein conserved region,	hypothetical protein PDIG_78490
PDIG_02290	-1.21	PF08241,	Methyltransf_11,	Methyltransferase domain,	hypothetical protein PDIG_02290
PDIG_56300	-1.22	PF13561,	adh_short_C2,	Enoyl-(Acyl carrier protein) reductase,	hypothetical protein PDIP_65860
PDIG_39900	-1.23				Mitochondrial distribution and morphology protein 34
PDIG_13640	-1.24	PF17125,PF01189,	Methyltr_RsmF_N, Methyltr_RsmB-F,	N-terminal domain of 16S rRNA methyltransferase RsmF,16S rRNA methyltransferase RsmB/F,	Nucleolar RNA methyltransferase (Nop2), putative
PDIG_91030	-1.25	PF00160,	Pro_isomerase,	Cyclophilin type peptidyl-prolyl cis-trans isomerase/CLD,	Peptidyl-prolyl cis-trans isomerase D
PDIG_70480	-1.26	PF02780,PF02779,	Transketolase_C,Transket_pyr,	Transketolase, C-terminal domain,Transketolase, pyrimidine binding domain,	3-methyl-2-oxobutanoate dehydrogenase, putative
PDIG_20950	-1.27	PF00645,	zf-PARP,	Poly(ADP-ribose) polymerase and DNA-Ligase Zn-finger region,	PARP-type zinc finger-containing protein C2A9.07c
PDIG_18740	-1.27				hypothetical protein PDIP_56570
PDIG_73920	-1.27	PF00400,	WD40,	WD domain, G-beta repeat,	Pc22g03140 [Penicillium chrysogenum Wisconsin 54-1255]
PDIG_54410	-1.28	PF00650,PF03765,	CRAL_TRIO,CRAL_TRIO_N,	CRAL/TRIO domain,CRAL/TRIO, N-terminal domain,	Phosphatidylinositol transporter, putative
PDIG_47330	-1.28	PF00583,	Acetyltransf_1,	Acetyltransferase (GNAT) family,	Acetyltransferase, GNAT family

PDIG_12760	-1.29	PF00166,	Cpn10,	Chaperonin 10 Kd subunit,	Chaperonin, putative
PDIG_04330	-1.29	PF01965,	DJ-1_PfpI,	DJ-1/PfpI family,	ThiJ/PfpI family protein
PDIG_31790	-1.30	PF01039,	Carboxyl_trans,	Carboxyl transferase domain,	3-methylcrotonyl-CoA carboxylase, beta subunit (MccB), putative
PDIG_39040	-1.30	PF13409,PF13410,	GST_N_2,GST_C_2,	Glutathione S-transferase, N-terminal domain,Glutathione S-transferase, C-terminal domain,	hypothetical protein PDIG_39040
PDIG_56530	-1.30	PF01207,	Dus,	Dihydrouridine synthase (Dus),	tRNA dihydrouridine synthase, putative
PDIG_77380	-1.32	PF13561,	adh_short_C2,	Enoyl-(Acyl carrier protein) reductase,	Sorbitol/xylulose reductase Sou1-like, putative
PDIG_47770	-1.33				hypothetical protein PDIG_47770
PDIG_17540	-1.33	PF10277,	Frag1,	Frag1/DRAM/Sfk1 family,	FK506 suppressor Sfk1, putative
PDIG_76930	-1.33	PF11951,	Fungal_trans_2,	Fungal specific transcription factor domain,	hypothetical protein PDIG_76930
PDIG_40060	-1.34	PF00266,	Aminotran_5,	Aminotransferase class-V,	Kynureninase
PDIG_03890	-1.34	PF00561,	Abhydrolase_1,	alpha/beta hydrolase fold,	Pc20g03890 [Penicillium chrysogenum Wisconsin 54-1255]
PDIG_22360	-1.34	PF00004,	AAA,	ATPase family associated with various cellular activities (AAA),	AAA family ATPase, putative
PDIG_08090	-1.38	PF00676,	E1_dh,	Dehydrogenase E1 component,	2-oxoisovalerate dehydrogenase complex alpha subunit, putative
PDIG_43630	-1.39	PF00107,	ADH_zinc_N,	Zinc-binding dehydrogenase,	Alcohol dehydrogenase, putative
PDIG_64350	-1.39				hypothetical protein PDIP_73690
PDIG_87000	-1.39	PF05712,PF11717,	MRG,Tudor-knot,	MRG,RNA binding activity-knot of a chromodomain,	Histone acetylase complex subunit MRG15-2
PDIG_64130	-1.40	PF00172,	Zn_clus,	Fungal Zn(2)-Cys(6) binuclear cluster domain,	hypothetical protein PDIP_73480
PDIG_56750	-1.41	PF00400,	WD40,	WD domain, G-beta repeat,	WD repeat protein
PDIG_20110	-1.41				hypothetical protein PDIG_20110

PDIG_55550	-1.41	PF13434,	K_oxygenase,	L-lysine 6-monooxygenase (NADPH-requiring),	L-ornithine N5 monooxygenase
PDIG_22490	-1.42	PF02782,PF00370,	FGGY_C,FGGY_N,	FGGY family of carbohydrate kinases, C-terminal domain,FGGY family of carbohydrate kinases, N-terminal domain,	FGGY-family carbohydrate kinase, putative
PDIG_55470	-1.42	PF00291,PF00571,	PALP,CBS,	Pyridoxal-phosphate dependent enzyme,CBS domain,	Cystathionine beta-synthase, putative
PDIG_11700	-1.42				hypothetical protein PDIG_11700
PDIG_61640	-1.43	PF01246,	Ribosomal_L24e,	Ribosomal protein L24e,	hypothetical protein PDIP_71070
PDIG_63620	-1.43	PF01474,	DAHP_synth_2,	Class-II DAHP synthetase family,	Phospho-2-dehydro-3-deoxyheptonate aldolase, class II
PDIG_66810	-1.43	PF01798,PF08156,	Nop,NOP5NT,	snoRNA binding domain, fibrillarin,NOP5NT (NUC127) domain,	Pre-rRNA processing nucleolar protein Sik1, putative
PDIG_48180	-1.44				hypothetical protein PDIP_57560
PDIG_55000	-1.44				NADH-ubiquinone oxidoreductase 9.5 kDa subunit, putative
PDIG_83990	-1.45	PF08297,	U3_snoRNA_assoc,	U3 snoRNA associated,	hypothetical protein PDIP_89480
PDIG_61240	-1.46				hypothetical protein PDIG_61240
PDIG_75870	-1.46				hypothetical protein PDIG_75870
PDIG_59730	-1.48	PF04427,	Brix,	Brix domain,	hypothetical protein PDIP_69150
PDIG_41170	-1.48	PF00364,PF02817,PF00198,	Biotin_lipoyl,E3_binding,2-oxoacid_dh,	Biotin-requiring enzyme,e3 binding domain,2-oxoacid dehydrogenases acyltransferase (catalytic domain),	Biotin-dependent 2-oxo acid dehydrogenases acyltransferase, putative
PDIG_11360	-1.48	PF11274,	DUF3074,	Protein of unknown function (DUF3074),	hypothetical protein PDIP_82860
PDIG_27920	-1.49	PF00463,	ICL,	Isocitrate lyase family,	Isocitrate lyase
PDIG_03200	-1.49	PF00625,	Guanylate_kin,	Guanylate kinase,	Guanylate kinase

PDIG_36830	-1.50	PF10329,	DUF2417,	Region of unknown function (DUF2417),	Mitochondrial integral membrane protein
PDIG_24330	-1.51				hypothetical protein PDIP_58810
PDIG_75310	-1.52	PF01172,	SBDS,	Shwachman-Bodian-Diamond syndrome (SBDS) protein,	RNA binding protein, putative
PDIG_66090	-1.54				hypothetical protein PDIP_75390
PDIG_04810	-1.54	PF08883,	DOPA_dioxygen,	Dopa 4,5-dioxygenase family,	hypothetical protein PDIG_04810
PDIG_07850	-1.55				hypothetical protein PDIP_35890
PDIG_23700	-1.56	PF00288,PF08544,	GHMP_kinases_N, GHMP_kinases_C,	GHMP kinases N terminal domain,GHMP kinases C terminal,	Mevalonate kinase
PDIG_58300	-1.58	PF01144,	CoA_trans,	Coenzyme A transferase,	succinyl-CoA:3-ketoacid-coenzyme A transferase [Neosartorya fischeri NRRL 181]
PDIG_23460	-1.59	PF00725,PF02737,	3HCDH,3HCDH_N,	3-hydroxyacyl-CoA dehydrogenase, C-terminal domain,3-hydroxyacyl-CoA dehydrogenase, NAD binding domain,	3-hydroxybutyryl-CoA dehydrogenase, putative
PDIG_41570	-1.60				hypothetical protein PDIG_41570
PDIG_72980	-1.60	PF00153,	Mito_carr,	Mitochondrial carrier protein,	Mitochondrial carrier protein, putative
PDIG_22380	-1.63				hypothetical protein PDIG_22380
PDIG_56320	-1.64				hypothetical protein PDIP_65880
PDIG_78890	-1.64	PF00549,PF00755,	Ligase_CoA,Carn_acyltransf,	CoA-ligase,Choline/Carnitine o-acyltransferase,	Carnitine acetyl transferase
PDIG_53050	-1.65				hypothetical protein PDIG_53050
PDIG_29290	-1.66	PF00378,	ECH_1,	Enoyl-CoA hydratase/isomerase,	Enoyl-CoA hydratase/isomerase family protein
PDIG_44310	-1.67	PF00205,PF02775,	TPP_enzyme_M,TPP_enzyme_C,	Thiamine pyrophosphate enzyme, central domain,Thiamine pyrophosphate enzyme, C-terminal TPP binding domain,	acetolactate synthase [Aspergillus oryzae RIB40]
PDIG_55650	-1.67				hypothetical protein PDIG_55650

PDIG_31800	-1.67	PF02771,PF00441,PF02770,	Acyl-CoA_dh_N,Acyl-CoA_dh_1,Acyl-CoA_dh_M,	Acyl-CoA dehydrogenase, N-terminal domain,Acyl-CoA dehydrogenase, C-terminal domain,Acyl-CoA dehydrogenase, middle domain,	Isovaleryl-CoA dehydrogenase IvdA, putative
PDIG_08310	-1.70	PF04209,	HgmA,	homogentisate 1,2-dioxygenase,	Homogentisate 1,2-dioxygenase (HmgA), putative
PDIG_31050	-1.72	PF01161,	PBP,	Phosphatidylethanolamine-binding protein,	Protease inhibitor (Tfs1), putative
PDIG_56920	-1.73	PF00081,PF02777,	Sod_Fe_N,Sod_Fe_C,	Iron/manganese superoxide dismutases, alpha-hairpin domain,Iron/manganese superoxide dismutases, C-terminal domain,	Superoxide dismutase
PDIG_08300	-1.74	PF01557,PF09298,	FAA_hydrolase,FAA_hydrolase_N,	Fumarylacetoacetate (FAA) hydrolase family,Fumarylacetoacetase N-terminal,	Fumarylacetoacetate hydrolase FahA
PDIG_70470	-1.74	PF05368,	NmrA,	NmrA-like family,	Isoflavone reductase, putative
PDIG_07390	-1.78				hypothetical protein PDIG_07390
PDIG_71010	-1.79	PF01556,PF00226,PF00684,	DnaJ_C,DnaJ,DnaJ_CXXCXGXG,	DnaJ C terminal domain,DnaJ domain,DnaJ central domain,	hypothetical protein PDIP_80330
PDIG_08080	-1.80				hypothetical protein PDIG_45050
PDIG_45050	-1.80	PF14529,PF00078,PF00075,	Exo_endo_phos_2,RVT_1,RNase_H,	Endonuclease-reverse transcriptase,Reverse transcriptase (RNA-dependent DNA polymerase),RNase H,	hypothetical protein PDIG_45050
PDIG_24510	-1.81	PF03239,	FTR1,	Iron permease FTR1 family,	Plasma membrane iron permease
PDIG_57140	-1.81	PF13489,	Methyltransf_23,	Methyltransferase domain,	hypothetical protein PDIP_66680
PDIG_12190	-1.82				hypothetical protein PDIP_38410
PDIG_78090	-1.85	PF04427,	Brix,	Brix domain,	Imp4 protein, putative
PDIG_65470	-1.86	PF13236,PF13374,PF1280	CLU,TPR_10,eIF3_p135,TPR_12,CL	Clustered mitochondria,Tetratricopeptide repeat,Translation initiation factor eIF3 subunit	hypothetical protein PDIP_74790

		7,PF13424,PF15044,	U_N,	135,Tetratricopeptide repeat,Mitochondrial function, CLU-N-term,	
PDIG_77450	-1.88	PF11700,	ATG22,	Vacuole effluxer Atg22 like,	hypothetical protein PDIG_77450
PDIG_38170	-1.88	PF13418,	Kelch_4,	Galactose oxidase, central domain,	Kelch repeat protein
PDIG_02770	-1.89	PF00108,PF02803,	Thiolase_N,Thiolase_C,	Thiolase, N-terminal domain,Thiolase, C-terminal domain,	Sterol carrier protein, putative
PDIG_45340	-1.91	PF13561,PF07247,	adh_short_C2,AATase,	Enoyl-(Acyl carrier protein) reductase,Alcohol acetyltransferase,	hypothetical protein PDIP_17280 [Penicillium digitatum Pd1]
PDIG_83000	-1.93	PF07081,	DUF1349,	Protein of unknown function (DUF1349),	hypothetical protein PDIP_86790
PDIG_60520	-1.99				hypothetical protein PDIP_69930
PDIG_27670	-1.99	PF00067,	p450,	Cytochrome P450,	Cytochrome P450 monooxygenase, putative
PDIG_03260	-1.99	PF00403,	HMA,	Heavy-metal-associated domain,	hypothetical protein PDIP_41920
PDIG_32310	-2.02				hypothetical protein PDIP_51900
PDIG_51810	-2.06	PF04622,	ERG2_Sigma1R,	ERG2 and Sigma1 receptor like protein,	C-8 sterol isomerase (Erg-1), putative
PDIG_75680	-2.07				hypothetical protein PDIG_75680
PDIG_20140	-2.12	PF08547,	CIA30,	Complex I intermediate-associated protein 30 (CIA30),	hypothetical protein PDIG_20140
PDIG_07340	-2.16	PF14683,PF14686,	CBM-like,fn3_3,	Polysaccharide lyase family 4, domain III,Polysaccharide lyase family 4, domain II,	hypothetical protein PDIG_07340
PDIG_00260	-2.18				hypothetical protein PDIG_00260
PDIG_57550	-2.20				hypothetical protein PDIP_67060
PDIG_25230	-2.21				hypothetical protein PDIP_59720
PDIG_56950	-2.23	PF01425,PF00106,	Amidase,adh_short,	Amidase,short chain dehydrogenase,	General amidase, putative
PDIG_53070	-2.26	PF03328,	HpcH_HpaI,	HpcH/HpaI aldolase/citrate lyase family,	2,4-dihydroxyhept-2-ene-1,7-dioic acid aldolase

PDIG_83260	-2.27	PF05063,	MT-A70,	MT-A70,	MT-A70 family
PDIG_42220	-2.32				hypothetical protein PDIG_42220
PDIG_29970	-2.36	PF08538,	DUF1749,	Protein of unknown function (DUF1749),	Siderophore biosynthesis lipase/esterase, putative
PDIG_84380	-2.36	PF08570,	DUF1761,	Protein of unknown function (DUF1761),	hypothetical protein PDIG_84380
PDIG_69650	-2.37				hypothetical protein PDIP_78940
PDIG_62940	-2.38				hypothetical protein PDIP_72320
PDIG_18400	-2.42	PF07992,PF00355,	Pyr_redox_2,Rieske,	Pyridine nucleotide-disulphide oxidoreductase,Rieske [2Fe-2S] domain,	AIF-like mitochondrial oxidoreductase (Nfrl), putative
PDIG_13300	-2.43	PF14033,	DUF4246,	Protein of unknown function (DUF4246),	hypothetical protein PDIP_40440
PDIG_38270	-2.56				hypothetical protein PDIG_38270
PDIG_24500	-2.58	PF01794,PF08022,PF08030,	Ferric_reduct,FAD_binding_8,NAD_binding_6,	Ferric reductase like transmembrane component,FAD-binding domain,Ferric reductase NAD binding domain,	Ferric reductase transmembrane component 5, putative
PDIG_38260	-2.59	PF07690,	MFS_1,	Major Facilitator Superfamily,	siderochrome-iron transporter (Sit1), putative [Aspergillus clavatus NRRL 1]
PDIG_53850	-2.73				hypothetical protein PDIG_53850
PDIG_46910	-3.19	PF04116,	FA_hydroxylase,	Fatty acid hydroxylase superfamily,	Sterol delta 5,6-desaturase ERG3
PDIG_22330	-3.21	PF04116,	FA_hydroxylase,	Fatty acid hydroxylase superfamily,	C-4 methyl sterol oxidase Erg25, putative
PDIG_77480	-3.31	PF00172,	Zn_clus,	Fungal Zn(2)-Cys(6) binuclear cluster domain,	hypothetical protein PDIG_77480
PDIG_90350	-3.67	PF01284,	MARVEL,	Membrane-associating domain,	Non-classical export protein, putative
PDIG_71130	-3.68				hypothetical protein PDIG_71130
PDIG_90150	-9.40	PF00010,PF09427,	HLH,DUF2014,	Helix-loop-helix DNA-binding domain,Domain of unknown function (DUF2014),	HLH transcription factor, putative
259 elements included exclusively in "△PdsreA-up":					
PDIG_03030	6.30	PF11951,	Fungal_trans_2,	Fungal specific transcription factor domain,	hypothetical protein PDIG_15640

PDIG_87530	4.36				hypothetical protein PDIG_87530
PDIG_58090	4.10	PF00199,PF06628,	Catalase,Catalase-rel,	Catalase,Catalase-related immune-responsive,	Catalase B
PDIG_33090	4.09	PF14310,PF00933,PF01915,	Fn3-like,Glyco_hydro_3,Glyco_hydro_3_C,	Fibronectin type III-like domain,Glycosyl hydrolase family 3 N terminal domain,Glycosyl hydrolase family 3 C-terminal domain,	putative beta-glucosidase G
PDIG_60730	3.78	PF00171,	Aldedh,	Aldehyde dehydrogenase family,	hypothetical protein PDIP_70160
PDIG_59450	3.47	PF07745,	Glyco_hydro_53,	Glycosyl hydrolase family 53,	Arabinogalactan endo-1,4-beta-galactosidase GalA
PDIG_64230	3.28				hypothetical protein PDIP_73570
PDIG_87780	3.24				hypothetical protein PDIG_87780
PDIG_49820	3.15				hypothetical protein PDIG_49820
PDIG_44800	3.01	PF00083,	Sugar_tr,	Sugar (and other) transporter,	MFS monosaccharide transporter, putative
PDIG_25750	2.97				GABA permease [Aspergillus fumigatus Af293]
PDIG_14830	2.93	PF01095,	Pectinesterase,	Pectinesterase,	hypothetical protein PDIG_14830
PDIG_60760	2.91	PF01619,	Pro_dh,	Proline dehydrogenase,	Pyrroline-5-carboxylate reductase
PDIG_13580	2.90	PF03442,PF00150,	CBM_X2,Cellulase,	Carbohydrate binding domain X2,Cellulase (glycosyl hydrolase family 5),	Extracellular endoglucanase, putative
PDIG_13830	2.88				hypothetical protein PDIP_39480
PDIG_37780	2.84	PF00324,	AA_permease,	Amino acid permease,	Amino acid permease (Dip5), putative
PDIG_84890	2.83				hypothetical protein PDIG_84890
PDIG_87750	2.69	PF01212,	Beta_elim_lyase,	Beta-eliminating lyase,	Threonine aldolase, putative
PDIG_44930	2.62	PF07519,	Tannase,	Tannase and feruloyl esterase,	hypothetical protein PDIG_44930
PDIG_32690	2.58	PF03330,	DPBB_1,	Lytic transglycolase,	Expansin-like protein 1
PDIG_26800	2.57	PF06912,	DUF1275,	Protein of unknown function (DUF1275),	hypothetical protein PDIP_61250

PDIG_81120	2.54	PF02133,	Transp_cyt_pur,	Permease for cytosine/purines, uracil, thiamine, allantoin,	hypothetical protein PDIG_81120
PDIG_45070	2.53	PF00295,	Glyco_hydro_28,	Glycosyl hydrolases family 28,	Polygalacturonase-2
PDIG_03860	2.52	PF04488,	Gly_transf_sug,	Glycosyltransferase sugar-binding region containing DXD motif,	hypothetical protein PDIG_03860
PDIG_60740	2.47				hypothetical protein PDIP_70170
PDIG_81130	2.43	PF02133,	Transp_cyt_pur,	Permease for cytosine/purines, uracil, thiamine, allantoin,	hypothetical protein PDIG_81130
PDIG_49470	2.40	PF00282,	Pyridoxal_deC,	Pyridoxal-dependent decarboxylase conserved domain,	hypothetical protein PDIG_49470
PDIG_13650	2.40	PF00443,PF13920,PF02493,	UCH,zf-C3HC4_3,MORN,	Ubiquitin carboxyl-terminal hydrolase,Zinc finger, C3HC4 type (RING finger),MORN repeat,	putative MATH and UCH domain protein
PDIG_51040	2.40	PF16815,	HRI1,	Protein HRI1,	hypothetical protein PDIG_51040
PDIG_34250	2.38	PF06422,PF00005,PF14510,PF01061,	PDR_CDR,ABC_tran,ABC_trans_N,ABC2_membrane,	CDR ABC transporter,ABC transporter,ABC-transporter extracellular N-terminal,ABC-2 type transporter,	ABC transporter, putative
PDIG_17100	2.37	PF00083,	Sugar_tr,	Sugar (and other) transporter,	MFS monosaccharide transporter, putative
PDIG_67790	2.35	PF13520,	AA_permease_2,	Amino acid permease,	hypothetical protein PDIP_77100
PDIG_50110	2.31	PF00857,	Isochorismatase,	Isochorismatase family,	Isochorismatase family protein
PDIG_89860	2.28	PF13520,	AA_permease_2,	Amino acid permease,	hypothetical protein PDIG_89860
PDIG_68670	2.28	PF00232,	Glyco_hydro_1,	Glycosyl hydrolase family 1,	Beta-glucosidase
PDIG_15120	2.26				hypothetical protein PDIG_15120
PDIG_54580	2.24				hypothetical protein PDIG_54580
PDIG_31500	2.21	PF09362,	DUF1996,	Domain of unknown function (DUF1996),	hypothetical protein PDIP_51080 [Penicillium digitatum Pd1]

PDIG_83190	2.20	PF00152,PF01336,	tRNA-synt_2,tRNA_anti-codon,	tRNA synthetases class II (D, K and N),OB-fold nucleic acid binding domain,	Cytoplasmic asparaginyl-tRNA synthetase, putative
PDIG_00570	2.19	PF10282,	Lactonase,	Lactonase, 7-bladed beta-propeller,	hypothetical protein PDIG_00570
PDIG_15490	2.19	PF08669,PF01571,	GCV_T_C,GCV_T,	Glycine cleavage T-protein C-terminal barrel domain,Aminomethyltransferase folate-binding domain,	Aminomethyltransferase
PDIG_29560	2.11	PF04371,	PAD_porph,	Porphyromonas-type peptidyl-arginine deiminase,	hypothetical protein PDIP_63960
PDIG_42070	2.11				hypothetical protein UCREL1_8844 [Eutypa lata UCREL1]
PDIG_39440	2.11	PF00202,	Aminotran_3,	Aminotransferase class-III,	hypothetical protein PDIG_39440
PDIG_66580	2.10				hypothetical protein PDIP_75880
PDIG_46690	2.10	PF08244,PF00251,	Glyco_hydro_32C,Glyco_hydro_32N,	Glycosyl hydrolases family 32 C terminal,Glycosyl hydrolases family 32 N-terminal domain,	Putative intracellular invertase
PDIG_04910	2.10	PF07690,	MFS_1,	Major Facilitator Superfamily,	MFS transporter, putative
PDIG_71680	2.08				Integral membrane protein
PDIG_15350	2.08	PF00083,	Sugar_tr,	Sugar (and other) transporter,	hypothetical protein PDIG_15350
PDIG_24110	2.07	PF02776,PF00205,PF02775,	TPP_enzyme_N,TPP_enzyme_M,TPP_enzyme_C,	Thiamine pyrophosphate enzyme, N-terminal TPP binding domain,Thiamine pyrophosphate enzyme, central domain,Thiamine pyrophosphate enzyme, C-terminal TPP binding domain,	Ankyrin, putative
PDIG_36640	2.04	PF01658,PF07994,	Inos-1-P_synth,NAD_binding_5,	Myo-inositol-1-phosphate synthase,Myo-inositol-1-phosphate synthase,	hypothetical protein PDIP_89710
PDIG_52220	2.00	PF04082,	Fungal_trans,	Fungal specific transcription factor domain,	hypothetical protein PDIG_52220
PDIG_48560	2.00	PF00860,	Xan_ur_permease,	Permease family,	Nucleoside transporter, putative
PDIG_20170	1.97	PF00172,	Zn_clus,	Fungal Zn(2)-Cys(6) binuclear cluster domain,	hypothetical protein PDIG_20170
PDIG_33200	1.95	PF01137,	RTC,	RNA 3'-terminal phosphate cyclase,	RNA 3'-terminal phosphate cyclase,

					putative
PDIG_58220	1.95	PF02347,	GDC-P,	Glycine cleavage system P-protein,	Glycine dehydrogenase
PDIG_36840	1.94	PF00171,	Aldedh,	Aldehyde dehydrogenase family,	Aldehyde dehydrogenase
PDIG_58830	1.92	PF01764,	Lipase_3,	Lipase (class 3),	Extracellular triacylglycerol lipase, putative
PDIG_79190	1.89	PF13302,	Acetyltransf_3,	Acetyltransferase (GNAT) domain,	N-acetyltransferase, GNAT family, putative
PDIG_75190	1.88	PF13561,	adh_short_C2,	Enoyl-(Acyl carrier protein) reductase,	hypothetical protein PDIG_75190
PDIG_36960	1.88				hypothetical protein PDIP_83550
PDIG_89390	1.87				putative beta-glucosidase btgE
PDIG_61970	1.85	PF00580,PF13361,	UvrD-helicase,UvrD_C,	UvrD/REP helicase N-terminal domain,UvrD-like helicase C-terminal domain,	ATP-dependend DNA helicase, putative
PDIG_84160	1.85				hypothetical protein PDIG_84160
PDIG_33750	1.84	PF05730,	CFEM,	CFEM domain,	Extracellular serine-threonine rich protein
PDIG_43030	1.84	PF09334,	tRNA-synt_1g,	tRNA synthetases class I (M),	Methionyl-tRNA synthetase
PDIG_61420	1.83	PF00248,	Aldo_ket_red,	Aldo/keto reductase family,	Aldehyde reductase (GliO), putative
PDIG_34550	1.82				hypothetical protein PDIP_54110
PDIG_43660	1.82	PF00291,PF00290,	PALP,Trp_syntA,	Pyridoxal-phosphate dependent enzyme,Tryptophan synthase alpha chain,	Bifunctional tryptophan synthase TRPB
PDIG_53550	1.81	PF00668,PF00501,PF07993,PF00550,	Condensation,AMP-binding,NAD_binding_4,PP-binding,	Condensation domain,AMP-binding enzyme,Male sterility protein,Phosphopantetheine attachment site,	HC-toxin synthetase
PDIG_48990	1.80	PF08029,PF01634,	HisG_C,HisG,	HisG, C-terminal domain,ATP phosphoribosyltransferase,	hypothetical protein PDIP_58370
PDIG_49390	1.80				hypothetical protein PDIG_49390
PDIG_38810	1.80	PF00078,PF00075,PF14529,	RVT_1,RNase_H,Exo_endo_phos_2,	Reverse transcriptase (RNA-dependent DNA polymerase),RNase H,Endonuclease-reverse transcriptase,	putative RNA-directed DNA polymerase from transposon X-element

PDIG_83720	1.80				hypothetical protein PDIP_89220
PDIG_30830	1.80	PF00150,PF00734,	Cellulase,CBM_1,	Cellulase (glycosyl hydrolase family 5),Fungal cellulose binding domain,	putative endoglucanase/cellulase 1 [Penicillium digitatum]
PDIG_23080	1.79	PF04389,	Peptidase_M28,	Peptidase family M28,	Aminopeptidase, putative
PDIG_10930	1.79	PF03124,	EXS,	EXS family,	Protein-ER retention protein (Erd1), putative
PDIG_56600	1.78				hypothetical protein PDIP_66160
PDIG_87700	1.77	PF00501,PF07993,PF00550,	AMP-binding,NAD_binding_4,PP-binding,	AMP-binding enzyme,Male sterility protein,Phosphopantetheine attachment site,	L-aminoadipate-semialdehyde dehydrogenase
PDIG_58110	1.76	PF01699,	Na_Ca_ex,	Sodium/calcium exchanger protein,	hypothetical protein PDIP_67600
PDIG_06660	1.76	PF00328,	His_Phos_2,	Histidine phosphatase superfamily (branch 2),	Acid phosphatase, putative
PDIG_23790	1.75	PF02436,PF00289,PF02786,PF00364,PF00682,PF02785,	PYC_OADA,Biotin_carb_N,CPSase_L_D2,Biotin_lipoyl,HMGL-like,Biotin_carb_C,	Conserved carboxylase domain,Biotin carboxylase, N-terminal domain,Carbamoyl-phosphate synthase L chain, ATP binding domain,Biotin-requiring enzyme,HMGL-like,Biotin carboxylase C-terminal domain,	Pyruvate carboxylase, putative
PDIG_48400	1.74	PF01425,	Amidase,	Amidase,	Amidase family protein
PDIG_29590	1.74	PF13520,	AA_permease_2,	Amino acid permease,	Amino acid permease
PDIG_76170	1.73	PF07690,	MFS_1,	Major Facilitator Superfamily,	MFS multidrug transporter, putative
PDIG_72690	1.73	PF01988,	VIT1,	VIT family,	Calcium transporter, putative
PDIG_36170	1.73	PF00445,	Ribonuclease_T2,	Ribonuclease T2 family,	Ribonuclease T2, putative
PDIG_00200	1.72	PF13520,	AA_permease_2,	Amino acid permease,	Choline transporter Hnm1, putative
PDIG_30210	1.72	PF02786,PF02787,	CPSase_L_D2,CPSase_L_D3,	Carbamoyl-phosphate synthase L chain, ATP binding domain,Carbamoyl-phosphate synthetase large chain, oligomerisation domain,	Carbamoyl-phosphate synthase, large subunit
PDIG_00100	1.72				hypothetical protein PDIG_00100

PDIG_84670	1.70	PF13358,PF01498,	DDE_3,HTH_Tnp_Tc3_2,	DDE superfamily endonuclease,Transposase,	Transposable element tc3 transposase, putative
PDIG_87680	1.70	PF00941,PF03450,PF00111,PF01799,PF01315,PF02738,	FAD_binding_5,CO_deh_flav_C,Fer2,Fer2_2,Ald_Xan_dh_C,Ald_Xan_dh_C2,	FAD binding domain in molybdopterin dehydrogenase,CO dehydrogenase flavoprotein C-terminal domain,2Fe-2S iron-sulfur cluster binding domain,[2Fe-2S] binding domain,Aldehyde oxidase and xanthine dehydrogenase, a/b hammerhead domain,Molybdopterin-binding domain of aldehyde dehydrogenase,	Xanthine dehydrogenase HxA, putative
PDIG_38950	1.69	PF00464,	SHMT,	Serine hydroxymethyltransferase,	Serine hydroxymethyltransferase
PDIG_37200	1.69	PF04193,	PQ-loop,	PQ loop repeat,	L-cystine transporter, putative
PDIG_25890	1.66	PF00328,	His_Phos_2,	Histidine phosphatase superfamily (branch 2),	hypothetical protein PDIP_60370
PDIG_52230	1.66				hypothetical protein PDIG_52230
PDIG_42900	1.66				hypothetical protein PDIG_42900
PDIG_00610	1.64				hypothetical protein PDIG_00610
PDIG_32580	1.63	PF03987,PF03986,PF10381,	Autophagy_act_C,Autophagy_N,Autophagy_C,	Autophagocytosis associated protein, active-site domain,Autophagocytosis associated protein (Atg3), N-terminal domain,Autophagocytosis associated protein C-terminal,	Autophagy-related protein 3
PDIG_29740	1.63	PF04139,	Rad9,	Rad9,	DNA repair protein rad9, putative
PDIG_32620	1.61	PF01960,	ArgJ,	ArgJ family,	Arginine biosynthesis bifunctional protein ArgJ, mitochondrial
PDIG_03870	1.60	PF07690,	MFS_1,	Major Facilitator Superfamily,	Amine transporter, putative
PDIG_14050	1.60				hypothetical protein PDIP_39700
PDIG_01380	1.59				hypothetical protein PDIG_01380
PDIG_34980	1.58	PF03856,	SUN,	Beta-glucosidase (SUN family),	hypothetical protein PDIP_54540

PDIG_60710	1.57	PF04082,	Fungal_trans,	Fungal specific transcription factor domain,	hypothetical protein PDIP_70140
PDIG_84130	1.55				hypothetical protein PDIG_84130
PDIG_59840	1.54				hypothetical protein PDIP_69260
PDIG_82710	1.54	PF07983,PF03198,	X8,Glyco_hydro_72,	X8 domain,Glucanosyltransferase,	1,3-beta-glucanosyltransferase gel4
PDIG_46700	1.53	PF00083,	Sugar_tr,	Sugar (and other) transporter,	hypothetical protein PDIG_46700
PDIG_51830	1.53	PF07690,	MFS_1,	Major Facilitator Superfamily,	hypothetical protein PDIG_51830
PDIG_85440	1.52	PF05699,	Dimer_Tnp_hAT,	hAT family C-terminal dimerisation region,	hypothetical protein PDIG_86160
PDIG_86160	1.52	PF05699,	Dimer_Tnp_hAT,	hAT family C-terminal dimerisation region,	hypothetical protein PDIG_86160
PDIG_43040	1.51	PF09811,	Yae1_N,	Essential protein Yae1, N terminal,	hypothetical protein PDIG_43040
PDIG_76200	1.50	PF01490,	Aa_trans,	Transmembrane amino acid transporter protein,	hypothetical protein PDIG_76200
PDIG_81100	1.50				hypothetical protein PDIG_81100
PDIG_48000	1.49	PF13816,	Dehydratase_hem,	Haem-containing dehydratase,	hypothetical protein PDIP_57380
PDIG_62410	1.48	PF00083,	Sugar_tr,	Sugar (and other) transporter,	hypothetical protein PDIG_62410
PDIG_57670	1.47	PF03856,	SUN,	Beta-glucosidase (SUN family),	SUN family protein, putative
PDIG_29110	1.47	PF04488,	Gly_transf_sug,	Glycosyltransferase sugar-binding region containing DXD motif,	SNARE complex subunit (Vam7)
PDIG_81560	1.47				hypothetical protein PDIG_81560
PDIG_72760	1.47				hypothetical protein PDIG_72760
PDIG_54660	1.47	PF13813,	MBOAT_2,	Membrane bound O-acyl transferase family,	hypothetical protein PDIG_54660
PDIG_17220	1.46	PF00328,	His_Phos_2,	Histidine phosphatase superfamily (branch 2),	hypothetical protein PDIP_55120
PDIG_19570	1.46	PF01501,	Glyco_transf_8,	Glycosyl transferase family 8,	hypothetical protein PDIG_19570
PDIG_87790	1.45	PF00724,	Oxidored_FMN,	NADH:flavin oxidoreductase / NADH oxidase family,	12-oxophytodienoate reductase, putative
PDIG_44840	1.44	PF00120,	Gln-synt_C,	Glutamine synthetase, catalytic domain,	hypothetical protein PDE_04219 [Penicillium oxalicum 114-2]

PDIG_09300	1.44	PF07690,	MFS_1,	Major Facilitator Superfamily,	Efflux pump antibiotic resistance protein, putative
PDIG_83180	1.44	PF00076,PF10378,	RRM_1,RRM,	RNA recognition motif. (a.k.a. RRM, RBD, or RNP domain),Putative RRM domain,	Differentiation regulator (Nrd1), putative
PDIG_25990	1.43	PF02115,	Rho_GDI,	RHO protein GDP dissociation inhibitor,	hypothetical protein PDIG_25990
PDIG_45710	1.43	PF00005,PF06422,PF14510,PF01061,	ABC_tran,PDR_CDR,ABC_trans_N, ABC2_membrane,	ABC transporter,CDR ABC transporter,ABC-transporter extracellular N-terminal,ABC-2 type transporter,	ABC drug exporter AtrF
PDIG_48040	1.42	PF00995,	Sec1,	Sec1 family,	Sec1 family superfamily
PDIG_68550	1.42	PF01975,	SurE,	Survival protein SurE,	hypothetical protein PDIP_77840
PDIG_18030	1.42	PF01704,	UDPGP,	UTP--glucose-1-phosphate uridylyltransferase,	UTP-glucose-1-phosphate uridylyltransferase Ugp1, putative
PDIG_66590	1.41	PF01979,	Amidohydro_1,	Amidohydrolase family,	Allantoinase Dal1, putative
PDIG_63100	1.41	PF00141,	peroxidase,	Peroxidase,	Cytochrome c peroxidase Ccp1, putative
PDIG_24730	1.40	PF04909,	Amidohydro_2,	Amidohydrolase,	2-amino-3-carboxymuconate-6-semialdehyde decarboxylase, putative
PDIG_41940	1.40	PF00162,	PGK,	Phosphoglycerate kinase,	Phosphoglycerate kinase
PDIG_33290	1.39	PF11735,	CAP59_mtransfer,	Cryptococcal mannosyltransferase 1,	hypothetical protein PDIP_52870
PDIG_65920	1.39	PF00910,	RNA_helicase,	RNA helicase,	Para-Rep C2
PDIG_70000	1.38	PF00320,	GATA,	GATA zinc finger,	GATA transcription factor
PDIG_16670	1.38	PF00005,PF00664,	ABC_tran,ABC_membrane,	ABC transporter,ABC transporter transmembrane region,	hypothetical protein PDIP_88180
PDIG_37590	1.37	PF00326,	Peptidase_S9,	Prolyl oligopeptidase family,	Dipeptidyl-peptidase 5
PDIG_32180	1.37	PF00735,	Septin,	Septin,	Septin AspA, putative
PDIG_77560	1.37	PF03169,	OPT,	OPT oligopeptide transporter protein,	hypothetical protein PDIG_77560
PDIG_73820	1.36	PF04061,	ORMDL,	ORMDL family,	Unfolded protein response Orm1, putative

PDIG_66140	1.36	PF00025,	Arf,	ADP-ribosylation factor family,	ADP-ribosylation factor 6, putative
PDIG_10720	1.36	PF00009,PF03143,	GTP_EFTU,GTP_EFTU_D3,	Elongation factor Tu GTP binding domain,Elongation factor Tu C-terminal domain,	hypothetical protein PDIP_82230
PDIG_75470	1.35	PF02990,	EMP70,	Endomembrane protein 70,	Multispanning membrane protein, putative
PDIG_31170	1.35				hypothetical protein PDIP_50750
PDIG_13820	1.35				hypothetical protein PDIP_39470
PDIG_22520	1.35				hypothetical protein PDIG_22520
PDIG_48030	1.34	PF00271,PF12513,	Helicase_C,SUV3_C,	Helicase conserved C-terminal domain,Mitochondrial degradasome RNA helicase subunit C terminal,	Mitochondrial ATP-dependent RNA helicase Suv3, putative
PDIG_81970	1.34	PF03447,PF00742,	NAD_binding_3,Homoserine_dh,	Homoserine dehydrogenase, NAD binding domain,Homoserine dehydrogenase,	Homoserine dehydrogenase
PDIG_76610	1.33	PF00724,	Oxidored_FMN,	NADH:flavin oxidoreductase / NADH oxidase family,	NADH oxidase
PDIG_25020	1.33	PF00169,	PH,	PH domain,	hypothetical protein PDIG_25020
PDIG_90060	1.33	PF14698,PF00206,	ASL_C2,Lyase_1,	Argininosuccinate lyase C-terminal,Lyase,	Argininosuccinate lyase
PDIG_62770	1.32				hypothetical protein PDIP_72150
PDIG_03510	1.32	PF00795,	CN_hydrolase,	Carbon-nitrogen hydrolase,	Nitrilase, putative
PDIG_81980	1.31	PF11927,	DUF3445,	Protein of unknown function (DUF3445),	hypothetical protein PDIG_81980
PDIG_78350	1.31	PF14604,	SH3_9,	Variant SH3 domain,	hypothetical protein PDIG_78350
PDIG_21170	1.31	PF01263,	Aldose_epim,	Aldose 1-epimerase,	Aldose 1-epimerase, putative
PDIG_52240	1.31	PF05277,	DUF726,	Protein of unknown function (DUF726),	hypothetical protein PDIG_52240
PDIG_40960	1.31	PF07690,	MFS_1,	Major Facilitator Superfamily,	MFS transporter (Mch2), putative
PDIG_78430	1.31	PF13520,	AA_permease_2,	Amino acid permease,	GabA permease, putative
PDIG_25160	1.30				hypothetical protein PDIP_59650
PDIG_77890	1.30	PF12223,	DUF3602,	Protein of unknown function (DUF3602),	hypothetical protein PDIG_77890

PDIG_62540	1.29	PF00245,	Alk_phosphatase,	Alkaline phosphatase,	Alkaline phosphatase
PDIG_46330	1.27	PF03641,	Lysine_decarbox,	Possible lysine decarboxylase,	hypothetical protein PDIG_46330
PDIG_74520	1.27	PF09333,PF13329,	ATG_C,ATG2_CAD,	Autophagy-related protein C terminal domain,Autophagy-related protein 2 CAD motif,	Autophagy regulatory protein Atg2, putative
PDIG_70690	1.27	PF03928,	Haem_degrading,	Haem-degrading,	hypothetical protein PDIP_80010
PDIG_74530	1.27	PF00962,	A_deaminase,	Adenosine/AMP deaminase,	CECR1 family adenosine deaminase, putative
PDIG_40560	1.26	PF03169,	OPT,	OPT oligopeptide transporter protein,	Oligopeptide transporter, putative
PDIG_25030	1.26	PF00892,	EamA,	EamA-like transporter family,	Integral membrane protein, putative
PDIG_57890	1.26	PF00133,PF08264,	tRNA-synt_1,Anticodon_1,	tRNA synthetases class I (I, L, M and V),Anticodon-binding domain of tRNA,	hypothetical protein DACRYDRAFT_20729 [Dacryopinax sp. DJM-731 SS1]
PDIG_24940	1.25	PF03171,	2OG-FeII_Oxy,	2OG-Fe(II) oxygenase superfamily,	Oxidoreductase, 2OG-Fe(II) oxygenase family, putative
PDIG_18840	1.25	PF07217,	Het-C,	Heterokaryon incompatibility protein Het-C,	NIMA-interacting protein TinC
PDIG_44850	1.25	PF01274,	Malate_synthase,	Malate synthase,	Malate synthase
PDIG_27140	1.25	PF07690,	MFS_1,	Major Facilitator Superfamily,	MFS transporter, putative
PDIG_79150	1.25	PF07690,	MFS_1,	Major Facilitator Superfamily,	Protein HOL1, putative
PDIG_68500	1.24	PF00171,	Aldedh,	Aldehyde dehydrogenase family,	Aldehyde dehydrogenase, putative
PDIG_24560	1.24	PF07690,	MFS_1,	Major Facilitator Superfamily,	Pc21g07950 [Penicillium chrysogenum Wisconsin 54-1255]
PDIG_84660	1.24	PF01490,	Aa_trans,	Transmembrane amino acid transporter protein,	Transporter, putative
PDIG_10550	1.23	PF00026,	Asp,	Eukaryotic aspartyl protease,	Aspartic protease pep1
PDIG_64720	1.23	PF00503,	G-alpha,	G-protein alpha subunit,	G protein complex alpha subunit GanA
PDIG_66000	1.22				pH-response transcription factor pacC/RIM101
PDIG_58180	1.22	PF05577,	Peptidase_S28,	Serine carboxypeptidase S28,	Serine peptidase, family S28, putative
PDIG_59830	1.22	PF03950,PF00749,	tRNA-synt_1c_C,tRNA-synt_1c,	tRNA synthetases class I (E and Q), anti-codon binding domain,tRNA synthetases class I (E and Q),	Glutaminyl-tRNA synthetase

				catalytic domain,	
PDIG_04490	1.22				hypothetical protein PDIG_04490
PDIG_54050	1.22	PF00735,PF00400,	Septin,WD40,	Septin,WD domain, G-beta repeat,	hypothetical protein THITE_2108385 [Thielavia terrestris NRRL 8126]
PDIG_61960	1.22	PF03069,	FmdA_AmdA,	Acetamidase/Formamidase family,	Acetamidase/formamidase family protein
PDIG_53280	1.21	PF04502,	DUF572,	Family of unknown function (DUF572),	MRNA splicing protein Yju2
PDIG_79030	1.21				Ammonium transporter (Mep2), putative
PDIG_31160	1.21	PF00561,	Abhydrolase_1,	alpha/beta hydrolase fold,	Homoserine O-acetyltransferase, putative
PDIG_52420	1.21	PF00328,	His_Phos_2,	Histidine phosphatase superfamily (branch 2),	Phytase
PDIG_56670	1.21	PF07690,	MFS_1,	Major Facilitator Superfamily,	MFS allantoate transporter, putative
PDIG_09390	1.20				hypothetical protein PDIG_09390
PDIG_33490	1.20	PF04982,	HPP,	HPP family,	HPP family protein
PDIG_32110	1.20	PF00561,	Abhydrolase_1,	alpha/beta hydrolase fold,	hypothetical protein PDIP_51690
PDIG_44830	1.20	PF13561,	adh_short_C2,	Enoyl-(Acyl carrier protein) reductase,	Short-chain dehydrogenase/reductase family protein, putative
PDIG_68650	1.20	PF01266,	DAO,	FAD dependent oxidoreductase,	hypothetical protein PDIP_77940
PDIG_68180	1.19	PF04982,	HPP,	HPP family,	HPP family protein
PDIG_67510	1.19	PF01597,	GCV_H,	Glycine cleavage H-protein,	Glycine cleavage system H protein
PDIG_15680	1.19				hypothetical protein PDIG_15680
PDIG_43170	1.19	PF05572,	Peptidase_M43,	Pregnancy-associated plasma protein-A,	Extracellular metalloprotease [Penicillium digitatum Pd1]
PDIG_76030	1.19	PF00225,	Kinesin,	Kinesin motor domain,	Kinesin family protein (KinA), putative
PDIG_67780	1.19	PF09286,PF00082,	Pro-kuma_activ,Peptidase_S8,	Pro-kumamolisin, activation domain,Subtilase family,	Tripeptidyl-peptidase sed2
PDIG_03210	1.19	PF00735,	Septin,	Septin,	hypothetical protein PDIP_41870
PDIG_31760	1.19	PF07855,	DUF1649,	Protein of unknown function (DUF1649),	hypothetical protein AN2280.2 [Aspergillus

					nidulans FGSC A4]
PDIG_02540	1.18	PF03456,PF02141,PF03455,	uDENN,DENN,dDENN,	uDENN domain,DENN (AEX-3) domain,dDENN domain,	hypothetical protein PDIG_02540
PDIG_13280	1.18	PF08303,PF08302,PF09511,	tRNA_lig_kinase,tRNA_lig_CPD,RNA_lig_T4_1,	tRNA ligase kinase domain,Fungal tRNA ligase phosphodiesterase domain,RNA ligase,	TRNA ligase
PDIG_09960	1.18	PF00668,PF00550,PF00501,	Condensation,PP-binding,AMP-binding,	Condensation domain,Phosphopantetheine attachment site,AMP-binding enzyme,	HC-toxin synthetase
PDIG_24450	1.18	PF01008,	IF-2B,	Initiation factor 2 subunit family,	hypothetical protein PDIP_58930
PDIG_69380	1.18	PF13302,	Acetyltransf_3,	Acetyltransferase (GNAT) domain,	hypothetical protein PDIP_78670
PDIG_89580	1.18	PF04082,	Fungal_trans,	Fungal specific transcription factor domain,	C6 transcription factor, putative
PDIG_67970	1.18	PF00545,	Ribonuclease,	ribonuclease,	hypothetical protein PDIP_77270
PDIG_69240	1.16				hypothetical protein PDIP_78530
PDIG_04450	1.16	PF14823,PF14824,PF13241,PF00590,	Sirohm_synth_C,Sirohm_synth_M,NAD_binding_7,TP_methylase,	Sirohaem biosynthesis protein C-terminal,Sirohaem biosynthesis protein central,Putative NAD(P)-binding,Tetrapyrrole (Corrin/Porphyrin) Methylases,	Siroheme synthase, putative
PDIG_80280	1.15	PF09816,	EAF,	RNA polymerase II transcription elongation factor,	hypothetical protein PDIG_80280
PDIG_44730	1.15	PF14661,	HAUS6_N,	HAUS augmin-like complex subunit 6 N-terminus,	hypothetical protein PDIG_44730
PDIG_29320	1.15	PF01764,	Lipase_3,	Lipase (class 3),	Autophagy related lipase Atg15, putative
PDIG_14140	1.15	PF03198,	Glyco_hydro_72,	Glucanosyltransferase,	1,3-beta-glucanosyltransferase gel2
PDIG_22110	1.15	PF01450,PF07991,	IlvC,IlvN,	Acetohydroxy acid isomeroreductase, catalytic domain,Acetohydroxy acid isomeroreductase, NADPH-binding domain,	Ketol-acid reductoisomerase

PDIG_22850	1.14	PF00884,PF04987,	Sulfatase,PigN,	Sulfatase,Phosphatidylinositolglycan class N (PIG-N),	GPI ethanolamine phosphate transferase 1
PDIG_47340	1.14	PF04768,	NAT,	NAT, N-acetyltransferase, of N-acetylglutamate synthase,	Amino-acid acetyltransferase, mitochondrial
PDIG_58340	1.14	PF05291,	Bystin,	Bystin,	RRNA processing protein Bystin, putative
PDIG_35580	1.13	PF10415,PF00206,	FumaraseC_C,Lyase_1,	Fumarase C C-terminus,Lyase,	Fumarate hydratase
PDIG_17480	1.13	PF00854,	PTR2,	POT family,	MFS peptide transporter Ptr2, putative
PDIG_80900	1.13	PF10233,	Cg6151-P,	Uncharacterized conserved protein CG6151-P,	Clathrin-coated vesicle protein, putative
PDIG_85660	1.13	PF00884,	Sulfatase,	Sulfatase,	hypothetical protein PDIG_85660
PDIG_86490	1.12	PF03372,	Exo_endo_phos,	Endonuclease/Exonuclease/phosphatase family,	Sphingomyelinase family protein, putative
PDIG_78380	1.12	PF03169,	OPT,	OPT oligopeptide transporter protein,	Oligonucleotide transporter
PDIG_19560	1.12	PF12859,	ANAPC1,	Anaphase-promoting complex subunit 1,	20S cyclosome subunit (APC1/BimE), putative
PDIG_33890	1.12	PF01406,	tRNA-synt_1e,	tRNA synthetases class I (C) catalytic domain,	Cysteinyl-tRNA synthetase
PDIG_17390	1.12	PF03676,	UPF0183,	Uncharacterised protein family (UPF0183),	hypothetical protein PDIP_55290
PDIG_89550	1.12	PF03876,PF00575,	SHS2_Rpb7-N,S1,	SHS2 domain found in N terminus of Rpb7p/Rpc25p/MJ0397,S1 RNA binding domain,	Pc12g03390 [Penicillium chrysogenum Wisconsin 54-1255]
PDIG_46580	1.12	PF01926,PF12631,PF10396,	MMR_HSR1,MnmE_helical,TrmE_N,	50S ribosome-binding GTPase,MnmE helical domain,GTP-binding protein TrmE N-terminus,	Mitochondrial GTPase (Mss1), putative
PDIG_30890	1.12	PF00202,	Aminotran_3,	Aminotransferase class-III,	4-aminobutyrate transaminase
PDIG_84170	1.11	PF00083,	Sugar_tr,	Sugar (and other) transporter,	MFS myo-inositol transporter, putative
PDIG_27090	1.11	PF05653,	Mg_trans_NIPA,	Magnesium transporter NIPA,	hypothetical protein PDIP_61530
PDIG_57150	1.11				hypothetical protein PDIP_66690
PDIG_76110	1.11	PF02872,PF0	5_nucleotid_C,Met	5'-nucleotidase, C-terminal domain,Calcineurin-like	5'-nucleotidase, putative

		0149,	allophos,	phosphoesterase,	
PDIG_39770	1.11				hypothetical protein PDIG_39770
PDIG_61880	1.11				Allergen Asp f 4
PDIG_34910	1.11	PF02265,	S1-P1_nuclease,	S1/P1 Nuclease,	Nuclease PA3, putative
PDIG_15690	1.10				hypothetical protein PDIG_15690
PDIG_63150	1.10	PF12063,PF0 0069,	DUF3543,Pkinase,	Domain of unknown function (DUF3543),Protein kinase domain,	Serine/threonine protein kinase (Pdd7p), putative
PDIG_69990	1.10	PF06813,	Nodulin-like,	Nodulin-like,	hypothetical protein PDIP_79290
PDIG_62340	1.09	PF08229,	SHR3_chaperone,	ER membrane protein SH3,	Secretory component protein shr3, putative
PDIG_59710	1.09	PF00677,	Lum_binding,	Lumazine binding domain,	Riboflavin synthase, alpha subunit
PDIG_06560	1.09	PF00171,	Aldedh,	Aldehyde dehydrogenase family,	Aldehyde dehydrogenase
PDIG_88650	1.09	PF10397,PF0 0206,	ADSL_C,Lyase_1,	Adenylosuccinate lyase C-terminus,Lyase,	Adenylosuccinate lyase Ade13, putative
PDIG_30980	1.09	PF00117,PF0 0988,	GATase,CPSase_s m_chain,	Glutamine amidotransferase class-I,Carbamoyl-phosphate synthase small chain, CPSase domain,	Carbamoyl-phosphate synthase, small subunit
PDIG_14060	1.08				hypothetical protein PDIP_39710
PDIG_28630	1.08	PF00793,	DAHP_synth_1,	DAHP synthetase I family,	Phospho-2-dehydro-3-deoxyheptonate aldolase, phenylalanine-inhibited
PDIG_84840	1.08	PF12929,	Mid1,	Stretch-activated Ca2+-permeable channel component,	Calcium channel subunit Mid1
PDIG_15670	1.08				hypothetical protein PDIG_15670
PDIG_36020	1.07	PF00012,	HSP70,	Hsp70 protein,	Hsp70 family chaperone Lhs1/Orp150, putative
PDIG_66320	1.07	PF00152,	tRNA-synt_2,	tRNA synthetases class II (D, K and N),	Aspartyl-tRNA synthetase Dps1, putative
