# Supplementary material for: Functional analysis of two sterol regulatory element binding proteins in Penicillium digitatum
Source: PLoS One. 2017 May 3;12(5):e0176485. doi: 10.1371/journal.pone.0176485 (PMC5415137; doi:10.1371/journal.pone.0176485)
Supplement: S7 Table — (PDF) [file pone.0176485.s011.pdf]

S7 Table. Annotation of genes uniquely regulated by *PdsreB*

ID	log2FC in △PdsreB	Pfam family	domain name(abbreviation)	domain name	nr_annotation
353 elements included exclusively in "△PdsreB-down":					
PDIG_67800	-12.77	PF00010,	HLH,	Helix-loop-helix DNA-binding domain,	Pc12g14660 [Penicillium chrysogenum Wisconsin 54-1255]
PDIG_17630	-8.96	PF00078,PF00075,	RVT_1,RNase_H,	Reverse transcriptase (RNA-dependent DNA polymerase),RNase H,	putative RNA-directed DNA polymerase from transposon X-element
PDIG_91320	-8.75	PF13976,PF07727,	gag_pre-integrs,RVT_2,	GAG-pre-integrase domain,Reverse transcriptase (RNA-dependent DNA polymerase),	hypothetical protein PDIG_91320
PDIG_17110	-5.72	PF00150,	Cellulase,	Cellulase (glycosyl hydrolase family 5),	Endo-1,4-beta-mannosidase
PDIG_89660	-5.69				hypothetical protein PDIG_45040
PDIG_88420	-5.51	PF05199,PF00732,	GMC_oxred_C,GMC_oxred_N,	GMC oxidoreductase,GMC oxidoreductase,	GMC oxidoreductase, putative
PDIG_47290	-5.43	PF07690,	MFS_1,	Major Facilitator Superfamily,	MFS multidrug transporter, putative
PDIG_50010	-5.26				hypothetical protein PDIG_50010
PDIG_46460	-5.15	PF03169,	OPT,	OPT oligopeptide transporter protein,	unnamed protein product [Aspergillus niger]
PDIG_89380	-5.13				hypothetical protein PDIG_89380
PDIG_32420	-4.91	PF00324,	AA_permease,	Amino acid permease,	hypothetical protein PDIP_52010
PDIG_70130	-4.88	PF03169,	OPT,	OPT oligopeptide transporter protein,	hypothetical protein PDIP_79440
PDIG_14230	-4.81	PF07727,	RVT_2,	Reverse transcriptase (RNA-dependent DNA polymerase),	hypothetical protein PDIG_87580
PDIG_42120	-4.70	PF13520,	AA_permease_2,	Amino acid permease,	hypothetical protein PDIG_42120
PDIG_71080	-4.68	PF04082,	Fungal_trans,	Fungal specific transcription factor	hypothetical protein PDIP_80400

				domain,	
PDIG_16450	-4.65	PF00069,	Pkinase,	Protein kinase domain,	hypothetical protein PDIP_87960
PDIG_06320	-4.52	PF11917,	DUF3435,	Protein of unknown function (DUF3435),	hypothetical protein PDIG_06320
PDIG_62200	-4.34	PF00450,	Peptidase_S10,	Serine carboxypeptidase,	Carboxypeptidase 5
PDIG_39030	-4.31	PF13976,PF07727,	gag_pre-integrs,RVT_2,	GAG-pre-integrase domain,Reverse transcriptase (RNA-dependent DNA polymerase),	hypothetical protein PDIG_91320
PDIG_89120	-4.18	PF02752,	Arrestin_C,	Arrestin (or S-antigen), C-terminal domain,	hypothetical protein PDIG_89120
PDIG_81770	-4.13	PF01112,	Asparaginase_2,	Asparaginase,	Putative isoaspartyl peptidase/L-asparaginase
PDIG_53460	-4.09	PF09084,	NMT1,	NMT1/THI5 like,	Thiamine biosynthesis protein
PDIG_71070	-4.01				hypothetical protein PDIP_80390
PDIG_36560	-3.86	PF07690,	MFS_1,	Major Facilitator Superfamily,	MFS transporter, putative
PDIG_16110	-3.84	PF13279,	4HBT_2,	Thioesterase-like superfamily,	hypothetical protein PDIP_87620
PDIG_62080	-3.78	PF00782,	DSPc,	Dual specificity phosphatase, catalytic domain,	Protein-tyrosine phosphatase, putative
PDIG_68690	-3.76	PF00703,	Glyco_hydro_2,	Glycosyl hydrolases family 2,	Glycosyl hydrolase, putative
PDIG_01760	-3.75	PF00026,	Asp,	Eukaryotic aspartyl protease,	Aspartic-type endopeptidase ctsD
PDIG_08000	-3.75	PF00324,	AA_permease,	Amino acid permease,	Proline permease, putative
PDIG_38910	-3.72				APSES transcription factor StuA
PDIG_50550	-3.67	PF00233,	PDEase_I,	3'5'-cyclic nucleotide phosphodiesterase,	High affinity cAMP phosphodiesterase, putative
PDIG_68190	-3.61	PF00324,	AA_permease,	Amino acid permease,	Amino acid transporter, putative
PDIG_00760	-3.57	PF00970,PF00175,	FAD_binding_6,NAD_binding_1,	Oxidoreductase FAD-binding domain,Oxidoreductase NAD-binding	hypothetical protein PDIG_00760

				domain,	
PDIG_43480	-3.57	PF01218,	Coprogen_oxidas,	Coproporphyrinogen III oxidase,	hypothetical protein PDIG_43480
PDIG_66480	-3.52				hypothetical protein PDIG_66480
PDIG_46640	-3.52	PF13450,PF01494,	NAD_binding_8,FAD_binding_3,	NAD(P)-binding Rossmann-like domain,FAD binding domain,	hypothetical protein PDIG_46640
PDIG_03400	-3.52	PF12874,	zf-met,	Zinc-finger of C2H2 type,	hypothetical protein PDIG_03400
PDIG_82790	-3.46				C6 transcription factor, putative
PDIG_41380	-3.44	PF05699,	Dimer_Tnp_hAT,	hAT family C-terminal dimerisation region,	hypothetical protein PDIP_86300
PDIG_29390	-3.42	PF11951,PF00172,	Fungal_trans_2,Zn_clus,	Fungal specific transcription factor domain,Fungal Zn(2)-Cys(6) binuclear cluster domain,	hypothetical protein PDIP_63820
PDIG_34690	-3.42				hypothetical protein PDIP_54260
PDIG_34480	-3.37	PF00107,PF08240,	ADH_zinc_N,ADH_N,	Zinc-binding dehydrogenase,Alcohol dehydrogenase GroES-like domain,	Alcohol dehydrogenase I
PDIG_62680	-3.34				hypothetical protein PDIP_72060
PDIG_02620	-3.33				hypothetical protein PDIG_02620
PDIG_02980	-3.28				General amino acid permease (Agp2)
PDIG_00900	-3.26	PF09286,	Pro-kuma_activ,	Pro-kumamolisin, activation domain,	Protease S8 tripeptidyl peptidase I, putative
PDIG_16030	-3.26	PF00501,	AMP-binding,	AMP-binding enzyme,	NRPS-like enzyme, putative
PDIG_50130	-3.24				hypothetical protein PDIG_50130
PDIG_55120	-3.23				hypothetical protein PDIG_55120
PDIG_25650	-3.23	PF00782,	DSPc,	Dual specificity phosphatase, catalytic domain,	Protein tyrosine phosphatase Pps1, putative
PDIG_75160	-3.21	PF00638,	Ran_BP1,	RanBP1 domain,	Nucleoporin nup61
PDIG_55710	-3.20				Pc14g00060 [Penicillium chrysogenum Wisconsin

					54-1255]
PDIG_24570	-3.19	PF03770,	IPK,	Inositol polyphosphate kinase,	Inositol hexaphosphate kinase KCS1, putative
PDIG_32170	-3.16	PF00610,PF00621,PF00780,PF15405,	DEP,RhoGEF,CNH,PH_5,	Domain found in Dishevelled, Egl-10, and Pleckstrin (DEP),RhoGEF domain,CNH domain,Pleckstrin homology domain,	Rho guanyl nucleotide exchange factor (Rom2), putative
PDIG_06110	-3.15	PF02219,	MTHFR,	Methylenetetrahydrofolate reductase,	Methylenetetrahydrofolate reductase
PDIG_32470	-3.15	PF13419,	HAD_2,	Haloacid dehalogenase-like hydrolase,	hypothetical protein PDIP_52050
PDIG_42130	-3.14				hypothetical protein PDIG_42130
PDIG_51670	-3.14	PF06920,PF14429,PF16172,	DHR-2,DOCK-C2,DOCK_N,	Dock homology region 2,C2 domain in Dock180 and Zizimin proteins,DOCK N-terminus,	hypothetical protein PDIG_51670
PDIG_07560	-3.12				hypothetical protein PDIP_81680
PDIG_89670	-3.11	PF03171,PF14226,	2OG-FeII_Oxy,DIOX_N,	2OG-Fe(II) oxygenase superfamily,non-haem dioxygenase in morphine synthesis N-terminal,	hypothetical protein PDIG_89670
PDIG_86770	-3.10	PF07690,	MFS_1,	Major Facilitator Superfamily,	hypothetical protein PDIG_86770
PDIG_12490	-3.06	PF01636,PF00534,	APH,Glycos_transf_1,	Phosphotransferase enzyme family,Glycosyl transferases group 1,	hypothetical protein PDIP_38710
PDIG_16460	-3.02	PF00170,	bZIP_1,	bZIP transcription factor,	hypothetical protein PDIP_87970
PDIG_50820	-3.02				hypothetical protein PDIG_50820
PDIG_67320	-3.02	PF05433,	Rick_17kDa_Anti,	Glycine zipper 2TM domain,	hypothetical protein PDIP_76620
PDIG_89680	-3.01	PF00668,PF00501,PF00550,	Condensation,AMP-binding,PP-binding,	Condensation domain,AMP-binding enzyme,Phosphopantetheine attachment site,	HC-toxin synthetase
PDIG_13070	-3.00				hypothetical protein PDIP_39290

PDIG_01300	-3.00	PF01717,PF08267,	Meth_synt_2,Meth_synt_1,	Cobalamin-independent synthase, Catalytic domain,Cobalamin-independent synthase, N-terminal domain,	Cobalamin-independent methionine synthase MetH/D
PDIG_02170	-2.99	PF00149,	Metallophos,	Calcineurin-like phosphoesterase,	Acid sphingomyelinase, putative
PDIG_91200	-2.93	PF00665,	rve,	Integrase core domain,	hypothetical protein PDIG_35890
PDIG_43140	-2.92	PF05699,	Dimer_Tnp_hAT,	hAT family C-terminal dimerisation region,	hypothetical protein PDIG_43140
PDIG_35910	-2.92	PF10431,PF00004,PF07724,	ClpB_D2-small,AAA,AAA_2,	C-terminal, D2-small domain, of ClpB protein,ATPase family associated with various cellular activities (AAA),AAA domain (Cdc48 subfamily),	Heat shock protein Hsp98/Hsp104/ClpA, putative
PDIG_50660	-2.91	PF00169,	PH,	PH domain,	hypothetical protein PDIG_50660
PDIG_69420	-2.90	PF03370,	CBM_21,	Carbohydrate/starch-binding module (family 21),	Protein phosphatase regulatory subunit Gac1, putative
PDIG_20460	-2.89	PF13602,	ADH_zinc_N_2,	Zinc-binding dehydrogenase,	Pc20g06850 [Penicillium chrysogenum Wisconsin 54-1255]
PDIG_85410	-2.89	PF04909,	Amidohydro_2,	Amidohydrolase,	Amidohydrolase family protein
PDIG_14340	-2.88	PF00067,	p450,	Cytochrome P450,	14-alpha sterol demethylase [Penicillium digitatum]
PDIG_51940	-2.88				hypothetical protein PDIG_51940
PDIG_84450	-2.86				hypothetical protein PDIG_84450
PDIG_67300	-2.86	PF06355,	Aegerolysin,	Aegerolysin,	hypothetical protein PDIP_76600
PDIG_17910	-2.84	PF04082,	Fungal_trans,	Fungal specific transcription factor domain,	hypothetical protein PDIP_55780

PDIG_78750	-2.83	PF07993,PF00698,PF08242,PF00550,PF00109,PF00668,PF02801,PF16197,PF14765,PF00501,PF13745,PF08659,	NAD_binding_4,Acyl_transf_1,Methyltransf_12,PP-binding,ketoacyl-synt,Condensation,Ketoacyl-synt_C,KAsynt_C_assoc,PS-DH,AMP-binding,HxxPF_rpt,KR,	Male sterility protein,Acyl transferase domain,Methyltransferase domain,Phosphopantetheine attachment site,Beta-ketoacyl synthase, N-terminal domain,Condensation domain,Beta-ketoacyl synthase, C-terminal domain,Ketoacyl-synthetase C-terminal extension,Polyketide synthase dehydratase,AMP-binding enzyme,HxxPF-repeated domain,KR domain,	Equisetin synthetase, putative
PDIG_14380	-2.83				hypothetical protein PDIG_14380
PDIG_26840	-2.82	PF00083,	Sugar_tr,	Sugar (and other) transporter,	hypothetical protein PDIP_61290
PDIG_49480	-2.81	PF00999,	Na_H_Exchanger,	Sodium/hydrogen exchanger family,	Plasma membrane antiporter, putative [Penicillium digitatum Pd1]
PDIG_83620	-2.80				hypothetical protein PDIP_89120
PDIG_48530	-2.77				hypothetical protein PDIP_57900
PDIG_86280	-2.77	PF12821,PF06738,	ThrE_2,ThrE,	Threonine/Serine exporter, ThrE,Putative threonine/serine exporter,	hypothetical protein PDIG_86280
PDIG_89880	-2.74	PF00665,PF07727,PF14223,	rve,RVT_2,Retrotran_gag_2,	Integrase core domain,Reverse transcriptase (RNA-dependent DNA polymerase),gag-polypeptide of LTR copia-type,	hypothetical protein PDIG_89880

PDIG_47920	-2.74	PF13967,PF14703,PF12621,PF02714,	RSN1_TM,PHM7_cyt,PHM7_ext,RSN1_7TM,	Late exocytosis, associated with Golgi transport,Cytosolic domain of 10TM putative phosphate transporter,Extracellular tail, of 10TM putative phosphate transporter,Calcium-dependent channel, 7TM region, putative phosphate,	hypothetical protein PDIP_57300
PDIG_39640	-2.73				hypothetical protein PDIG_39640
PDIG_17900	-2.71	PF00724,	Oxidored_FMN,	NADH:flavin oxidoreductase / NADH oxidase family,	12-oxophytodienoate reductase opr, putative
PDIG_10830	-2.71	PF04082,	Fungal_trans,	Fungal specific transcription factor domain,	C6 transcription factor, putative
PDIG_13800	-2.69	PF05699,	Dimer_Tnp_hAT,	hAT family C-terminal dimerisation region,	hypothetical protein PDIG_82610
PDIG_41740	-2.69	PF00670,PF05221,	AdoHcyase_NAD,AdoHcyase,	S-adenosyl-L-homocysteine hydrolase, NAD binding domain,S-adenosyl-L-homocysteine hydrolase,	Adenosylhomocysteinase
PDIG_89130	-2.67	PF01926,PF06071,	MMR_HSR1,YchF-GTPase_C,	50S ribosome-binding GTPase,Protein of unknown function (DUF933),	probable GTP-binding protein [Fusarium fujikuroi IMI 58289]
PDIG_77730	-2.67	PF00620,	RhoGAP,	RhoGAP domain,	Rho GTPase activator (Lrg11), putative
PDIG_17290	-2.66	PF00172,PF04082,	Zn_clus,Fungal_trans,	Fungal Zn(2)-Cys(6) binuclear cluster domain,Fungal specific transcription factor domain,	hypothetical protein PDIP_55190
PDIG_55570	-2.66	PF00069,	Pkinase,	Protein kinase domain,	Protein kinase, putative

PDIG_63820	-2.65	PF11563,	Protoglobin,	Protoglobin,	hypothetical protein PDIP_73170
PDIG_29960	-2.64				hypothetical protein PDIP_64340
PDIG_49980	-2.64	PF16197,PF00550,PF02801,PF14765,PF00109,PF08659,PF00698,	KAsynt_C_assoc,PP-binding,Ketoacyl-synt_C,PS-DH,ketoacyl-synt,KR,Acyl_transf_1,	Ketoacyl-synthetase C-terminal extension,Phosphopantetheine attachment site,Beta-ketoacyl synthase, C-terminal domain,Polyketide synthase dehydratase,Beta-ketoacyl synthase, N-terminal domain,KR domain,Acyl transferase domain,	Polyketide synthase, putative
PDIG_49050	-2.63	PF00690,PF00702,PF00122,PF13246,PF00689,	Cation_ATPase_N,Hydrolase,E1-E2_ATPase,Cation_ATPase,Cation_ATPase_C,	Cation transporter/ATPase, N-terminus,haloacid dehalogenase-like hydrolase,E1-E2 ATPase,Cation transport ATPase (P-type),Cation transporting ATPase, C-terminus,	Na/K ATPase alpha 1 subunit, putative
PDIG_81040	-2.62	PF07727,	RVT_2,	Reverse transcriptase (RNA-dependent DNA polymerase),	hypothetical protein PDIG_81040
PDIG_45650	-2.62	PF00806,	PUF,	Pumilio-family RNA binding repeat,	mRNA binding protein Pumilio 2, putative
PDIG_80370	-2.60	PF07690,	MFS_1,	Major Facilitator Superfamily,	hypothetical protein PDIG_80370
PDIG_04940	-2.60	PF02518,PF00512,PF00072,	HATPase_c,HisKA,Response_reg,	Histidine kinase-, DNA gyrase B-, and HSP90-like ATPase,His Kinase A (phospho-acceptor) domain,Response regulator receiver domain,	Sensor histidine kinase/response regulator, putative
PDIG_79950	-2.59	PF00078,PF14529,	RVT_1,Exo_endo_phos_2,	Reverse transcriptase (RNA-dependent DNA polymerase),Endonuclease-reverse	hypothetical protein PDIG_79950

				transcriptase,	
PDIG_66170	-2.59	PF03098,	An_peroxidase,	Animal haem peroxidase,	Psi-producing oxygenase C
PDIG_29210	-2.58	PF00121,	TIM,	Triosephosphate isomerase,	Triosephosphate isomerase
PDIG_32550	-2.58				hypothetical protein PDIP_52120
PDIG_21470	-2.56	PF00296,	Bac_luciferase,	Luciferase-like monooxygenase,	Xenobiotic compound monooxygenase, DszA family
PDIG_26930	-2.56				hypothetical protein PDIP_61380
PDIG_28310	-2.56	PF03441,PF00875,	FAD_binding_7,DNA_photolyase,	FAD binding domain of DNA photolyase,DNA photolyase,	hypothetical protein PDIP_62750
PDIG_59270	-2.55	PF00069,	Pkinase,	Protein kinase domain,	hypothetical protein PDIP_68710
PDIG_76460	-2.55				hypothetical protein PDIG_76460
PDIG_02130	-2.54	PF00317,PF03477,PF02867,	Ribonuc_red_lgN,ATP-cone,Ribonuc_red_lgC,	Ribonucleotide reductase, all-alpha domain,ATP cone domain,Ribonucleotide reductase, barrel domain,	Ribonucleoside-diphosphate reductase
PDIG_04980	-2.52	PF00704,PF00187,	Glyco_hydro_18,Chitin_bind_1,	Glycosyl hydrolases family 18,Chitin recognition protein,	Class V chitinase, putative
PDIG_39020	-2.50				hypothetical protein PDIG_39020
PDIG_87820	-2.50	PF13450,	NAD_binding_8,	NAD(P)-binding Rossmann-like domain,	Pc22g06180 [Penicillium chrysogenum Wisconsin 54-1255]
PDIG_50830	-2.46	PF01740,PF00916,	STAS,Sulfate_transp,	STAS domain,Sulfate permease family,	Sulfate permease SutB
PDIG_18760	-2.45	PF13641,	Glyco_tranf_2_3,	Glycosyltransferase like family 2,	Polysaccharide synthase Cps1, putative
PDIG_89260	-2.45				BZIP transcription factor, putative
PDIG_25710	-2.44	PF01764,PF00450,PF03893,	Lipase_3,Peptidase_S10,Lipase3_N,	Lipase (class 3),Serine carboxypeptidase,Lipase 3 N-terminal region,	Pc21g06670 [Penicillium chrysogenum Wisconsin 54-1255]

PDIG_35660	-2.44	PF00324,	AA_permease,	Amino acid permease,	hypothetical protein PDIG_35660
PDIG_34880	-2.43	PF09949,	DUF2183,	Uncharacterized conserved protein (DUF2183),	hypothetical protein PDIG_34880
PDIG_02360	-2.41	PF00617,PF00618,	RasGEF,RasGEF_N,	RasGEF domain,RasGEF N-terminal motif,	Ras guanine-nucleotide exchange protein, putative
PDIG_55700	-2.41	PF02801,PF07993,PF00698,PF13847,PF08659,PF13745,PF00109,PF00501,PF14765,PF00668,PF00550,PF16197,	Ketoacyl-synt_C,NAD_binding_4,Acyl_transf_1,Methyltransf_31,KR,HxxPF_rpt,ketoacyl-synt,AMP-binding,PS-DH,Condensation,PP-binding,KAsynt_C_assoc,	Beta-ketoacyl synthase, C-terminal domain,Male sterility protein,Acyl transferase domain,Methyltransferase domain,KR domain,HxxPF-repeated domain,Beta-ketoacyl synthase, N-terminal domain,AMP-binding enzyme,Polyketide synthase dehydratase,Condensation domain,Phosphopantetheine attachment site,Ketoacyl-synthetase C-terminal extension,	Hybrid NRPS/PKS enzyme, putative
PDIG_53880	-2.40				hypothetical protein PDIG_53880
PDIG_14870	-2.39	PF00520,	Ion_trans,	Ion transport protein,	Calcium channel subunit Cch1
PDIG_11590	-2.39	PF00665,PF08284,PF00078,PF00098,	rve,RVP_2,RVT_1,zf-CCHC,	Integrase core domain,Retroviral aspartyl protease,Reverse transcriptase (RNA-dependent DNA polymerase),Zinc knuckle,	Retrotransposon polyprotein, putative
PDIG_23100	-2.39				hypothetical protein PDIG_23100
PDIG_66750	-2.39	PF00464,	SHMT,	Serine hydroxymethyltransferase,	Serine hydroxymethyltransferase
PDIG_54860	-2.37	PF00294,	PfkB,	pfkB family carbohydrate kinase,	Adenosine kinase, putative
PDIG_29270	-2.34				hypothetical protein PDIP_63700

PDIG_20350	-2.34	PF11001,PF10998,	DUF2841,DUF2838,	Protein of unknown function (DUF2841),Protein of unknown function (DUF2838),	hypothetical protein PDIP_00250 [Penicillium digitatum Pd1]
PDIG_11000	-2.34	PF01179,	Cu_amine_oxid,	Copper amine oxidase, enzyme domain,	Pc21g04560 [Penicillium chrysogenum Wisconsin 54-1255]
PDIG_11220	-2.33	PF00349,PF03727,	Hexokinase_1,Hexokinase_2,	Hexokinase,Hexokinase,	Hexokinase-1
PDIG_34850	-2.32				hypothetical protein PDIP_54420
PDIG_12860	-2.31				MFS amine transporter, putative
PDIG_35390	-2.31	PF02136,PF00076,	NTF2,RRM_1,	Nuclear transport factor 2 (NTF2) domain,RNA recognition motif. (a.k.a. RRM, RBD, or RNP domain),	hypothetical protein PDIP_54940
PDIG_20730	-2.31	PF05544,	Pro_racemase,	Proline racemase,	hypothetical protein PDIP_88540
PDIG_15720	-2.31	PF02773,PF00438,PF02772,	S-AdoMet_synt_C,S-AdoMet_synt_N,S-AdoMet_synt_M,	S-adenosylmethionine synthetase, C-terminal domain,S-adenosylmethionine synthetase, N-terminal domain,S-adenosylmethionine synthetase, central domain,	S-adenosylmethionine synthase
PDIG_70310	-2.31				hypothetical protein PDIP_79620
PDIG_16150	-2.31	PF00501,PF00550,PF00668,	AMP-binding,PP-binding,Condensation,	AMP-binding enzyme,Phosphopantetheine attachment site,Condensation domain,	HC-toxin synthetase
PDIG_66780	-2.31	PF00136,PF08490,PF03104,	DNA_pol_B,DUF1744,DNA_pol_B_exo1,	DNA polymerase family B,Domain of unknown function (DUF1744),DNA polymerase family B, exonuclease	DNA polymerase epsilon catalytic subunit A

				domain,	
PDIG_68080	-2.30				hypothetical protein PDIP_77370
PDIG_36420	-2.29	PF07690,	MFS_1,	Major Facilitator Superfamily,	MFS transporter, putative
PDIG_42090	-2.28	PF00155,	Aminotran_1_2,	Aminotransferase class I and II,	hypothetical protein PDIG_42090
PDIG_62980	-2.28	PF01532,	Glyco_hydro_47,	Glycosyl hydrolase family 47,	putative class I alpha-mannosidase
PDIG_87580	-2.28	PF07727,	RVT_2,	Reverse transcriptase (RNA-dependent DNA polymerase),	hypothetical protein PDIG_87580
PDIG_24080	-2.27				hypothetical protein PDIP_58580
PDIG_34460	-2.26				hypothetical protein PDIP_54020
PDIG_49080	-2.25	PF00621,	RhoGEF,	RhoGEF domain,	Rho guanyl nucleotide exchange factor, putative
PDIG_64750	-2.25	PF00617,PF00618,	RasGEF,RasGEF_N,	RasGEF domain,RasGEF N-terminal motif,	Guanine nucleotide exchange factor, putative
PDIG_68720	-2.24	PF07727,PF00665,	RVT_2,rve,	Reverse transcriptase (RNA-dependent DNA polymerase),Integrase core domain,	hypothetical protein PDIG_35550
PDIG_71260	-2.24	PF07727,PF00665,	RVT_2,rve,	Reverse transcriptase (RNA-dependent DNA polymerase),Integrase core domain,	hypothetical protein PDIG_35550
PDIG_79430	-2.24				Cell wall proline rich protein, putative
PDIG_13590	-2.22	PF02714,PF12621,PF14703,	RSN1_7TM,PHM7_ext,PHM7_cyt,	Calcium-dependent channel, 7TM region, putative phosphate,Extracellular tail, of 10TM putative phosphate transporter,Cytosolic domain of 10TM putative phosphate transporter,	hypothetical protein PDIP_47710

PDIG_33500	-2.21	PF12848,PF00005,	ABC_tran_Xtn,ABC_tran,	ABC transporter,ABC transporter,	Ribosome biogenesis ABC transporter Arb1, putative
PDIG_55720	-2.21	PF07690,	MFS_1,	Major Facilitator Superfamily,	MFS multidrug transporter, putative
PDIG_70020	-2.19	PF00271,PF00176,	Helicase_C,SNF2_N,	Helicase conserved C-terminal domain,SNF2 family N-terminal domain,	SNF2 family helicase/ATPase, putative
PDIG_03800	-2.19				hypothetical protein PDIG_03800
PDIG_46390	-2.18	PF00171,	Aldedh,	Aldehyde dehydrogenase family,	hypothetical protein PDIG_46390
PDIG_73870	-2.18				putative WetA [Penicillium digitatum]
PDIG_04660	-2.17	PF02913,PF01565,	FAD-oxidase_C,FAD_binding_4,	FAD linked oxidases, C-terminal domain,FAD binding domain,	Oxidoreductase, FAD-binding, putative
PDIG_82690	-2.16	PF00732,	GMC_oxred_N,	GMC oxidoreductase,	Aryl-alcohol dehydrogenase, putative
PDIG_45090	-2.16	PF00145,	DNA_methylase,	C-5 cytosine-specific DNA methylase,	C-5 cytosine methyltransferase DmtA [Penicillium digitatum Pd1]
PDIG_26250	-2.15	PF00071,	Ras,	Ras family,	RAS protein
PDIG_61040	-2.14	PF13805,	Pil1,	Eisosome component PIL1,	Cell wall integrity signaling protein Lsp1/Pil1, putative
PDIG_71890	-2.14	PF10017,PF12867,PF03781,	Methyltransf_33,DinB_2,FGE-sulfatase,	Histidine-specific methyltransferase, SAM-dependent,DinB superfamily,Sulfatase-modifying factor enzyme 1,	hypothetical protein PDIP_81160
PDIG_77910	-2.14	PF00388,PF00387,	PI-PLC-X,PI-PLC-Y,	Phosphatidylinositol-specific phospholipase C, X domain,Phosphatidylinositol-specific phospholipase C, Y domain,	1-phosphatidylinositol-4,5-bisphosphate phosphodiesterase 1, putative
PDIG_50650	-2.13				hypothetical protein PDIG_50650

PDIG_04950	-2.13	PF00171,PF04909,	Aldedh,Amidohydro_2,	Aldehyde dehydrogenase family,Amidohydrolase,	Putative succinate-semialdehyde dehydrogenase
PDIG_44820	-2.13				hypothetical protein PDIG_44820
PDIG_49690	-2.13	PF04082,	Fungal_trans,	Fungal specific transcription factor domain,	hypothetical protein PDIG_49690
PDIG_05530	-2.13				Dimethylaniline monooxygenase, putative
PDIG_34760	-2.13	PF07819,	PGAP1,	PGAP1-like protein,	GPI inositol-deacylase
PDIG_03810	-2.12				hypothetical protein PDIG_03810
PDIG_49840	-2.12	PF00665,PF13650,PF00078,	rve,Asp_protease_2,RVT_1,	Integrase core domain,Aspartyl protease,Reverse transcriptase (RNA-dependent DNA polymerase),	hypothetical protein PDIG_86730
PDIG_69480	-2.12				hypothetical protein PDIP_78770
PDIG_26960	-2.11				hypothetical protein PDIP_61410
PDIG_15930	-2.11				hypothetical protein PDIG_15930
PDIG_80580	-2.10	PF00069,	Pkinase,	Protein kinase domain,	Protein serine/threonine kinase (Ran1), putative
PDIG_26910	-2.10				Flavin-binding monooxygenase-like protein
PDIG_15090	-2.09	PF00109,PF08242,PF14765,PF16197,PF08659,PF00107,PF08240,PF00550,PF00698,PF02801,	ketoacyl-synt,Methyltransf_12,PS-DH,KAsynt_C_assoc,KR,ADH_zinc_N,ADH_N,PP-binding,Acyl_transf_1,Ketoacyl-synt_C,	Beta-ketoacyl synthase, N-terminal domain,Methyltransferase domain,Polyketide synthase dehydratase,Ketoacyl-synthetase C-terminal extension,KR domain,Zinc-binding dehydrogenase,Alcohol dehydrogenase GroES-like domain,Phosphopantetheine attachment site,Acyl transferase	hypothetical protein PDIG_15090

				domain,Beta-ketoacyl synthase, C-terminal domain,	
PDIG_49990	-2.09	PF00005,PF06422,PF01061,	ABC_tran,PDR_CDR,ABC2_membrane,	ABC transporter,CDR ABC transporter,ABC-2 type transporter,	ABC multidrug transporter, putative
PDIG_78740	-2.08				hypothetical protein PDIG_78740
PDIG_02160	-2.08				hypothetical protein PDIG_02160
PDIG_77810	-2.08	PF00924,	MS_channel,	Mechanosensitive ion channel,	Mechanosensitive ion channel family
PDIG_58790	-2.08				hypothetical protein PDIP_68250
PDIG_24950	-2.08	PF01753,	zf-MYND,	MYND finger,	hypothetical protein PDIP_59430
PDIG_19220	-2.07	PF00071,	Ras,	Ras family,	RAS small monomeric GTPase, putative
PDIG_12870	-2.06				Carboxylesterase, putative
PDIG_34540	-2.06	PF04488,	Gly_transf_sug,	Glycosyltransferase sugar-binding region containing DXD motif,	hypothetical protein PDIP_54100
PDIG_82460	-2.06				hypothetical protein PDIP_88700
PDIG_65320	-2.06				BZIP transcription factor (MeaB), putative
PDIG_18290	-2.06	PF01231,	IDO,	Indoleamine 2,3-dioxygenase,	Indoleamine 2,3-dioxygenase family protein
PDIG_90240	-2.05	PF13185,	GAF_2,	GAF domain,	hypothetical protein PDIG_90240
PDIG_43750	-2.05	PF00069,	Pkinase,	Protein kinase domain,	Meiosis induction protein kinase (Ime2), putative
PDIG_87640	-2.04	PF16193,PF00004,PF12002,	AAA_assoc_2,AAA,MgsA_C,	AAA C-terminal domain,ATPase family associated with various cellular activities (AAA),MgsA AAA+ ATPase C terminal,	AAA family ATPase, putative
PDIG_00600	-2.04	PF06330,	TRI5,	Trichodiene synthase (TRI5),	Trichodiene synthase
PDIG_14310	-2.04	PF00225,	Kinesin,	Kinesin motor domain,	Kinesin family protein (KipA), putative

PDIG_43540	-2.02				hypothetical protein PDIG_43540
PDIG_24710	-2.02	PF14226,PF 03171,	DIOX_N,2OG-FeII_Oxy,	non-haem dioxygenase in morphine synthesis N-terminal,2OG-Fe(II) oxygenase superfamily,	Oxidoreductase, 2OG-Fe(II) oxygenase family
PDIG_60300	-2.02	PF00226,	DnaJ,	DnaJ domain,	hypothetical protein PDIP_69710
PDIG_86500	-2.02	PF01946,	Thi4,	Thi4 family,	Thiazole biosynthesis enzyme
PDIG_36110	-2.01				Pc06g00520 [Penicillium chrysogenum Wisconsin 54-1255]
PDIG_63130	-2.01	PF12874,PF 00096,	zf-met,zf-C2H2,	Zinc-finger of C2H2 type,Zinc finger, C2H2 type,	hypothetical protein PDIP_72510
PDIG_90000	-2.00	PF13692,	Glyco_trans_1_4,	Glycosyl transferases group 1,	Glycosyl transferase, putative
PDIG_64590	-2.00	PF03221,PF 04218,	HTH_Tnp_Tc5,CENP-B_ N,	Tc5 transposase DNA-binding domain,CENP-B N-terminal DNA-binding domain,	hypothetical protein PDIP_73930
PDIG_68630	-2.00	PF00365,	PFK,	Phosphofructokinase,	6-phosphofructokinase
PDIG_13110	-2.00	PF14833,PF 03446,	NAD_binding_11,NAD_bi nding_2,	NAD-binding of NADP-dependent 3-hydroxyisobutyrate dehydrogenase,NAD binding domain of 6-phosphogluconate dehydrogenase,	Putative 3-hydroxyisobutyrate dehydrogenase
PDIG_34870	-1.99	PF02133,	Transp_cyt_pur,	Permease for cytosine/purines, uracil, thiamine, allantoin,	Nucleoside transporter, putative
PDIG_45640	-1.99				hypothetical protein PDIG_45640
PDIG_66680	-1.99	PF11704,	Folliculin,	Vesicle coat protein involved in Golgi to plasma membrane transport,	hypothetical protein PDIP_75980
PDIG_42950	-1.99	PF12697,	Abhydrolase_6,	Alpha/beta hydrolase family,	Hydrolase, alpha/beta fold family protein
PDIG_83780	-1.99	PF03184,	DDE_1,	DDE superfamily endonuclease,	hypothetical protein PDIG_83780

PDIG_36530	-1.99	PF14534,	DUF4440,	Domain of unknown function (DUF4440),	hypothetical protein PDIP_83130
PDIG_39390	-1.98	PF00122,PF13246,PF16209,PF16212,	E1-E2_ATPase,Cation_ATPase,PhoLip_ATPase_N,PhoLip_ATPase_C,	E1-E2 ATPase,Cation transport ATPase (P-type),Phospholipid-translocating ATPase N-terminal,Phospholipid-translocating P-type ATPase C-terminal,	Phospholipid-transporting ATPase (DRS2), putative
PDIG_38320	-1.98	PF12030,PF00443,	DUF3517,UCH,	Domain of unknown function (DUF3517),Ubiquitin carboxyl-terminal hydrolase,	Ubiquitin Carboxy-terminal hydrolase, putative
PDIG_31720	-1.98				hypothetical protein PDIP_51300
PDIG_59910	-1.97	PF07690,	MFS_1,	Major Facilitator Superfamily,	MFS multidrug transporter, putative
PDIG_46240	-1.97				hypothetical protein PDIG_46240
PDIG_50950	-1.97	PF10446,	DUF2457,	Protein of unknown function (DUF2457),	hypothetical protein PDIG_50950
PDIG_28670	-1.97	PF14701,PF06202,PF14702,PF14699,	hDGE_amylase,GDE_C,hGDE_central,hGDE_N,	Glucanotransferase of human glycogen debranching enzyme,Amylo-alpha-1,6-glucosidase, Central domain of human glycogen debranching enzyme,N-terminal domain from the human glycogen debranching enzyme,	Glycogen debranching enzyme Gdb1, putative
PDIG_24530	-1.96	PF00225,	Kinesin,	Kinesin motor domain,	Kinesin family protein
PDIG_47310	-1.95	PF00083,	Sugar_tr,	Sugar (and other) transporter,	MFS monosaccharide transporter, putative
PDIG_81190	-1.95				hypothetical protein PDIG_81190

PDIG_50670	-1.94	PF00295,	Glyco_hydro_28,	Glycosyl hydrolases family 28,	PG2 [Penicillium digitatum]
PDIG_83160	-1.94				hypothetical protein PDIP_86950
PDIG_69900	-1.94	PF00013,	KH_1,	KH domain,	hypothetical protein PDIP_79190
PDIG_12460	-1.93	PF03367,	zf-ZPR1,	ZPR1 zinc-finger domain,	Zinc finger protein ZPR1
PDIG_00690	-1.93	PF02045,	CBFB_NFYA,	CCAAT-binding transcription factor (CBF-B/NF-YA) subunit B,	CCAAT-binding transcription factor subunit HAPB
PDIG_25480	-1.93				hypothetical protein PDIP_59960
PDIG_50200	-1.92	PF00512,PF00072,PF02518,	HisKA,Response_reg,HATPase_c,	His Kinase A (phospho-acceptor) domain,Response regulator receiver domain,Histidine kinase-, DNA gyrase B-, and HSP90-like ATPase,	hypothetical protein PDIG_50200
PDIG_55890	-1.92	PF13358,PF01498,	DDE_3,HTH_Tnp_Tc3_2,	DDE superfamily endonuclease,Transposase,	hypothetical protein PDIG_55890
PDIG_78420	-1.91	PF02373,PF02375,PF13832,	JmjC,JmjN,zf-HC5HC2H_2,	JmjC domain, hydroxylase,jmjN domain,PHD-zinc-finger like domain,	Jumonji family transcription factor, putative
PDIG_24490	-1.91	PF00650,PF03765,	CRAL_TRIO,CRAL_TRIO_N,	CRAL/TRIO domain,CRAL/TRIO, N-terminal domain,	hypothetical protein PDIP_58970
PDIG_05360	-1.91				Plasma membrane ATPase
PDIG_16320	-1.90	PF06314,PF01494,	ADC,FAD_binding_3,	Acetoacetate decarboxylase (ADC),FAD binding domain,	Salicylate hydroxylase, putative
PDIG_34860	-1.90	PF02133,	Transp_cyt_pur,	Permease for cytosine/purines, uracil, thiamine, allantoin,	Vitamin B6 transporter, putative
PDIG_47970	-1.90	PF00702,PF00403,PF00122,	Hydrolase,HMA,E1-E2_ATPase,	haloacid dehalogenase-like hydrolase,Heavy-metal-associated domain,E1-E2 ATPase,	Copper-transporting ATPase, putative

PDIG_80800	-1.87	PF08544,PF00288,PF10509,	GHMP_kinases_C,GHMP_kinases_N,GalKase_gal_bdg,	GHMP kinases C terminal,GHMP kinases N terminal domain,Galactokinase galactose-binding signature,	Galactokinase
PDIG_31670	-1.87	PF01536,	SAM_decarbox,	Adenosylmethionine decarboxylase,	S-adenosylmethionine decarboxylase proenzyme
PDIG_46160	-1.87				hypothetical protein PDIG_46160
PDIG_37510	-1.87	PF00722,	Glyco_hydro_16,	Glycosyl hydrolases family 16,	Endo-1,3(4)-beta-glucanase, putative
PDIG_01070	-1.86	PF08625,PF00400,	Utp13,WD40,	Utp13 specific WD40 associated domain,WD domain, G-beta repeat,	Small nucleolar ribonucleoprotein complex subunit, putative
PDIG_46280	-1.85	PF13646,PF08609,	HEAT_2,Fes1,	HEAT repeats,Nucleotide exchange factor Fes1,	Hsp70 nucleotide exchange factor (Fes1), putative
PDIG_90700	-1.85	PF14510,PF06422,PF01061,PF00005,	ABC_trans_N,PDR_CDR,ABC2_membrane,ABC_tran,	ABC-transporter extracellular N-terminal,CDR ABC transporter,ABC-2 type transporter,ABC transporter,	ABC multidrug transporter, putative
PDIG_79050	-1.84	PF09783,	Vac_ImportDeg,	Vacuolar import and degradation protein,	Vesicle-mediated transport protein Vid24, putative
PDIG_64970	-1.84	PF00933,PF13508,	Glyco_hydro_3,Acetyltransf_7,	Glycosyl hydrolase family 3 N terminal domain,Acetyltransferase (GNAT) domain,	Beta-N-acetylglucosaminidase, putative
PDIG_34470	-1.83	PF13520,	AA_permease_2,	Amino acid permease,	GABA permease, putative
PDIG_39590	-1.83	PF00668,PF00501,PF00550,	Condensation,AMP-binding,PP-binding,	Condensation domain,AMP-binding enzyme,Phosphopantetheine attachment site,	hypothetical protein PDIP_25130 [Penicillium digitatum Pd1]
PDIG_77800	-1.83	PF00069,PF00072,	Pkinase,Response_reg,	Protein kinase domain,Response regulator receiver domain,	Serine threonine protein kinase, putative

PDIG_41820	-1.83				hypothetical protein PDIG_41820
PDIG_83030	-1.83	PF00067,	p450,	Cytochrome P450,	hypothetical protein PDIG_83030
PDIG_18130	-1.82	PF04082,	Fungal_trans,	Fungal specific transcription factor domain,	C6 transcription factor, putative
PDIG_50810	-1.82	PF01556,PF00226,PF00684,	DnaJ_C,DnaJ,DnaJ_CXXCXGXG,	DnaJ C terminal domain,DnaJ domain,DnaJ central domain,	Protein mitochondrial targeting protein (Mas1), putative
PDIG_68680	-1.82	PF03663,	Glyco_hydro_76,	Glycosyl hydrolase family 76,	Cell wall glycosyl hydrolase Dfg5, putative
PDIG_03820	-1.82	PF07986,	TBCC,	Tubulin binding cofactor C,	Tubulin-specific chaperone c, putative
PDIG_30190	-1.81	PF01753,PF00856,	zf-MYND,SET,	MYND finger,SET domain,	hypothetical protein PDIP_64570
PDIG_68530	-1.81	PF07690,	MFS_1,	Major Facilitator Superfamily,	2-ketogluconate transporter, putative
PDIG_10760	-1.81	PF13002,	LDB19,	Arrestin_N terminal like,	hypothetical protein PDIP_82270
PDIG_06360	-1.81				hypothetical protein PDIG_06360
PDIG_59190	-1.81				hypothetical protein PDIP_68640
PDIG_00210	-1.81				hypothetical protein PDIG_00210
PDIG_81060	-1.81	PF00072,PF00512,PF02518,	Response_reg,HisKA,HATPase_c,	Response regulator receiver domain,His Kinase A (phospho-acceptor) domain,Histidine kinase-, DNA gyrase B-, and HSP90-like ATPase,	Sensor histidine kinase/response regulator, putative
PDIG_13980	-1.80	PF03456,PF02141,	uDENN,DENN,	uDENN domain,DENN (AEX-3) domain,	hypothetical protein PDIG_13980
PDIG_35680	-1.80	PF04082,PF00172,	Fungal_trans,Zn_clus,	Fungal specific transcription factor domain,Fungal Zn(2)-Cys(6) binuclear cluster domain,	C6 transcription factor, putative

PDIG_65370	-1.78	PF00270,PF00271,	DEAD,Helicase_C,	DEAD/DEAH box helicase,Helicase conserved C-terminal domain,	ATP dependent RNA helicase (Dbp8), putative
PDIG_59920	-1.77	PF01073,	3Beta_HSD,	3-beta hydroxysteroid dehydrogenase/isomerase family,	3beta-hydroxysteroid-dehydrogenase/decarboxylase isoform 2
PDIG_40520	-1.76	PF04898,PF14691,PF01645,PF00310,PF07992,PF01493,	Glu_syn_central,Fer4_20,Glu_synthase,GATase_2,Pyr_redox_2,GXGXG,	Glutamate synthase central domain,Dihydroprymidine dehydrogenase domain II, 4Fe-4S cluster,Conserved region in glutamate synthase,Glutamine amidotransferases class-II,Pyridine nucleotide-disulphide oxidoreductase,GXGXG motif,	Glutamate synthase Glt1, putative
PDIG_54570	-1.76	PF00128,PF08323,	Alpha-amylase,Glyco_transf_5,	Alpha amylase, catalytic domain,Starch synthase catalytic domain,	alpha-1,3-glucan synthase [Aspergillus kawachii IFO 4308]
PDIG_36330	-1.76				hypothetical protein PDIG_36330
PDIG_15580	-1.76				hypothetical protein PDIG_15580
PDIG_79440	-1.75	PF00782,	DSPc,	Dual specificity phosphatase, catalytic domain,	Dual specificity protein phosphatase 3, putative
PDIG_64510	-1.75	PF05686,	Glyco_transf_90,	Glycosyl transferase family 90,	Capsule-associated protein CAP1, putative
PDIG_82720	-1.75	PF01699,	Na_Ca_ex,	Sodium/calcium exchanger protein,	Vacuolar H+\/Ca2+ exchanger
PDIG_00350	-1.74	PF08316,	Pal1,	Pal1 cell morphology protein,	hypothetical protein PDIG_00350
PDIG_66530	-1.74	PF06687,	SUR7,	SUR7/PalI family,	hypothetical protein PDIP_75830
PDIG_88550	-1.74	PF00005,PF00664,	ABC_tran,ABC_membrane,	ABC transporter,ABC transporter transmembrane region,	ABC multidrug transporter, putative
PDIG_47900	-1.74	PF14551,PF00493,	MCM_N,MCM,	MCM N-terminal domain,MCM2/3/5 family,	DNA replication licensing factor Mcm6, putative

PDIG_56130	-1.73	PF04116,PF00173,	FA_hydroxylase,Cyt-b5,	Fatty acid hydroxylase superfamily,Cytochrome b5-like Heme/Steroid binding domain,	Fatty acid hydroxylase, putative
PDIG_02150	-1.73	PF03600,	CitMHS,	Citrate transporter,	Arsenite efflux transporter ArsB-like, putative
PDIG_73220	-1.73				hypothetical protein PDIG_73220
PDIG_21940	-1.72	PF14551,PF12619,PF00493,	MCM_N,MCM2_N,MCM,	MCM N-terminal domain,Mini-chromosome maintenance protein 2,MCM2/3/5 family,	hypothetical protein PDIG_21940
PDIG_36400	-1.72	PF13489,	Methyltransf_23,	Methyltransferase domain,	hypothetical protein PDIG_36400
PDIG_74650	-1.72	PF00665,PF07727,PF14223,	rve,RVT_2,Retrotran_gag_2,	Integrase core domain,Reverse transcriptase (RNA-dependent DNA polymerase),gag-polypeptide of LTR copia-type,	hypothetical protein PDIG_74650
PDIG_61030	-1.72	PF07716,	bZIP_2,	Basic region leucine zipper,	BZIP transcription factor, putative
PDIG_20100	-1.72	PF09497,	Med12,	Transcription mediator complex subunit Med12,	Mediator of RNA polymerase II transcription subunit 12
PDIG_59590	-1.71				Pathogenesis associated protein Cap20, putative
PDIG_75880	-1.71	PF05730,	CFEM,	CFEM domain,	hypothetical protein PDIG_75880
PDIG_68890	-1.71				MLG1, putative
PDIG_76300	-1.70	PF16073,PF00109,PF00550,PF02801,PF00698,PF07993,PF08242,	SAT,ketoacyl-synt,PP-binding,Ketoacyl-synt_C,Acyl_transf_1,NAD_binding_4,Methyltransf_12,	Starter unit:ACP transacylase in aflatoxin biosynthesis,Beta-ketoacyl synthase, N-terminal domain,Phosphopantetheine attachment site,Beta-ketoacyl synthase, C-terminal domain,Acyl	Polyketide synthase, putative

				transferase domain,Male sterility protein,Methyltransferase domain,	
PDIG_44600	-1.70	PF08161,	NUC173,	NUC173 domain,	Pre-rRNA processing protein Rrp12, putative
PDIG_63830	-1.70	PF02836,PF00703,	Glyco_hydro_2_C,Glyco_hydro_2,	Glycosyl hydrolases family 2, TIM barrel domain,Glycosyl hydrolases family 2,	hydrolase, putative [Aspergillus oryzae 3.042]
PDIG_75450	-1.70	PF03178,PF10433,	CPSF_A,MMS1_N,	CPSF A subunit region,Mono-functional DNA-alkylating methyl methanesulfonate N-term,	Pre-mRNA-splicing factor rse1
PDIG_82970	-1.69	PF00491,	Arginase,	Arginase family,	hypothetical protein PDIP_86760
PDIG_79730	-1.69	PF13806,PF07992,PF03460,PF01077,	Rieske_2,Pyr_redox_2,NIR_SIR_ferr,NIR_SIR,	Rieske-like [2Fe-2S] domain,Pyridine nucleotide-disulphide oxidoreductase,Nitrite/Sulfite reductase ferredoxin-like half domain,Nitrite and sulphite reductase 4Fe-4S domain,	Pc13g11420 [Penicillium chrysogenum Wisconsin 54-1255]
PDIG_08540	-1.69	PF04185,	Phosphoesterase,	Phosphoesterase family,	Phospholipase C PLC-B
PDIG_32560	-1.69				hypothetical protein PDIP_52130
PDIG_76140	-1.69	PF02538,PF01968,PF05378,	Hydantoinase_B,Hydantoinase_A,Hydant_A_N,	Hydantoinase B/oxoprolinase,Hydantoinase/oxoprolinase,Hydantoinase/oxoprolinase N-terminal region,	5-oxo-L-prolinase, putative
PDIG_71510	-1.69	PF02301,	HORMA,	HORMA domain,	Mitotic spindle checkpoint protein (Mad2B), putative
PDIG_79200	-1.68	PF13650,PF00078,PF00	Asp_protease_2,RVT_1,rve,	Aspartyl protease,Reverse transcriptase (RNA-dependent DNA	hypothetical protein PDIG_79200

		665,		polymerase),Integrase core domain,	
PDIG_25500	-1.68	PF00400,PF11816,	WD40,DUF3337,	WD domain, G-beta repeat,Domain of unknown function (DUF3337),	WD repeat protein
PDIG_45590	-1.68	PF03542,PF11864,PF02145,	Tuberin,DUF3384,Rap_GAP,	Tuberin,Domain of unknown function (DUF3384),Rap/ran-GAP,	GTPase activating protein (Tsc2), putative
PDIG_11400	-1.67				hypothetical protein PDIP_82900
PDIG_65820	-1.66				hypothetical protein PDIP_75140
PDIG_86930	-1.66	PF00400,	WD40,	WD domain, G-beta repeat,	hypothetical protein PDIG_86930
PDIG_20490	-1.66	PF01476,	LysM,	LysM domain,	hypothetical protein PDIG_20490
PDIG_42000	-1.65	PF11715,	Nup160,	Nucleoporin Nup120/160,	hypothetical protein PDIG_42000
PDIG_34190	-1.65				hypothetical protein PDIG_39320
PDIG_69060	-1.64	PF10350,	DUF2428,	Putative death-receptor fusion protein (DUF2428),	HEAT repeat protein
PDIG_82830	-1.64	PF14636,	FNIP_N,	Folliculin-interacting protein N-terminus,	hypothetical protein PDIP_86620
PDIG_37120	-1.64	PF08616,PF07792,	SPA,Afi1,	Stabilization of polarity axis,Docking domain of Afi1 for Arf3 in vesicle trafficking,	hypothetical protein PDIP_83710
PDIG_14430	-1.63	PF13516,	LRR_6,	Leucine Rich repeat,	DNA repair protein Rad7, protein
PDIG_40370	-1.63	PF01070,PF00173,	FMN_dh,Cyt-b5,	FMN-dependent dehydrogenase,Cytochrome b5-like Heme/Steroid binding domain,	Mitochondrial cytochrome b2, putative
PDIG_74190	-1.63	PF00171,	Aldedh,	Aldehyde dehydrogenase family,	Methylmalonate-semialdehyde dehydrogenase, putative

PDIG_81510	-1.62	PF04516,	CP2,	CP2 transcription factor,	CP2 transcription factor, putative
PDIG_29080	-1.62	PF00152,PF09924,	tRNA-synt_2,DUF2156,	tRNA synthetases class II (D, K and N),Uncharacterised conserved protein (DUF2156),	Aspartyl-tRNA synthetase, cytoplasmic
PDIG_78620	-1.62	PF03443,	Glyco_hydro_61,	Glycosyl hydrolase family 61,	Endoglucanase, putative
PDIG_18550	-1.62	PF12923,	RRP7,	Ribosomal RNA-processing protein 7 (RRP7),	Ribosomal small subunit assembly protein, putative
PDIG_16580	-1.62	PF05192,PF05188,PF00488,PF01624,PF05190,	MutS_III,MutS_II,MutS_V,MutS_I,MutS_IV,	MutS domain III,MutS domain II,MutS domain V,MutS domain I,MutS family domain IV,	DNA mismatch repair protein Msh6, putative
PDIG_42680	-1.62				hypothetical protein PDIG_42680
PDIG_31340	-1.62	PF05183,	RdRP,	RNA dependent RNA polymerase,	RNA-directed RNA polymerase, putative
PDIG_10910	-1.61	PF01612,	DNA_pol_A_exo1,	3'-5' exonuclease,	hypothetical protein PDIP_82420
PDIG_82370	-1.61	PF07539,	DRIM,	Down-regulated in metastasis,	HEAT repeat protein (DRIM), putative
PDIG_73550	-1.61	PF15446,PF00271,PF00176,	zf-PHD-like,Helicase_C,SNF2_N,	PHD/FYVE-zinc-finger like domain,Helicase conserved C-terminal domain,SNF2 family N-terminal domain,	Chromatin remodeling complex subunit (Chd3), putative
PDIG_20520	-1.60	PF07748,PF01074,PF09261,	Glyco_hydro_38C,Glyco_hydro_38,Alpha-mann_mid,	Glycosyl hydrolases family 38 C-terminal domain,Glycosyl hydrolases family 38 N-terminal domain,Alpha mannosidase middle domain,	Alpha-mannosidase
PDIG_82270	-1.60	PF01182,	Glucosamine_iso,	Glucosamine-6-phosphate isomerases/6-phosphogluconolactonas	Glucosamine-6-phosphate deaminase, putative

				e,	
PDIG_87290	-1.59	PF06371,	Drf_GBD,	Diaphanous GTPase-binding Domain,	hypothetical protein PDIG_87290
PDIG_52850	-1.59	PF00641,PF00929,PF00076,	zf-RanBP,RNase_T,RRM_1,	Zn-finger in Ran binding protein and others,Exonuclease,RNA recognition motif. (a.k.a. RRM, RBD, or RNP domain),	RNA binding protein (Arp), putative
PDIG_82150	-1.59	PF04082,	Fungal_trans,	Fungal specific transcription factor domain,	Pc22g10180 [Penicillium chrysogenum Wisconsin 54-1255]
PDIG_06550	-1.59				Alcohol dehydrogenase 1
PDIG_85630	-1.59	PF00614,	PLDc,	Phospholipase D Active site motif,	Pc20g01680 [Penicillium chrysogenum Wisconsin 54-1255]
PDIG_02470	-1.58	PF13450,PF01593,	NAD_binding_8,Amino_oxidase,	NAD(P)-binding Rossmann-like domain,Flavin containing amine oxidoreductase,	hypothetical protein PDIG_02470
PDIG_86190	-1.58	PF00628,PF13621,	PHD,Cupin_8,	PHD-finger,Cupin-like domain,	hypothetical protein PDIG_86190
PDIG_72200	-1.58	PF00071,	Ras,	Ras family,	Rho GTPase Rho4, putative
PDIG_63970	-1.58	PF10330,	Stb3,	Putative Sin3 binding protein,	hypothetical protein PDIP_73320
PDIG_05440	-1.57	PF00076,	RRM_1,	RNA recognition motif. (a.k.a. RRM, RBD, or RNP domain),	Nucleolin protein Nsr1, putative
PDIG_07920	-1.57				hypothetical protein PDIP_35960
PDIG_88020	-1.57	PF00400,PF12937,	WD40,F-box-like,	WD domain, G-beta repeat,F-box-like,	hypothetical protein PDIG_88020
PDIG_08070	-1.56				hypothetical protein PDIG_45040
PDIG_44960	-1.56				hypothetical protein PDIG_45040
PDIG_45040	-1.56				hypothetical protein PDIG_45040

PDIG_68810	-1.56	PF11915,	DUF3433,	Protein of unknown function (DUF3433),	hypothetical protein PDIG_68810
PDIG_82300	-1.55	PF01979,	Amidohydro_1,	Amidohydrolase family,	N-acetylglucosamine-6-phosphate deacetylase (NagA), putative
PDIG_07650	-1.55	PF17123,PF00498,	zf-RING_11,FHA,	RING-like zinc finger,FHA domain,	hypothetical protein PDIP_81770
PDIG_00910	-1.55	PF02142,PF01808,	MGS,AICARFT_IMPCHas,	MGS-like domain,AICARFT/IMPCHase bienzyme,	Phosphoribosylaminoimidazolecarboxamide formyltransferase/IMP cyclohydrolase
PDIG_08680	-1.55				hypothetical protein PDIG_08680
PDIG_30860	-1.54	PF13923,PF00176,PF08797,PF00271,	zf-C3HC4_2,SNF2_N,HIRAN,Helicase_C,	Zinc finger, C3HC4 type (RING finger),SNF2 family N-terminal domain,HIRAN domain,Helicase conserved C-terminal domain,	SNF2 family helicase, putative
PDIG_32920	-1.54	PF06027,	SLC35F,	Solute carrier family 35,	hypothetical protein PDIP_52480
254 elements included exclusively in "∆PdsreB-up":					
PDIG_07400	1.58				hypothetical protein PDIG_07400
PDIG_72750	1.58				hypothetical protein PDIG_72750
PDIG_75630	1.59	PF00141,	peroxidase,	Peroxidase,	Putative heme-binding peroxidase
PDIG_70050	1.60				putative kinetochore protein spc24
PDIG_26230	1.60	PF00241,	Cofilin_ADF,	Cofilin/tropomyosin-type actin-binding protein,	GMF family protein
PDIG_07860	1.60	PF01370,	Epimerase,	NAD dependent epimerase/dehydratase family,	hypothetical protein PDIP_35900
PDIG_53930	1.60	PF04573,	SPC22,	Signal peptidase subunit,	Microsomal signal peptidase subunit (Gp23), putative
PDIG_69720	1.60	PF04082,	Fungal_trans,	Fungal specific transcription factor	C2H2 transcription factor, putative

				domain,	
PDIG_27990	1.61	PF07955,	DUF1687,	Protein of unknown function (DUF1687),	hypothetical protein PDIP_62430
PDIG_35820	1.61	PF01920,	Prefoldin_2,	Prefoldin subunit,	Prefoldin subunit 4, putative
PDIG_67530	1.61				NADH-ubiquinone oxidoreductase 14 kDa subunit, putative
PDIG_65080	1.62	PF00179,	UQ_con,	Ubiquitin-conjugating enzyme,	Ubiquitin conjugating enzyme (UbcB), putative
PDIG_72090	1.62	PF00137,	ATP-synt_C,	ATP synthase subunit C,	V-ATPase proteolipid subunit Ppa1, putative
PDIG_19790	1.62	PF02046,	COX6A,	Cytochrome c oxidase subunit VIa,	Cytochrome c oxidase polypeptide VIa
PDIG_50220	1.62	PF00153,	Mito_carr,	Mitochondrial carrier protein,	hypothetical protein PDIG_50220
PDIG_67170	1.63	PF00583,	Acetyltransf_1,	Acetyltransferase (GNAT) family,	Acetyltransferase, GNAT family, putative
PDIG_23150	1.63				hypothetical protein PDIG_23150
PDIG_29180	1.64	PF00887,	ACBP,	Acyl CoA binding protein,	Acyl CoA binding protein family
PDIG_33400	1.65				hypothetical protein PDIP_52980
PDIG_30540	1.65	PF01165,	Ribosomal_S21,	Ribosomal protein S21,	hypothetical protein PDIP_64920
PDIG_32160	1.65	PF00137,	ATP-synt_C,	ATP synthase subunit C,	vacuolar ATPase proteolipid subunit c, putative [Neosartorya fischeri NRRL 181]
PDIG_23750	1.65	PF01423,	LSM,	LSM domain,	Small nuclear ribonucleoprotein-associated protein B
PDIG_49540	1.65	PF01124,	MAPEG,	MAPEG family,	hypothetical protein PDIG_49540
PDIG_75250	1.65	PF02036,	SCP2,	SCP-2 sterol transfer family,	Pc22g01300 [Penicillium chrysogenum Wisconsin 54-1255]
PDIG_05290	1.65	PF02630,	SCO1-SenC,	SCO1/SenC,	Mitochondrial metallochaperone Sco1, putative
PDIG_64790	1.66	PF10775,	ATP_sub_h,	ATP synthase complex subunit h,	Mitochondrial F1F0 ATP synthase subunit Atp14, putative
PDIG_65340	1.66	PF09598,	Stm1_N,	Stm1,	Telomere and ribosome associated protein Stm1, putative

PDIG_10360	1.66	PF05047,	L51_S25_CI-B8,	Mitochondrial ribosomal protein L51 / S25 / CI-B8 domain,	Mitochondrial ribosomal protein L43, putative
PDIG_18680	1.67	PF01042,	Ribonuc_L-PSP,	Endoribonuclease L-PSP,	L-PSP endoribonuclease family protein (Hmf1), putative
PDIG_53840	1.68	PF15932,	DUF4748,	Domain of unknown function (DUF4748),	hypothetical protein PDIG_53840
PDIG_66600	1.68	PF11712,	Vma12,	Endoplasmic reticulum-based factor for assembly of V-ATPase,	hypothetical protein PDIP_75900
PDIG_67820	1.69				hypothetical protein PDIP_77130
PDIG_09550	1.69				hypothetical protein PDIP_37580
PDIG_85980	1.69	PF01522,	Polysacc_deac_1,	Polysaccharide deacetylase,	hypothetical protein PDIP_47090 [Penicillium digitatum Pd1]
PDIG_52730	1.70	PF01894,	UPF0047,	Uncharacterised protein family UPF0047,	hypothetical protein PDIG_52730
PDIG_67500	1.70	PF01250,	Ribosomal_S6,	Ribosomal protein S6,	37S ribosomal protein Mrp17
PDIG_28020	1.71				Pc22g23020 [Penicillium chrysogenum Wisconsin 54-1255]
PDIG_29820	1.71	PF01063,	Aminotran_4,	Amino-transferase class IV,	Branched-chain-amino-acid aminotransferase
PDIG_46130	1.71	PF13233,	Complex1_LYR_2,	Complex1_LYR-like,	NADH-ubiquinone oxidoreductase B14 subunit, putative
PDIG_84300	1.71	PF00462,	Glutaredoxin,	Glutaredoxin,	Glutaredoxin Grx5, putative
PDIG_73200	1.72	PF08511,	COQ9,	COQ9,	hypothetical protein PDIG_73200
PDIG_40010	1.73	PF01910,	Thiamine_BP,	Thiamine-binding protein,	Cell wall biogenesis protein Ecm15, putative
PDIG_80080	1.73	PF00025,	Arf,	ADP-ribosylation factor family,	hypothetical protein PDIP_28460 [Penicillium digitatum Pd1]
PDIG_79360	1.73	PF09206,PF	ArabFuran-catal,AbfB,	Alpha-L-arabinofuranosidase B,	Alfa-L-arabinofuranosidase

		05270,		catalytic,Alpha-L-arabinofuranosidase B (ABFB) domain,	
PDIG_76500	1.74	PF02627,	CMD,	Carboxymuconolactone decarboxylase family,	4-carboxymuconolactone decarboxylase family protein
PDIG_64260	1.74	PF13664,	DUF4149,	Domain of unknown function (DUF4149),	hypothetical protein PDIP_73600
PDIG_69260	1.75	PF09784,	L31,	Mitochondrial ribosomal protein L31,	hypothetical protein PDIP_78550
PDIG_85900	1.75	PF00583,	Acetyltransf_1,	Acetyltransferase (GNAT) family,	hypothetical protein PDIG_85900
PDIG_47690	1.76				hypothetical protein PDIP_57070
PDIG_19880	1.77				hypothetical protein PDIG_19880
PDIG_78930	1.77	PF11976,	Rad60-SLD,	Ubiquitin-2 like Rad60 SUMO-like,	Ubiquitin-like modifier SUMO, putative
PDIG_15390	1.77	PF00254,	FKBP_C,	FKBP-type peptidyl-prolyl cis-trans isomerase,	FK506-binding protein 1B
PDIG_85250	1.78	PF01423,	LSM,	LSM domain,	Small nuclear ribonucleoprotein SmE, putative
PDIG_80020	1.78	PF02077,	SURF4,	SURF4 family,	COPII-coated vesicle protein SurF4/Erv29, putative
PDIG_66850	1.78	PF00152,	tRNA-synt_2,	tRNA synthetases class II (D, K and N),	Asparaginyl-tRNA synthetase Slm5, putative
PDIG_20290	1.79	PF10780,	MRP_L53,	39S ribosomal protein L53/MRP-L53,	hypothetical protein PDIG_20290
PDIG_37680	1.79	PF06179,	Med22,	Surfeit locus protein 5 subunit 22 of Mediator complex,	hypothetical protein PDIP_84270
PDIG_83940	1.79	PF02953,	zf-Tim10_DDP,	Tim10/DDP family zinc finger,	Mitochondrial intermembrane space translocase subunit Tim13, putative
PDIG_84820	1.80	PF00288,PF08544,	GHMP_kinases_N,GHMP_kinases_C,	GHMP kinases N terminal domain,GHMP kinases C terminal,	Homoserine kinase
PDIG_19180	1.80	PF01729,PF02749,	QRPTase_C,QRPTase_N,	Quinolinate phosphoribosyl transferase, C-terminal	Nicotinate-nucleotide pyrophosphorylase

				domain,Quinolinate phosphoribosyl transferase, N-terminal domain,	
PDIG_61150	1.81	PF08520,	DUF1748,	Fungal protein of unknown function (DUF1748),	Pc18g05990 [Penicillium chrysogenum Wisconsin 54-1255]
PDIG_30640	1.81	PF02466,	Tim17,	Tim17/Tim22/Tim23/Pmp24 family,	Mitochondrial inner membrane translocase subunit (TIM17), putative
PDIG_85280	1.83	PF13499,	EF-hand_7,	EF-hand domain pair,	Cytokinesis EF-hand protein Cdc4, putative
PDIG_05890	1.83	PF02845,	CUE,	CUE domain,	AMFR protein, putative
PDIG_46230	1.83				hypothetical protein PDIG_46230
PDIG_62610	1.83	PF01199,	Ribosomal_L34e,	Ribosomal protein L34e,	Ribosomal protein L34 protein, putative
PDIG_73230	1.83	PF02466,	Tim17,	Tim17/Tim22/Tim23/Pmp24 family,	NADH-ubiquinone oxidoreductase 213 kDa subunit
PDIG_42430	1.83	PF00076,	RRM_1,	RNA recognition motif. (a.k.a. RRM, RBD, or RNP domain),	hypothetical protein PDIG_42430
PDIG_88080	1.83	PF02320,	UCR_hinge,	Ubiquinol-cytochrome C reductase hinge protein,	Cytochrome b-c1 complex subunit 6
PDIG_79060	1.83	PF02792,	Mago_nashi,	Mago nashi protein,	Pc13g13540 [Penicillium chrysogenum Wisconsin 54-1255]
PDIG_77200	1.84				NADH-ubiquinone oxidoreductase 12 kDa subunit, putative
PDIG_86470	1.84				Transposable element tc3 transposase, putative
PDIG_01950	1.84	PF02271,	UCR_14kD,	Ubiquinol-cytochrome C reductase complex 14kD subunit,	Ubiquinol-cytochrome c reductase complex 14 kDa protein
PDIG_11040	1.85	PF03656,	Pam16,	Pam16,	Cochaperone Pam16
PDIG_05950	1.85	PF03061,	4HBT,	Thioesterase superfamily,	Thioesterase family protein, putative
PDIG_57160	1.86	PF00389,PF 00237,PF02	2-Hacid_dh,Ribosomal_L2 2,2-Hacid_dh_C,	D-isomer specific 2-hydroxyacid dehydrogenase, catalytic	hypothetical protein PDE_00303 [Penicillium oxalicum 114-2]

		826,		domain,Ribosomal protein L22p/L17e,D-isomer specific 2-hydroxyacid dehydrogenase, NAD binding domain,	
PDIG_84580	1.86	PF01725,	Ham1p_like,	Ham1 family,	Non-canonical purine NTP pyrophosphatase, rdgB/HAM1 family
PDIG_51720	1.87				Pc16g08840 [Penicillium chrysogenum Wisconsin 54-1255]
PDIG_38570	1.87	PF02970,	TBCA,	Tubulin binding cofactor A,	hypothetical protein PDIP_85210
PDIG_54750	1.87	PF05032,	Spo12,	Spo12 family,	hypothetical protein PDIG_54750
PDIG_81940	1.89				hypothetical protein PDIG_81940
PDIG_47110	1.89	PF00153,	Mito_carr,	Mitochondrial carrier protein,	Mitochondrial folate carrier protein Flx1, putative
PDIG_67930	1.90	PF00397,	WW,	WW domain,	hypothetical protein PDIP_77230
PDIG_69710	1.90	PF00903,	Glyoxalase,	Glyoxalase/Bleomycin resistance protein/Dioxygenase superfamily,	Lactoylglutathione lyase (Glo1), putative
PDIG_51260	1.92	PF02953,	zf-Tim10_DDP,	Tim10/DDP family zinc finger,	Mitochondrial intermembrane space translocase subunit Tim10, putative
PDIG_43840	1.92	PF04847,	Calcipressin,	Calcipressin,	Calcineurin binding protein, putative
PDIG_12000	1.93	PF00153,	Mito_carr,	Mitochondrial carrier protein,	hypothetical protein PDIP_38220
PDIG_63330	1.95	PF05433,	Rick_17kDa_Anti,	Glycine zipper 2TM domain,	Pc18g03250 [Penicillium chrysogenum Wisconsin 54-1255]
PDIG_43890	1.95	PF01025,	GrpE,	GrpE,	hypothetical protein PDIG_43890
PDIG_70040	1.95	PF10356,	DUF2034,	Protein of unknown function (DUF2034),	hypothetical protein PDIP_79340
PDIG_29880	1.96	PF01521,	Fe-S_biosyn,	Iron-sulphur cluster biosynthesis,	Iron-sulfur cluster assembly accessory protein Isa2, putative

PDIG_53490	1.96	PF00687,	Ribosomal_L1,	Ribosomal protein L1p/L10e family,	Pc21g15750 [Penicillium chrysogenum Wisconsin 54-1255]
PDIG_54910	1.97	PF02805,PF 00165,	Ada_Zn_binding,HTH_Ara C,	Metal binding domain of Ada,Bacterial regulatory helix-turn-helix proteins, AraC family,	DNA repair and transcription factor Ada, putative
PDIG_60830	1.97				hypothetical protein PDIG_60830
PDIG_10230	1.97				Cytochrome c oxidase assembly protein Cox19, putative
PDIG_33960	1.97	PF00179,	UQ_con,	Ubiquitin-conjugating enzyme,	Ubiquitin carrier protein
PDIG_88200	1.98				hypothetical protein PDIG_88200
PDIG_00660	1.99	PF00457,	Glyco_hydro_11,	Glycosyl hydrolases family 11,	putative xylanase 2 [Penicillium digitatum]
PDIG_63700	2.00				hypothetical protein PDIP_73050
PDIG_70530	2.00	PF13649,PF 07690,	Methyltransf_25,MFS_1,	Methyltransferase domain,Major Facilitator Superfamily,	hypothetical protein W97_00653 [Coniosporium apollinis CBS 100218]
PDIG_09370	2.01	PF00708,	Acylphosphatase,	Acylphosphatase,	hypothetical protein PDIP_37430
PDIG_62630	2.02	PF01215,	COX5B,	Cytochrome c oxidase subunit Vb,	hypothetical protein PDIP_72010
PDIG_84800	2.03	PF12678,	zf-rbx1,	RING-H2 zinc finger domain,	Ubiquitin ligase subunit HrtA, putative
PDIG_75050	2.04	PF01230,	HIT,	HIT domain,	hypothetical protein PDIG_75050
PDIG_54300	2.04	PF02230,	Abhydrolase_2,	Phospholipase/Carboxylesterase,	Phospholipase/Carboxylesterase superfamily
PDIG_61090	2.05	PF06687,	SUR7,	SUR7/PalI family,	Integral membrane protein
PDIG_74150	2.05	PF08571,	Yos1,	Yos1-like,	hypothetical protein PDIG_74150
PDIG_47040	2.05	PF02866,PF 00056,	Ldh_1_C,Ldh_1_N,	lactate/malate dehydrogenase, alpha/beta C-terminal domain,lactate/malate dehydrogenase, NAD binding domain,	Malate dehydrogenase, NAD-dependent
PDIG_83680	2.05	PF07896,	DUF1674,	Protein of unknown function	hypothetical protein PDIP_89180

				(DUF1674),	
PDIG_83950	2.05	PF03388,	Lectin_leg-like,	Legume-like lectin family,	Lectin family integral membrane protein, putative
PDIG_79220	2.05				Coatomer subunit epsilon, putative [Penicillium digitatum Pd1]
PDIG_15790	2.06	PF00300,	His_Phos_1,	Histidine phosphatase superfamily (branch 1),	GPI anchored protein, putative
PDIG_31150	2.06	PF01248,	Ribosomal_L7Ae,	Ribosomal protein L7Ae/L30e/S12e/Gadd45 family,	Pc21g18200 [Penicillium chrysogenum Wisconsin 54-1255]
PDIG_34640	2.06	PF02629,PF00549,	CoA_binding,Ligase_CoA,	CoA binding domain,CoA-ligase,	succinyl-CoA ligase alpha-chain [Uncinocarpus reesii 1704]
PDIG_04270	2.07				hypothetical protein PDIG_04270
PDIG_05460	2.07				hypothetical protein PDIG_05460
PDIG_00280	2.08	PF00076,	RRM_1,	RNA recognition motif. (a.k.a. RRM, RBD, or RNP domain),	hypothetical protein PDIG_00280
PDIG_17530	2.08				hypothetical protein PDIP_55420
PDIG_05210	2.08				hypothetical protein PDIG_05210
PDIG_35210	2.09				hypothetical protein PDIP_54760
PDIG_72820	2.09	PF08534,	Redoxin,	Redoxin,	hypothetical protein FOXB_13058 [Fusarium oxysporum Fo5176]
PDIG_50710	2.09	PF13850,PF07970,	ERGIC_N,COPIIcoated_ERV,	Endoplasmic Reticulum-Golgi Intermediate Compartment (ERGIC),Endoplasmic reticulum vesicle transporter,	COPII-coated vesicle membrane protein Erv46, putative
PDIG_55790	2.11	PF05328,	CybS,	CybS, succinate dehydrogenase cytochrome B small subunit,	Succinate dehydrogenase subunit CybS, putative
PDIG_32840	2.11	PF00436,	SSB,	Single-strand binding protein family,	SsDNA binding protein, putative

PDIG_23740	2.12	PF03650,	MPC,	Uncharacterised protein family (UPF0041),	hypothetical protein PDIG_23740
PDIG_26070	2.12				hypothetical protein PDIP_60550
PDIG_49680	2.14	PF00107,PF08240,	ADH_zinc_N,ADH_N,	Zinc-binding dehydrogenase,Alcohol dehydrogenase GroES-like domain,	Zinc-binding alcohol dehydrogenase, putative
PDIG_19650	2.14	PF00179,	UQ_con,	Ubiquitin-conjugating enzyme,	Pc20g07930 [Penicillium chrysogenum Wisconsin 54-1255]
PDIG_61790	2.15	PF00160,	Pro_isomerase,	Cyclophilin type peptidyl-prolyl cis-trans isomerase/CLD,	peptidyl-prolyl cis-trans isomerase [Aspergillus niger CBS 513.88]
PDIG_58440	2.15	PF04828,	GFA,	Glutathione-dependent formaldehyde-activating enzyme,	hypothetical protein PDIP_67920
PDIG_02190	2.16				hypothetical protein PDIG_45040
PDIG_67260	2.18	PF04124,	Dor1,	Dor1-like family,	Dor1-like family protein
PDIG_78150	2.18	PF02939,	UcrQ,	UcrQ family,	Ubiquinol-cytochrome C reductase complex subunit UcrQ, putative
PDIG_50160	2.18	PF04627,	ATP-synt_Eps,	Mitochondrial ATP synthase epsilon chain,	hypothetical protein PDIG_50160
PDIG_03750	2.19	PF08240,PF00107,	ADH_N,ADH_zinc_N,	Alcohol dehydrogenase GroES-like domain,Zinc-binding dehydrogenase,	hypothetical protein PDIG_03750
PDIG_00950	2.20	PF00160,	Pro_isomerase,	Cyclophilin type peptidyl-prolyl cis-trans isomerase/CLD,	Peptidyl-prolyl cis-trans isomerase B
PDIG_22070	2.21	PF13472,	Lipase_GDSL_2,	GDSL-like Lipase/Acylhydrolase family,	Rhamnogalacturonan acetylesterase RgaE
PDIG_26320	2.21	PF14529,	Exo_endo_phos_2,	Endonuclease-reverse transcriptase,	hypothetical protein PDIG_26320
PDIG_74790	2.21	PF12585,	DUF3759,	Protein of unknown function (DUF3759),	hypothetical protein PDIG_74790

PDIG_19970	2.21	PF09849,	DUF2076,	Uncharacterized protein conserved in bacteria (DUF2076),	hypothetical protein PDIG_19970
PDIG_60120	2.22				hypothetical protein PDIG_60120
PDIG_09340	2.22				hypothetical protein PDIP_37460
PDIG_37240	2.22	PF02823,	ATP-synt_DE_N,	ATP synthase, Delta/Epsilon chain, beta-sandwich domain,	ATP synthase delta chain, mitochondrial, putative
PDIG_87230	2.23	PF15879,	MWFE,	NADH-ubiquinone oxidoreductase MWFE subunit,	hypothetical protein PDIG_87230
PDIG_27470	2.23	PF00179,	UQ_con,	Ubiquitin-conjugating enzyme,	Pc22g23780 [Penicillium chrysogenum Wisconsin 54-1255]
PDIG_43780	2.24	PF00034,	Cytochrom_C,	Cytochrome c,	Cytochrome c
PDIG_82620	2.24				hypothetical protein PDIP_86420
PDIG_36970	2.24	PF07297,	DPM2,	Dolichol phosphate-mannose biosynthesis regulatory protein (DPM2),	Dolichol phosphate-mannose biosynthesis regulatory protein Dpm2, putative
PDIG_67690	2.24				Mitochondrial zinc maintenance protein 1, mitochondrial
PDIG_11920	2.25				Putative ATP synthase protein 9
PDIG_90870	2.28				hypothetical protein PDIG_90870
PDIG_38120	2.28	PF00238,	Ribosomal_L14,	Ribosomal protein L14p/L23e,	50S ribosomal protein L14
PDIG_69460	2.29				hypothetical protein PDIP_78750
PDIG_64700	2.29	PF00137,	ATP-synt_C,	ATP synthase subunit C,	Vacuolar ATP synthase 16 kDa proteolipid subunit, putative
PDIG_69770	2.30	PF09347,	DUF1989,	Domain of unknown function (DUF1989),	hypothetical protein PDIP_79060
PDIG_62650	2.32				hypothetical protein PDIG_62650

PDIG_28090	2.33	PF03650,	MPC,	Uncharacterised protein family (UPF0041),	hypothetical protein PDIP_62530
PDIG_38190	2.34	PF05705,	DUF829,	Eukaryotic protein of unknown function (DUF829),	hypothetical protein PDIP_84770
PDIG_23520	2.34				hypothetical protein PDIG_23520
PDIG_49310	2.34	PF00383,	dCMP_cyt_deam_1,	Cytidine and deoxycytidylate deaminase zinc-binding region,	Cytidine deaminase, putative [Penicillium digitatum Pd1]
PDIG_56350	2.35				hypothetical protein PDIP_65910
PDIG_60170	2.36				hypothetical protein PDIP_69580
PDIG_80500	2.38	PF02136,	NTF2,	Nuclear transport factor 2 (NTF2) domain,	Nuclear transport factor NTF-2, putative
PDIG_61630	2.41				Mitochondrial import inner membrane translocase subunit tim14
PDIG_20900	2.41				hypothetical protein PDIG_20900
PDIG_23210	2.43	PF03061,	4HBT,	Thioesterase superfamily,	PaaI_thioesterase family protein, putative
PDIG_24910	2.43	PF06825,	HSBP1,	Heat shock factor binding protein 1,	hypothetical protein PDIP_59390
PDIG_87280	2.43				hypothetical protein PDIG_87280
PDIG_22780	2.45	PF01648,	ACPS,	4'-phosphopantetheinyl transferase superfamily,	Phosphopantetheinyl transferase PptB
PDIG_51070	2.45	PF03911,	Sec61_beta,	Sec61beta family,	Translocon protein Sec61beta, putative
PDIG_29610	2.46	PF09796,	QCR10,	Ubiquinol-cytochrome-c reductase complex subunit (QCR10),	Pc22g20870 [Penicillium chrysogenum Wisconsin 54-1255]
PDIG_89920	2.46				hypothetical protein PDIG_89920
PDIG_56030	2.46	PF08881,	CVNH,	CVNH domain,	Cyanovirin-N family protein
PDIG_22160	2.47				Pc13g15770 [Penicillium chrysogenum Wisconsin 54-1255]

PDIG_18120	2.47				TOM core complex subunit Tom6, putative
PDIG_15330	2.48	PF00459,	Inositol_P,	Inositol monophosphatase family,	Inositol monophosphatase QutG, putative
PDIG_28050	2.50	PF07960,	CBP4,	CBP4,	hypothetical protein PDIP_62490
PDIG_32700	2.51	PF05922,	Inhibitor_I9,	Peptidase inhibitor I9,	hypothetical protein PDIP_52270
PDIG_80550	2.57	PF09072,	TMA7,	Translation machinery associated TMA7,	hypothetical protein PDIG_80550
PDIG_69780	2.59				hypothetical protein PDIP_79070
PDIG_12550	2.59				Transcriptional elongation regulator Elc1/Elongin C, putative
PDIG_42740	2.59	PF12554,	MOZART1,	Mitotic-spindle organizing gamma-tubulin ring associated,	Mitotic-spindle organizing protein 1
PDIG_66450	2.60	PF10247,	Romo1,	Reactive mitochondrial oxygen species modulator 1,	Pc12g16370 [Penicillium chrysogenum Wisconsin 54-1255]
PDIG_54690	2.60	PF05071,	NDUFA12,	NADH ubiquinone oxidoreductase subunit NDUFA12,	NADH-ubiquinone oxidoreductase subunit B17.2, putative
PDIG_34660	2.63				hypothetical protein PDIP_54230
PDIG_05420	2.68				Cytochrome c oxidase copper chaperone Cox17, putative
PDIG_72900	2.69				hypothetical protein PDIG_72900
PDIG_36510	2.71	PF00544,	Pec_lyase_C,	Pectate lyase,	putative pectate lyase 2 [Penicillium digitatum]
PDIG_28080	2.78	PF05047,	L51_S25_CI-B8,	Mitochondrial ribosomal protein L51 / S25 / CI-B8 domain,	NADH-ubiquinone oxidoreductase 105 kDa subunit
PDIG_07350	2.79				hypothetical protein PDIG_07350
PDIG_62790	2.81	PF04568,	IATP,	Mitochondrial ATPase inhibitor, IATP,	Mitochondrial ATPase inhibitor, putative
PDIG_07170	2.85				hypothetical protein PDIG_07170

PDIG_43930	2.87	PF02297,	COX6B,	Cytochrome oxidase c subunit VIb,	unnamed protein product [Aspergillus niger]
PDIG_67090	2.90	PF05254,	UPF0203,	Uncharacterised protein family (UPF0203),	hypothetical protein AKAW_03065 [Aspergillus kawachii IFO 4308]
PDIG_08190	2.91	PF00171,	Aldedh,	Aldehyde dehydrogenase family,	Delta-1-pyrroline-5-carboxylate dehydrogenase PrnC
PDIG_25920	2.93	PF00203,	Ribosomal_S19,	Ribosomal protein S19,	Pc21g06330 [Penicillium chrysogenum Wisconsin 54-1255]
PDIG_74280	2.96				LYR family protein
PDIG_84540	2.98				hypothetical protein PDIG_84540
PDIG_48970	3.01	PF01200,	Ribosomal_S28e,	Ribosomal protein S28e,	hypothetical protein ASPNIDRAFT_191806 [Aspergillus niger ATCC 1015]
PDIG_21110	3.03				hypothetical protein [Mycobacterium tuberculosis]
PDIG_23880	3.05	PF01781,	Ribosomal_L38e,	Ribosomal L38e protein family,	60S ribosomal protein L38, putative
PDIG_64270	3.07				hypothetical protein PDIP_73610
PDIG_59490	3.09				60S acidic ribosomal protein P1
PDIG_88230	3.11				hypothetical protein PDIG_88230
PDIG_27360	3.17				hypothetical protein PDIP_61800
PDIG_22800	3.21	PF00254,	FKBP_C,	FKBP-type peptidyl-prolyl cis-trans isomerase,	FKBP-type peptidyl-prolyl isomerase, putative
PDIG_74880	3.22				hypothetical protein PDIG_74880
PDIG_37250	3.27	PF01779,	Ribosomal_L29e,	Ribosomal L29e protein family,	hypothetical protein AKAW_01563 [Aspergillus kawachii IFO 4308]
PDIG_71550	3.28	PF03131,	bZIP_Maf,	bZIP Maf transcription factor,	hypothetical protein PDIP_80850
PDIG_26090	3.30				hypothetical protein PDIP_60570
PDIG_37860	3.33	PF04758,	Ribosomal_S30,	Ribosomal protein S30,	Pc13g03870 [Penicillium chrysogenum Wisconsin 54-1255]
PDIG_08320	3.46				hypothetical protein PDIP_36340

PDIG_77680	3.47	PF01263,	Aldose_epim,	Aldose 1-epimerase,	Aldose 1-epimerase, putative
PDIG_40810	3.49				hypothetical protein PDIP_85730
PDIG_26750	3.50	PF09813,	Coiled-coil_56,	Coiled-coil domain-containing protein 56,	Pc21g05250 [Penicillium chrysogenum Wisconsin 54-1255]
PDIG_41130	3.52	PF00179,	UQ_con,	Ubiquitin-conjugating enzyme,	hypothetical protein PDIP_86050
PDIG_38590	3.54	PF00240,	ubiquitin,	Ubiquitin family,	NEDD8-like protein (RubA), putative
PDIG_M0010	3.56	PF00115,	COX1,	Cytochrome C and Quinol oxidase polypeptide I,	cytochrome c oxidase subunit 1 (mitochondrion) [Penicillium digitatum]
PDIG_30390	3.59				hypothetical protein PDIP_64770
PDIG_39160	3.65	PF05365,	UCR_UQCRX_QCR9,	Ubiquinol-cytochrome C reductase, UQCRX/QCR9 like,	hypothetical protein PDIG_39160
PDIG_22770	3.65	PF11022,	DUF2611,	Protein of unknown function (DUF2611),	hypothetical protein PDIG_22770
PDIG_40040	3.76	PF02238,	COX7a,	Cytochrome c oxidase subunit VII,	hypothetical protein PDIG_40040
PDIG_09840	3.77	PF11720,	Inhibitor_I78,	Peptidase inhibitor I78 family,	hypothetical protein PDIP_39880
PDIG_00510	3.80	PF01423,	LSM,	LSM domain,	Small nuclear ribonucleoprotein SmG, putative [Penicillium digitatum Pd1]
PDIG_30800	3.81				hypothetical protein PDIP_65180
PDIG_90340	4.05				hypothetical protein PDIG_90340
PDIG_M0040	4.12	PF00116,PF02790,	COX2,COX2_TM,	Cytochrome C oxidase subunit II, periplasmic domain,Cytochrome C oxidase subunit II, transmembrane domain,	cytochrome c oxidase subunit 2 (mitochondrion) [Penicillium digitatum]
PDIG_76210	4.13				Cell wall protein PhiA
PDIG_14270	4.18	PF06624,	RAMP4,	Ribosome associated membrane protein RAMP4,	hypothetical protein PDIG_14270

PDIG_76730	4.19	PF05699,	Dimer_Tnp_hAT,	hAT family C-terminal dimerisation region,	hypothetical protein PDIG_82610
PDIG_41420	4.19	PF05699,	Dimer_Tnp_hAT,	hAT family C-terminal dimerisation region,	hypothetical protein PDIG_82610
PDIG_90310	4.19	PF05699,	Dimer_Tnp_hAT,	hAT family C-terminal dimerisation region,	hypothetical protein PDIG_82610
PDIG_39960	4.19	PF05699,	Dimer_Tnp_hAT,	hAT family C-terminal dimerisation region,	hypothetical protein PDIG_82610
PDIG_64730	4.22				hypothetical protein [Mycobacterium tuberculosis]
PDIG_04740	4.33	PF00584,	SecE,	SecE/Sec61-gamma subunits of protein translocation complex,	Protein translocation complex subunit Sss1, putative
PDIG_51480	4.51				hypothetical protein PDIG_51480
PDIG_30760	4.56	PF06747,	CHCH,	CHCH domain,	hypothetical protein PDIP_65140
PDIG_34050	4.61	PF04135,	Nop10p,	Nucleolar RNA-binding protein, Nop10p family,	H/ACA ribonucleoprotein complex subunit 3
PDIG_68270	4.63	PF08650,	DASH_Dad4,	DASH complex subunit Dad4,	hypothetical protein PDIP_77560
PDIG_02240	4.66				hypothetical protein PDIG_02240
PDIG_57900	4.66				hypothetical protein PDIG_57900
PDIG_67850	4.70				hypothetical protein [Mycobacterium tuberculosis]
PDIG_85120	4.77				hypothetical protein PDIG_85120
PDIG_17040	4.93	PF05699,	Dimer_Tnp_hAT,	hAT family C-terminal dimerisation region,	hypothetical protein PDIG_86160
PDIG_13090	4.93	PF05699,	Dimer_Tnp_hAT,	hAT family C-terminal dimerisation region,	hypothetical protein PDIG_86160
PDIG_14810	4.93	PF05699,	Dimer_Tnp_hAT,	hAT family C-terminal dimerisation region,	hypothetical protein PDIG_86160

PDIG_40770	4.93	PF05699,	Dimer_Tnp_hAT,	hAT family C-terminal dimerisation region,	hypothetical protein PDIG_86160
PDIG_01270	5.17	PF12907,PF 04419,	zf-met2,4F5,	Zinc-binding,4F5 protein family,	hypothetical protein PDIG_01270
PDIG_57990	5.26	PF07690,	MFS_1,	Major Facilitator Superfamily,	MFS multidrug transporter, putative
PDIG_15160	5.26				hypothetical protein PDIG_15160
PDIG_04460	5.31				hypothetical protein PDIG_04460
PDIG_08330	5.37				putative 4-hydroxyphenylpyruvate dioxygenase 1
PDIG_89590	5.57	PF00076,	RRM_1,	RNA recognition motif. (a.k.a. RRM, RBD, or RNP domain),	hypothetical protein PDIG_89590
PDIG_64680	6.37				hypothetical protein PDIG_64680
